# TGx-DDI (toxicogenomic DNA damage-inducing) biomarker validation: multi-site ring trial supporting regulatory use

**DOI:** 10.1093/toxsci/kfaf138

**Published:** 2025-10-01

**Authors:** Xiaotong Wang, Christine E Crute, Ashley Allemang, Jiri Aubrecht, Florence Burleson, Yasmin Dietz-Baum, Lena Dorsheimer, Albert J Fornace, Roland Frötschl, Ulrike Hemmann, Constance A Mitchell, Stefan Pfuhler, Andrew Williams, Lorreta Yun-Tien Lin, Syril Pettit, Carole L Yauk, Heng-Hong Li

**Affiliations:** Department of Biology, University of Ottawa, Ottawa, ON K1N 9A7, Canada; Health and Environmental Sciences Institute (HESI Global), Washington, DC 20005, United States; The Procter & Gamble Company, Mason, OH 45202, United States; Department of Oncology, Georgetown University Medical Center, Washington, DC 20057, United States; Burleson Research Technologies, Inc, Morrisville, NC 27560, United States; Research and Development, Translational Medicine Unit, Sanofi-Aventis Deutschland GmbH, Frankfurt am Main, HE 65926, Germany; Research and Development, Translational Medicine Unit, Sanofi-Aventis Deutschland GmbH, Frankfurt am Main, HE 65926, Germany; Department of Oncology, Georgetown University Medical Center, Washington, DC 20057, United States; Department of Biochemistry and Molecular & Cellular Biology, Georgetown University Medical Center, Washington, DC20057, United States; BfArM-Bundesinstitut für Arzneimittel und Medizinprodukte, Federal Institute for Drugs and Medical Devices, Bonn, NRW D-53175, Germany; Research and Development, Translational Medicine Unit, Sanofi-Aventis Deutschland GmbH, Frankfurt am Main, HE 65926, Germany; Health and Environmental Sciences Institute (HESI Global), Washington, DC 20005, United States; The Procter & Gamble Company, Mason, OH 45202, United States; Environmental Health Science and Research Bureau, Health Canada, Ottawa, ON K1A 0K9, Canada; Department of Oncology, Georgetown University Medical Center, Washington, DC 20057, United States; Health and Environmental Sciences Institute (HESI Global), Washington, DC 20005, United States; Department of Biology, University of Ottawa, Ottawa, ON K1N 9A7, Canada; Department of Oncology, Georgetown University Medical Center, Washington, DC 20057, United States; Department of Biochemistry and Molecular & Cellular Biology, Georgetown University Medical Center, Washington, DC20057, United States

**Keywords:** TGx-DDI, toxicogenomics, genotoxicity, biomarker, in vitro, validation, new approach methodologies (NAMs)

## Abstract

Standard in vitro genotoxicity assays often suffer from low specificity, leading to irrelevant positive findings that require costly in vivo follow-up studies. The TGx-DDI (Toxicogenomic DNA Damage-Inducing) transcriptomic biomarker was developed to address this limitation by identifying DNA damage-inducing compounds through gene expression profiling in human TK6 lymphoblastoid cells. To qualify TGx-DDI as a reliable, reproducible biomarker for augmenting genotoxicity hazard assessment, a multi-site ring-trial was conducted across four laboratories using 14 blinded test compounds and standardized protocols. TK6 cells were exposed to three concentrations of each compound, followed by RNA extraction and digital nucleic acid counting using the NanoString nCounter platform. A three-pronged bioinformatics approach—Nearest Shrunken Centroid Probability Analysis, Principal Component Analysis, and Hierarchical Clustering—was used to assign DDI or non-DDI classifications. TGx-DDI demonstrated 100% sensitivity, 86% specificity, and 91% accuracy in distinguishing DDI from non-DDI compounds under validated test conditions. High interlaboratory concordance was observed (agreement coefficients ≥0.61), and transcriptomic data showed strong cross-site correlation (Pearson *r* > 0.84). The biomarker reproducibly classified test agents even when conducted across study sites. These results demonstrate that TGx-DDI is a robust and reproducible transcriptomic biomarker that enhances the specificity of genotoxicity testing by distinguishing biologically relevant DNA damage responses. Its integration into genotoxicity testing strategies can support regulatory decision-making, reduce unnecessary animal use, and improve the assessment of human health risks.

Regulatory frameworks, including guidance from the U.S. Food and Drug Administration (FDA) and the International Council for Harmonisation (ICH S2(R1)), require genotoxicity data to inform risk assessments and support safe progression of drug candidates. Conventional genotoxicity testing batteries typically consist of in vitro assays including the bacterial reverse mutation test (OECD TG 471), the in vitro chromosomal aberration assay (OECD TG 473), and the in vitro micronucleus assay (OECD TG 487). Although sensitive, these assays often yield “irrelevant positives”—in vitro findings that do not reflect true in vivo genotoxic or carcinogenic potential (Kirkland et al. 2005; Avlasevich et al. 2021), necessitating resource-intensive in vivo follow-up studies and halting risk assessment and drug development. This underscores the need for more specific and mechanistically informative assays to better contextualize in vitro findings (Kirkland et al. 2005; Le Hégarat et al. 2014).

Transcriptomic biomarkers were proposed nearly two decades ago to refine genotoxicity assessments (Froetschl et al. 2025; Meier et al. 2025). These biomarkers enable high-throughput, objective profiling of cellular responses to genotoxic stressors, offering mechanistic insight into DNA damage pathways. Among these, the TGx-DDI transcriptomic biomarker has undergone the most extensive validation.

The TGx-DDI biomarker was developed to address the limitations of traditional in vitro genotoxicity assays, particularly their low specificity (Li et al. 2019). Using a toxicogenomics approach, TGx-DDI was identified from a dataset of 28 model agents with well-characterized genotoxic and nongenotoxic mechanisms in TK6 human lymphoblastoid cells (Li et al. 2015). A panel of 64 genes was identified as a reliable classifier for distinguishing DDI and non-DDI compounds (Li et al. 2015, 2017). Since its initial development, TGx-DDI has been validated across multiple laboratories, cell models, and platforms, which include Agilent and Affymetrix microarrays, NanoString nCounter, RT-qPCR, RNA-Seq, and TempO-Seq (Buick et al. 2015, 2017, 2020, 2021; Yauk et al. 2016; Cho et al. 2019; Allemang et al. 2021; Chen et al. 2022). Its high sensitivity and specificity highlight its potential as a tool for resolving equivocal results from standard genotoxicity tests and providing additional mechanistic insight into DNA damage responses. Cross-platform agreement analyses have shown strong concordance in TGx-DDI calls, with statistical measures indicating high reliability independent of the transcriptomic technology employed (Andrew Williams, Health Canada, unpublished data).

Further supporting its utility, TGx-DDI has been validated in the presence of metabolic activation (the liver microsomal S9 mix), extending its applicability to pro-genotoxicants (Buick et al. 2015; Yauk et al. 2016; Li et al. 2017; Chen et al. 2022; Fortin et al. 2023). Health Canada has integrated TGx-DDI into its GeneTox21 program, employing the biomarker for screening environmental chemicals alongside flow cytometry-based micronucleus and MultiFlow assays (Fortin et al. 2023). It has been applied for DDI classification or mechanistic investigations in a variety of cell models that have intact p53 function, including HepaRG, MCF-7, and KGN cells, further illustrating its adaptability to alternative biological systems and toxicological questions (Buick et al. 2021; Matteo et al. 2023; Wang et al. 2023; Barutcu 2024). In total, more than 90 compounds have been evaluated using TGx-DDI across six transcriptomic platforms and over four laboratories, consistently producing accurate classifications aligned with known genotoxic mechanisms (Table 1). Collectively, these findings establish TGx-DDI as a reliable and transferable transcriptomic biomarker that enhances the specificity and interpretability of in vitro genotoxicity test batteries.

**Table 1.** Summary of multi-site, multi-cell line, and multi-platform experience with the TGx-DDI biomarker as of December 2024.

Study site	Platform	Cell line	Compounds			Notes	References
			Training set^a^	Test set^b^	Additional		
**TK6 cells**							
Georgetown University	Agilent microarray	TK6*	28^#^	43^#^		Development of the gene classifier in TGx-DDI biomarker.	Li et al. (2015) and Li et al. (2017)
Georgetown University	NanoString	TK6*	28^#^	43	2	Reproduced Agilent microarray results.	Li et al. (2017)
Health Canada	RT-qPCR	TK6*	28^#^	23^#^		Reproduced Agilent microarray results.	Cho et al. (2019)
Health Canada	Agilent microarray	TK6		4	9	High accuracy in prediction of DDI. Anchored against micronucleus frequency.	Buick et al. (2015) and Yauk et al. (2016)
Health Canada	Agilent microarray	TK6			2	**Case study:** Data-poor chemicals requested for analysis by Health Canada’s New Substances Assessment and Control Bureau. Anchored against micronucleus frequency (results showed agreement between the assays that both chemicals are DDI).	Buick et al. (2017)
Procter & Gamble	Affymetrix microarray	TK6	9	1	12	Anchored against micronucleus frequency and the ToxTracker assay. High concordance and demonstration of importance of careful top concentration selection.	Allemang et al. (2021)
Amelia	NanoString PlexSet	TK6	27		17	Concordance between high-throughput Plexset and the standard nCounter codeset assays.	Chen et al. (2022)
Georgetown University	NanoString	TK6			3	**Case study:** Combined with the high-throughput alkaline DNA damage-sensing CometChip assay to demonstrate value of integrated testing to de-risk irrelevant positives.	Buick et al. (2022)
Health Canada	TempO-Seq	TK6					
Health Canada	TempO-Seq	TK6	1	1	11	**Case study:** Data-poor substances for regulatory partners in the Existing Substances Risk Assessment Bureau. TGx-DDI included as part of Health Canada’s GeneTox21 program. TGx-DDI was multiplexed with the MicroFlow and MultiFlow assays. Correct classification of the 2 positive and 2 negative controls using the high-throughput TempO-seq platform on cell lysates. The biomarker yielded concordant calls with the MultiFlow assay and demonstrated lower rates of positives than micronucleus analysis using MicroFlow (as expected).	Fortin et al. (2023)
**HepaRG cells**							
Health Canada	Affymetrix microarray	HepaRG		2	11	Analysis of external dataset (Doktorova et al. 2013) demonstrating 100% accuracy in HepaRG.	Buick et al. (2015)
Health Canada	AmpliSeq	HepaRG	5	1	4	100% accuracy using RNA-seq for classifying 5 genotoxic and 5 nongenotoxic agents, and concordance with the micronucleus assay.	Buick et al. (2020)
Health Canada	TempO-Seq	HepaRG	3	1	8	Confirmed accuracy in performance in HepaRG using TempO-seq and showed added value as an integrated test with the CometChip assay.	Buick et al. (2021)
	TempO-Seq	HepaRG	2	1	7	TGx-DDI classifications compared with GENOMARK biomarker classifications. Both biomarkers had 100% accuracy in classifying compounds. TGx-DDI showed 100% concordance with GENOMARK based on both DDI calls and benchmark concentrations.	Thienpont et al. (2024)

a A set of 28 model agents with well-studied genotoxic or nongenotoxic mechanisms; used to develop the gene classifier panel in the biomarker (Li et al. 2015).

b A set of 45 test compounds selected based on the FDA’s recommendation in the first qualification round; two compounds were included in the training set.

* Cells from a common source batch of cells.

# Chemicals from a common source/lot.

Although the TGx-DDI biomarker has demonstrated utility across a range of applications (Li et al. 2017), the Health and Environmental Sciences Institute (HESI) Emerging Systems Toxicology in the Assessment of Risk (eSTAR) Committee is currently pursuing formal qualification through the U.S. FDA’s Biomarker Qualification Program. As part of this process, the biomarker is being evaluated for a defined regulatory purpose to ensure clarity of use and reproducibility. The following Context of Use (COU) has been defined:

TGx-DDI should be applied following a positive in vitro mammalian chromosomal aberration assay or in vitro micronucleus assay. TGx-DDI results support weight-of-evidence evaluation of whether the positive in vitro finding is relevant to in vivo carcinogenicity (Fig. 1). Although TGx-DDI offers high specificity, its performance relies on functional p53 signaling (Li et al. 2017). TGx-DDI is not applicable to aneugens and may show reduced sensitivity to antimetabolites or compounds that inhibit transcription (Li et al. 2017; Buick et al. 2021). These limitations highlight the importance of applying TGx-DDI as part of an integrated testing strategy for optimal performance and coverage of genotoxic mechanisms.

**Fig. 1.**
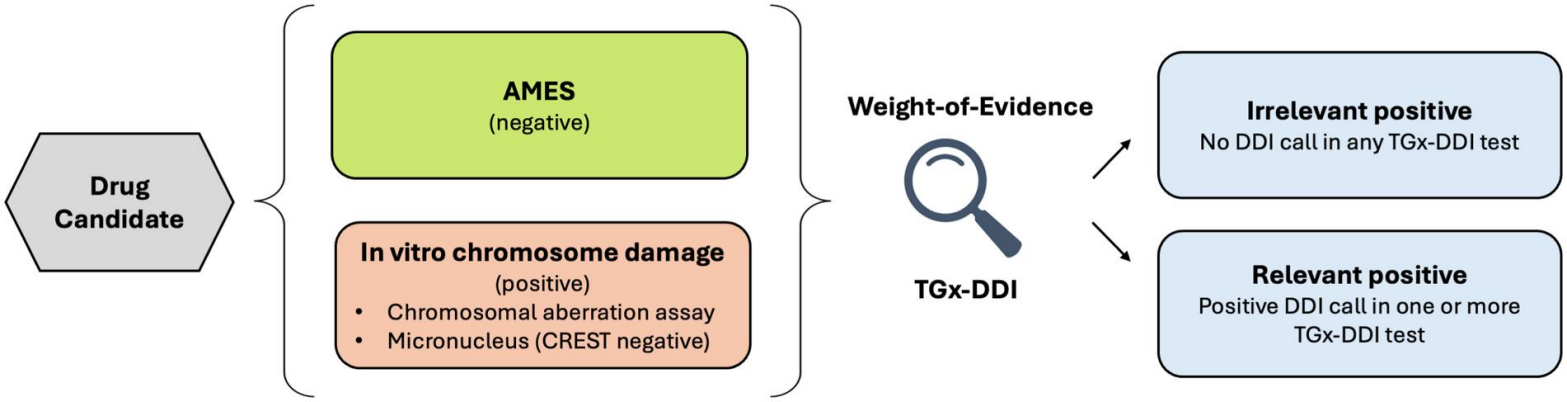
Proposed context of use and workflow for integration of the TGx-DDI transcriptomic biomarker into genotoxicity hazard assessment.

Here, we present the results of a comprehensive ring trial, funded through the FDA Drug Development Tool program (grant no. 1U01FD007473-01). This study evaluated the reproducibility, robustness, and predictive performance of TGx-DDI across four independent laboratories using a harmonized standard operating procedure (SOP) and technology. Results from the ring-trial provide support for TGx-DDI integration into modern genotoxicity testing strategies to improve specificity, reduce unnecessary in vivo testing, and enhance the mechanistic interpretation of genotoxic responses.

## Materials and methods

### Study sites

Four independent laboratories (Georgetown University, The Procter & Gamble Company [P&G], Sanofi-Aventis [Sanofi], and Burleson Research) volunteered to conduct the cell-exposure studies based on demonstrated proficiency in molecular and cellular biology techniques as evidenced by their publications and professional credentials. To preserve confidentiality and avoid singling out any participant, the laboratories are hereafter referred to as Lab 1, Lab 2, Lab 3, and Lab 4, in no particular order. The selected NanoString cores (Children’s National Research Institute and The Wistar Institute Genomics Core) have established proficiency with this platform and are service providers. In addition, each laboratory completed an initial technical assessment by running positive and negative controls (described below) and a reference RNA standard, as per the SOPs provided to each laboratory for TGx-DDI analysis. Accurate classification of the positive and negative controls as DDI or non-DDI, respectively, confirmed the laboratories’ proficiency prior to the full study launch.

The design of this validation ring trial was finalized by HESI Global in consultation with the FDA. All experiments were conducted following standardized SOPs provided to each laboratory, which are included in Supplementary Material S1. These SOPs contain relevant methodological details, including cell culture, treatment, RNA extraction, and data processing workflows.

### Test compounds

Each study site tested 14 compounds (Table 2); three of these compounds required S9 activation. The chemicals were selected in consultation with, and approved by, the FDA to represent a broad range of DDI and non-DDI mechanisms (Li et al. 2017). To ensure consistent assay performance across laboratories, each site included positive (bleomycin and benzo[a]pyrene) and negative (caffeine) control compounds. Two rounds of experiments were conducted in this ring-trial study, referred to as Result 1 and Result 2. In Result 1, all test compounds were run at three preselected concentrations alongside concurrent solvent controls; the highest test concentration was selected based on experience from a previous study (Li et al. 2017). In Result 2, range-finding experiments were independently conducted at each study site to optimize the concentration selection for a subset of compounds, addressing cross-laboratory variability in cytotoxicity measurements observed in Result 1.

**Table 2.** A list of test compounds and concentrations used in the TGx-DDI ring-trial.

Class	Code	Test compound	CAS No.	M.W.	Metabolic activation	Concentration		
						**Low**	**Medium**	**High**
1	A	Etoposide	33419-42-0	588.56	N	22.22 nM	66.67 nM	200 nM
1	B	EMS (Ethyl methanesulfonate)	62-50-0	124.16	N	0.22 mM	0.67 mM	2 mM
1	D	Chlorambucil	305-03-3	304.21	N	0.44 μM	1.33 μM	4 μM
1	E	ENU (N-Nitroso-N-ethylurea)	759-73-9	117.11	N	0.056 mM	0.167 mM	0.5 mM
4	F	D-Mannitol	69-65-8	182.17	N	0.11 mM	0.33 mM	1 mM
4	G	Ampicillin	69-53-4	349.40	N	0.11 mM	0.33 mM	1 mM
4	I	Sunitinib malate^a^	341031-54-7	532.56	N	2.22 μM	6.67 μM	20 μM
5	J	2,4-Dinitrophenol	51-28-5	184.11	N	0.11 mM	0.33 mM	1 mM
5	K	Dexamethasone	50-02-2	392.46	N	0.11 mM	0.33 mM	1 mM
5	N	Staurosporine^a,b^	62996-74-1	466.53	N	3.33 nM	10 nM	30 nM
5	O	Cycloheximide	66-81-9	281.35	N	1.11 μM	3.33 μM	10 μM
N/A	P	Phenobarbital sodium	57-30-7	254.22	Y	0.11 mM	0.33 mM	1 mM
N/A	Q	Cyclophosphamide^b^	6055-19-2	279.1	Y	2.22 μM	6.67 μM	20 μM
N/A	R	2-Aminoanthracene^a^	613-13-8	193.24	Y	1.14 μM	3.47 μM	10.3 μM
Control	CA	Bleomycin (sulfate)	9041-93-4	1,415.6	N	7 or 3.53 μM *		
Control	CB	Caffeine	58-08-2	194.19	N	2 mM		
Control	CC	Benzo[a]pyrene	50-32-8	252.31	Y	39.6 µM		

a,b Test concentrations were redefined and retested in new repeat experiments at ^a^Lab2 or ^b^Lab1.

* Note: The concentration of bleomycin was 7 μM in the first batch of experiments and reduced to 3.5 μM in subsequent experiments.

All the compounds in this study were sourced from a common manufacturer, and aliquoted and blinded at a single facility (Georgetown University) for distribution to the participating laboratories in their stable form (in stock solution). Briefly, each sample was labeled with a code to hide its identity. One member of each laboratory, designated as the “sample preparation technician,” received information to decode the samples. This de-coding was necessary to provide the recipient laboratories with essential safety information. The sample preparation technician prepared the sample dilutions and provided them, with all blinded coding in place, to the investigator, who then applied these samples to the cells and collected the resulting data.

### Transcriptomics platform

All sites participating in the ring trial were required to use the NanoString platform for transcriptomic assessment. Preliminary studies support the high degree of accuracy of the TGx-DDI biomarker in predicting relevant DDI responses on this platform (Li et al. 2017; Chen et al. 2022).

Two study sites, referred to as Lab 1 and Lab 2 throughout the manuscript, conducted cellular exposures and RNA extractions in-house, but then sent the samples to external contracted core facilities (Children’s National Genomics Core and Wistar Institute) for transcriptomic analyses. The outsourcing of high-throughput data generation and analysis is a common practice in industry, government, and academic research and thus is consistent with current practice. The two other sites, Lab 3 and Lab 4, used core services available within their institutes.

### TK6 cell culture and exposure

**Fig. 2.**
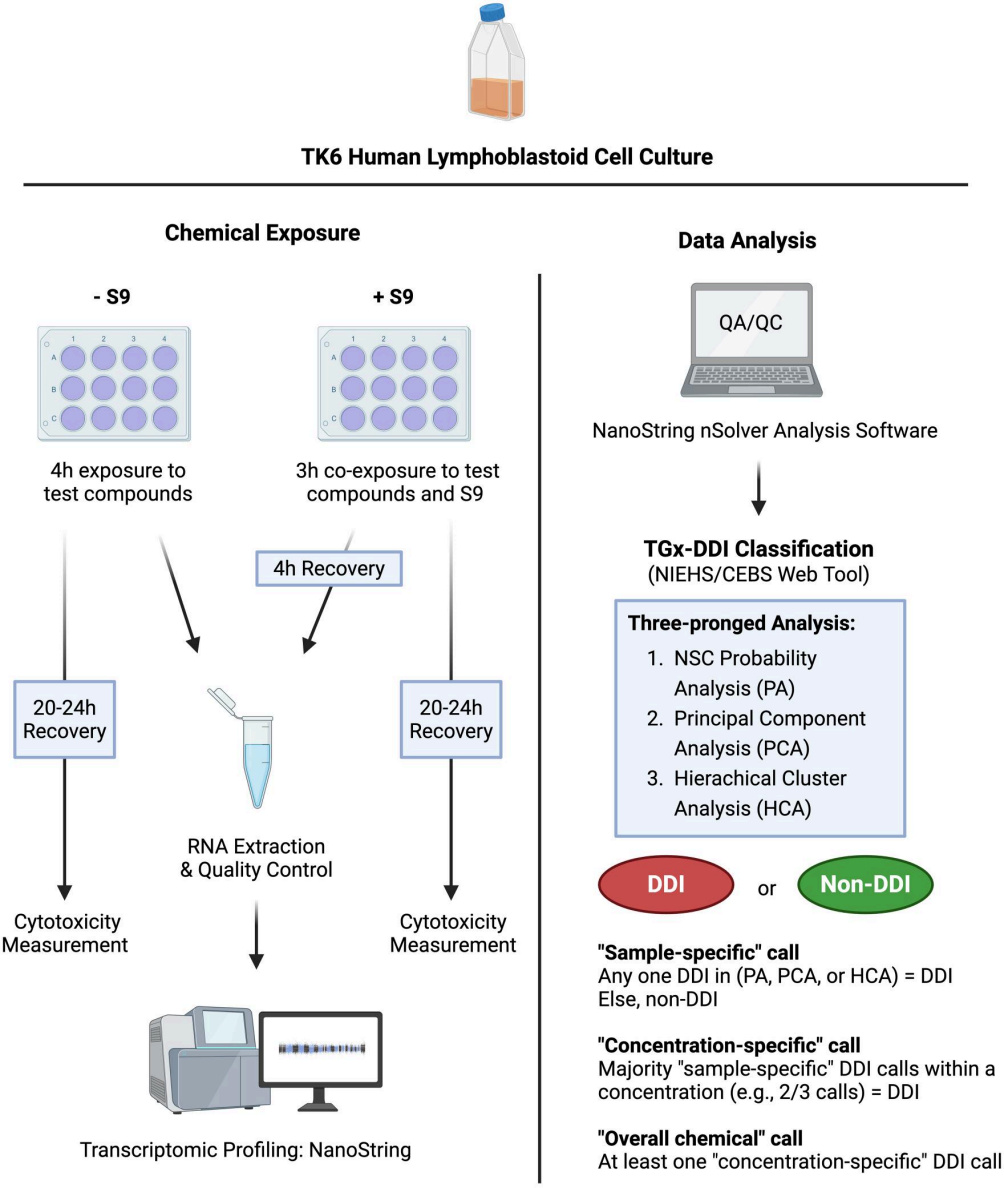
Experimental workflow of the TGx-DDI assay.

To apply the TGx-DDI transcriptomic biomarker, human TK6 cells were purchased from ATCC CRL-8015 and distributed to the participating laboratories to ensure they had the same cell stock and passage number. Cells were exposed to three increasing concentrations of each test compound alongside concurrent solvent controls, using standard culturing conditions (SOPs included as Supplementary Material S1). Three separate wells were treated and analyzed at the same concentration, with a total of three preselected concentrations tested per compound. The workflow of TGx-DDI is described in Fig. 2. Briefly, following a 4-h exposure, cells were pelleted, flash-frozen, and stored at −80°C until RNA extraction. If the test compound required metabolic activation, cells were exposed for 3 h in the presence of rat liver S9 mix and NADPH-regenerating system reagents, followed by an additional 4-h recovery time in fresh media. Additional details, including chemical preparation and S9 use, are available in SOPs. To ensure the cells in each batch were producing a robust and specific response for TGx-DDI transcripts, controls were included in each batch of experiments: 2 mM caffeine as the negative control, bleomycin (either 7 or 3.5 μM) as the positive control without metabolic activation, and 39.6 µM benzo[a]pyrene in the presence of S9 mix as the positive control with metabolic activation. Initially, bleomycin was used at 7 μM; however, this concentration exceeded the cytotoxicity threshold in some laboratories. Therefore, in subsequent experiments, the dose was adjusted to 3.5 μM to remain within the acceptable cytotoxicity range (i.e. 55%±5% cytotoxicity). A portion of the cell suspension was further cultured for an additional 20- to 24-h postexposure to assess relative survival using the MTT [3-(4,5-dimethylthiazol-2-yl)-2,5-diphenyltetrazolium bromide] cell proliferation assay. In brief, 10 μl of MTT solution (5 mg/ml in Assay Buffer) was added to each well and incubated at 37°C for 3 h. The resulting formazan crystals were dissolved in 100 μl of crystal dissolving solution, incubated for 4 to 18 h, and absorbance was measured at 570 nm using a microplate reader.

### RNA extraction and quality assessment

After exposures, RNA was extracted from cells following the SOP provided (Supplementary Material S1). The integrity of RNA samples was assessed immediately prior to transcriptional profiling and TGx-DDI analysis. Specifically, RNA integrity numbers (RINs) were produced using Agilent RNA 6000 Nano Bioanalyzer kits and Agilent 2100 Bioanalyzers or Invitrogen Qubit Fluorometers. Samples with RINs >7 were considered amenable to TGx-DDI analyses, according to the field conventions.

### NanoString nCounter gene expression and data analysis

NanoString nCounter Analysis Systems were used for RNA analysis following the SOP and kit- and platform-specific manufacturer instructions. Note that Georgetown employed a multiplexed high-throughput assay format within the NanoString platform (Nanostring PlexSet). Data analysis was done independently at each laboratory, following the corresponding SOPs and a statistical analysis plan (Supplementary Material S1). The NanoString nSolver Analysis Software was used for quality assurance/quality control (QA/QC) of the gene expression data. A comprehensive set of QA/QC parameters was established in the analysis pipeline, with details provided in the ring trial SOPs (Supplementary Material S1). Samples with QA/QC flags were re-examined and removed if necessary, following the instructions on flag examination specified in SOPs. The transcript levels in exposed cells were normalized to those in their respective solvent controls. To facilitate data sharing for omics experiments, details of data processing approaches and study designs are organized and provided in the OECD Omics Reporting Framework (Supplementary Material S2).

### TGx-DDI biomarker classification (making a DDI or non-DDI call)

TGx-DDI classification was completed through an open-access analysis data tool available via the National Institute of Environmental Health Sciences (NIEHS) Chemical Effects in Biology Systems (CEBS) database (https://cebs.niehs.nih.gov/tgxddi/tool). Data analysis was conducted independently at each of the participating laboratories using a shared, predefined statistical analysis plan and standardized SOPs to minimize subjective interpretation (Supplementary Material S1). Briefly, a three-pronged approach, comprising Nearest Shrunken Centroid Probability Analysis (PA), Principal Component Analysis (PCA), and Hierarchical Cluster Analysis (HCA), was applied to each test sample to derive a DDI or non-DDI call. The three statistical methods ensure comprehensive and robust classification that prioritizes sensitivity to minimize false negatives. Concentrations showing overt cytotoxicity (i.e. viability <40% based on the MTT assay at 24 h) were excluded from the analysis. In contrast, the top concentration had to induce 55% ± 5% cytotoxicity (i.e. 40% to 50% viability, as recommended in OECD TG 487) to be considered a valid test for making a negative TGx-DDI call, unless it reached or exceeded 1 mM as specified in ICH S2(R1) guideline (Table 2).

The overall DDI classification was derived through a step-by-step process.

1. “Sample-specific” call: At the sample level, each experimental sample received a classification call. If a positive DDI call was made in any of the analysis methods (PA, PCA, HCA) in the three-pronged approach, the sample was classified as DDI. If all the methods generated a non-DDI call, the compound was classified as non-DDI. Compounds may produce an “inconclusive” call under specific conditions: When the PA probability is below 0.9 for either DDI or non-DDI classification, when the compound falls on the PC1 axis, or if the compound does not fall distinctly on either the DDI or non-DDI branch of the dendrogram. For samples without a definitive DDI classification, a majority rule was used to classify them as non-DDI or inconclusive.
2. “Concentration-specific” call: A grouped analysis was then conducted to classify each concentration of a compound. As with the classification of individual samples, if a majority of samples (e.g. two out of three) within a concentration produced a DDI call, the concentration was called DDI in the “grouped analysis.” Similarly, samples were classified by the majority rule as either non-DDI or inconclusive.
3. “Overall chemical” call: Finally, a compound was classified as DDI if a DDI call was made at any concentration (else it was considered non-DDI or inconclusive).

### Within-laboratory reproducibility

Within-laboratory (technical) reproducibility was assessed by comparing the TGx-DDI classification calls from the three technical replicates for each treatment condition. Only valid test results were included, with control treatments (bleomycin, benzo[a]pyrene, caffeine) excluded from the analysis. For each laboratory, the proportion of compounds with identical calls across all three replicates was calculated.

### Cross-site concordance analysis

We evaluated the level of concordance in TGx-DDI calls made for the same compound between study sites. This analysis was done by Dr Andrew Williams (Health Canada), who was independent of the participating laboratories. The overall positive and negative DDI calls were compiled into 2 × 2 contingency tables for each pair of the four study sites (Supplementary Material S3, concordance_analysis). Three statistical metrics, the Cohen’s Kappa (Cohen 1960), the prevalence and bias-adjusted Kappa (PABAK) (Byrt et al. 1993), and AC1 (Gwet 2010), were calculated for each table as an integrated approach to interpret the level of agreements (Kirkland et al. 2019). The results were interpreted as follows: Poor (<0.2), fair (0.21 to 0.4), moderate (0.41 to 0.6), good (0.61 to 0.8), very good (0.81 to 1) (Landis and Koch 1977).

### Data processing and correlation analysis of reference RNA

A common reference RNA standard (Human Universal Reference RNA, uhrRNA Agilent Cat No. 740000, Santa Clara, CA) was included in each batch of experiments to facilitate a robust comparison of the technical performance across study sites. Raw count data from each laboratory were imported into R and the datasets were merged by gene ID to create a unified dataset. To account for differences in count depth across laboratories, counts were scaled relative to the total counts per laboratory and log_2_ transformed to stabilize variance and improve comparability across datasets. All analyses were conducted in R version 4.3.2 on a Windows x86_64 platform. Pairwise Pearson and Spearman correlation coefficients were computed using the cor() function. Pearson correlation was used to assess linear relationships, whereas Spearman correlation was used to evaluate rank-based consistency between laboratories.

## Results

All four participating laboratories successfully implemented the ring-trial SOPs and correctly classified the positive (bleomycin and benzo[a]pyrene) and negative (caffeine) controls, to demonstrate assay proficiency (Table 3).

**Table 3.** Summary of TGx-DDI ring-trial calls and test accuracy for the 14 test compounds and 3 positive or negative controls.

Code	Compound	Expected TGx-DDI call	Result 1			Result 2		
			Valid tests*	Correct calls	Test accuracy	Valid tests*	Correct calls	Test accuracy
A	Etoposide	+	3	3	100%	3	3	100%
B	Ethyl methanesulfonate	+	4	4	100%	4	4	100%
D	Chlorambucil	+	2	2	100%	2	2	100%
E	ENU (N-Nitroso-N-ethylurea)	+	4	4	100%	4	4	100%
Q	Cyclophosphamide	+	3	3	100%	**4**	**4**	**100%**
R	2-Aminoanthracene	+	1	1	100%	**2**	**2**	**100%**
F	D-Mannitol	−	4	4	100%	4	4	100%
G	Ampicillin	−	4	4	100%	4	4	100%
I	Sunitinib malate	−	2	0	0%	**3**	**1**	**33%**
J	2,4-Dinitrophenol	−	4	4	100%	4	4	100%
K	Dexamethasone	−	4	4	100%	4	4	100%
N	Staurosporine	−	0	0	n/a*	**2**	**2**	**100%**
O	Cycloheximide	−	3	1	33%	3	1	33%
P	Phenobarbital sodium	−	4	4	100%	4	4	100%
CB	Caffeine	−	4	4	100%	4	4	100%
CA	Bleomycin (sulfate)	+	4	4	100%	4	4	100%
CC	Benzo[a]pyrene	+	4	4	100%	4	4	100%

* n/a: Not available. Result 1 summarizes the first round of TGx-DDI analyses; Result 2 includes new repeat experiments for compounds Q, R, I, and N. Differences between Results 1 and 2 are indicated in underlined and bold text.

* Valid tests: Top concentration causes a 50% to 60% reduction in cell viability and/or achieve solubility limits or exceed 1 mM.

After confirming laboratory proficiency, the ring-trial study was conducted in two phases, referred to as “Result 1” and “Result 2” in the tables below. All data and analysis outputs are available via the NIEHS CEBS data repository  (NIEHS Chemical Effects in Biological Systems (CEBS) Database 2025).

### Sample inclusion and exclusion

All samples passed RNA quality control (RIN >7) and underwent rigorous QA/QC evaluation prior to inclusion in the analysis, as detailed in Appendix S1 (Supplementary Material S1). Across the four laboratories, a total of six replicates were excluded due to QC flags related to mRNA content normalization (Supplementary Material S4). In addition, 42 sample-specific calls (triplicates of 14 experiments) were excluded for not meeting the predefined cytotoxicity thresholds: 27 non-DDI calls were removed because the top concentration failed to induce the required 50% to 60% cell death to support a negative TGx-DDI call, and 15 replicates were excluded due to overt cytotoxicity (>60%). After these exclusions, at least two replicates remained for each treatment group.

### Initial testing phase (result 1)

Our initial testing phase used concentrations for the 14 test compounds selected from the literature or based on in-house experience with TK6 cell exposures at Georgetown University. Across laboratories, 56 independent experiments (each comprising three technical replicates) were performed in TK6 cells, of which 42 were considered “valid” because they reached the target cytotoxicity level (50% to 60% cell death at the top concentration) or were at, or above, the 1 mM concentration limit. The overall TGx-DDI classification calls for the 14 test compounds (six DDI agents and eight non-DDI agents) from this Phase are summarized and compared with the expected calls (Table 3, Result 1).

The participating laboratories generated 17 valid tests for the six DDI agents. TGx-DDI demonstrated outstanding performance—correctly classifying all of them, achieving 100% sensitivity (95% confidence interval, CI: 80% to 100%). For the 25 valid tests on non-DDI agents, TGx-DDI generated correct calls in 21 of them, achieving 84% specificity (CI: 64% to 95%). Notably, five non-DDI compounds (D-mannitol, ampicillin, 2,4-dinitrophenol, dexamethasone, and phenobarbital sodium) were classified with 100% individual accuracy. However, the classification accuracy was lower for sunitinib malate (0%) and cycloheximide (33%). For sunitinib malate, only two laboratories met the required cytotoxicity, but neither produced a correct call. Three of the laboratories generated valid tests for cycloheximide, but only one correctly classified it as non-DDI. No classification was made for staurosporine (indicated as “n/a”), as none of the tests induced sufficient cytotoxicity at the top test concentration. A detailed summary of site-specific results is provided in Supplementary Material S3 (compiled_result 1). Overall, TGx-DDI achieved 90% classification accuracy (CI: 77% to 97%) across all valid tests in the initial testing phase.

When excluding the controls and considering only valid tests, all laboratories exhibited complete concordance (100%) among technical replicates except Lab 4, which had one discordant result (for chlorambucil) out of 12 compounds evaluated, yielding a within-laboratory concordance rate of 91.7% (11/12). These findings demonstrate high technical reproducibility of the TGx-DDI assay across multiple laboratories.

### Optimization phase (result 2)

During the initial testing phase using predetermined test concentrations, ∼20% of tests failed to meet the cytotoxicity thresholds and were excluded from TGx-DDI analysis. To increase the number of valid tests, two participating laboratories (Lab 1 and Lab 2) conducted additional range-finding experiments for specific compounds and repeated the tests under optimized conditions to meet the cytotoxicity threshold. Lab 1 redefined test concentrations for cyclophosphamide and staurosporine, whereas Lab 2 optimized the concentrations and retested 2-aminoanthracene, sunitinib malate, and staurosporine. Researchers were not blinded in this testing phase. The positive and negative controls were tested alongside the test compounds to ensure test reliability.

The optimized test concentrations were confirmed to meet the cytotoxicity thresholds, and all repeat experiments generated correct TGx-DDI classifications. Notably, the individual classification accuracy for staurosporine improved from 0% (no valid tests) to 100% (2/2 correct calls) (Table 3). A detailed summary of TGx-DDI calls from new experiments is provided in Supplementary Material S3 (compiled_result 2).

Overall, result 2 experiments increased the number of valid tests and contributed to improved performance of TGx-DDI. When these new data were included in the full analysis (47 valid tests in total), the assay achieved 100% sensitivity (CI: 82% to 100%), 86% specificity (CI: 67% to 96%), and 91% overall accuracy (CI: 80% to 98%) (Table 4).

**Table 4.** Summary statistics of test sensitivity, specificity, and accuracy.

Summary statistics (result 1)				Summary statistics (result 2)			
		Truth standard				Truth standard	
**Pooled**	** **	DDI	non-DDI	**Pooled**		DDI	non-DDI
TGx-DDI	DDI	17	4	TGx-DDI	DDI	19	4
	non-DDI	0	21		non-DDI	0	24

	Estimate	Lower limit	Upper limit		Estimate	Lower limit	Upper limit

Sensitivity	100%	80%	100%	Sensitivity	100%	82%	100%
Specificity	84%	64%	95%	Specificity	86%	67%	96%
Accuracy	90%	77%	97%	Accuracy	91%	80%	98%

### Reference RNA correlation analysis

Gene expression counts from a common reference RNA sample were analyzed to assess technical consistency across the four study sites. The highest Pearson correlation was observed between Lab 1 and Lab 2 (0.999), indicating near-identical measurements (Table 5). Although Labs 3 and 4 showed slightly greater variability, all interlaboratory Pearson coefficients remained high (>0.84), reflecting strong reproducibility. Spearman correlations followed similar patterns and were slightly higher in most cases, confirming consistent rank order across datasets. These findings demonstrate that the NanoString nCounter platform generated highly reproducible transcriptomic data across all participating laboratories.

**Table 5.** Pairwise correlation analysis of reference RNA.

**Pearson correlation coefficients**					**Spearman correlation coefficients**				
	Lab1	Lab2	Lab3	Lab4		Lab1	Lab2	Lab3	Lab4
**Lab1**	1.000	0.999	0.996	0.849	**Lab1**	1.000	0.998	0.993	0.862
**Lab2**	0.999	1.000	0.995	0.841	**Lab2**	0.998	1.000	0.992	0.857
**Lab3**	0.996	0.995	1.000	0.848	**Lab3**	0.993	0.992	1.000	0.877
**Lab4**	0.849	0.841	0.848	1.000	**Lab4**	0.862	0.857	0.877	1.000

### Cross-site concordance analysis

**Fig. 3.**
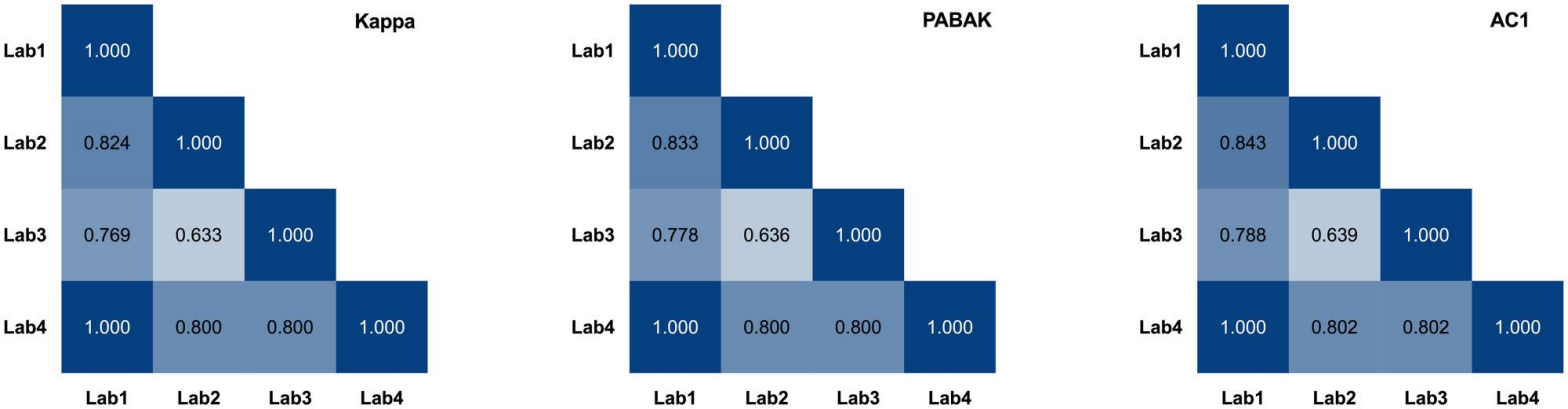
Concordance analysis of TGx-DDI calls across study sites in the ring-trial. Agreement between pairs of laboratories was evaluated using Cohen’s kappa, the prevalence and bias-adjusted Kappa (PABAK), and Gwet’s AC1 statistics. Each cell represents the agreement score for TGx-DDI classification between two study sites, with higher value and greater color intensity indicating stronger interlaboratory concordance. Levels of agreement were interpreted as follow: Poor (<0.2), fair (0.21 to 0.4), moderate (0.41 to 0.6), good (0.61 to 0.8), very good (0.81 to 1). Laboratories are presented in the same order (Labs 1 to 4) across all three matrices.

To assess the reproducibility of TGx-DDI classifications across study sites, concordance analysis was performed using three metrics: Kappa, AC1, and PABAK, as previously described in Kirkland et al. (2019). The results indicate good (coefficients between 0.61 and 0.8) to very good (>0.8) agreement in TGx-DDI calls produced across laboratories. Notably, Lab 1 and Lab 4 achieved perfect concordance in all the valid tests (Kappa, AC1, and PABAK = 1) (Fig. 3 and Supplementary Material S3).

## Discussion

The successful completion of the ring trial marks a critical milestone in the qualification of the TGx-DDI biomarker. With optimized concentration selection, TGx-DDI correctly classified 6/6 DDI compounds and 6/8 non-DDI compounds, to achieve 100% sensitivity, 86% specificity, and 91% overall accuracy in this ring-trial. These results represent a substantial improvement over traditional chromosomal damage assays such as the in vitro micronucleus test, which has reported specificities of 30% to 54% (Kirkland et al. 2005), and 65% (Avlasevich et al. 2021). Moreover, the highly concordant results produced across participating study sites (agreement coefficients ranging from 0.63 to 1) support the feasibility of applying TGx-DDI in different laboratory settings. This ring trial represents a pivotal step toward regulatory acceptance, confirming that TGx-DDI meets essential criteria for qualification and future integration into safety assessment frameworks.

The experiences gained from this ring trial also enabled the finalization of a test guidance for the use of TGx-DDI as a promising follow-up tool for in vitro genotoxicity tests. A final qualification report for the biomarker and the associated protocols have now been submitted to the U.S. FDA Biomarker Qualification Program (https://www.fda.gov/regulatory-information/search-fda-guidance-documents/qualification-process-drug-development-tools-guidance-industry-and-fda-staff). If accepted, this will mark the first transcriptomic biomarker qualified by FDA CDER.

### Generic test guidance

To support future use of TGx-DDI, we developed generic test guidance based on SOPs used in this ring trial and practical lessons learned. Key components include: (i) range-finding experiments for optimal concentration selection, (ii) comprehensive proficiency testing, and (iii) flexibility in cell line and platform selection.

#### Range-finding experiments

Selecting appropriate test concentrations is critical for valid TGx-DDI test outcomes, as strict cytotoxicity thresholds must be met. The initial testing phase with preselected concentrations resulted in suboptimal cytotoxicity outcomes and led to the exclusion of about 20% of tests. Additional range-finding experiments remedied these issues and improved specificity and test accuracy. The test guidance, therefore, specifies the need to conduct range-finding experiments for new compound testing.

#### Proficiency testing

TGx-DDI has demonstrated robust responses across various transcriptomic platforms, including microarrays, NanoString, RT-qPCR, RNA-Seq, and TempO-Seq (summarized in Table 1). Given the evolving landscape of omics technologies, it is neither necessary nor appropriate to restrict TGx-DDI implementation to a single platform. Instead, its flexibility across platforms reflects one of its key strengths and supports broader applicability in both research and regulatory settings. However, when applying TGx-DDI on a new or modified platform, we recommend that laboratories first demonstrate performance equivalency through proficiency testing. To do so, laboratories should conduct two sets of experiments under the experimental conditions they intend to use. First, the laboratory should verify their ability to correctly classify the positive and negative control compounds (caffeine, bleomycin, benzo[a]pyrene) following TGx-DDI SOPs. Second, they should test an additional set of five compounds including two non-DDI agents, two DDI agents that do not require metabolic activation, and one DDI agent that requires metabolic activation. We recommend testing five compounds that show robust and reproducible results across cell lines and platforms: D-mannitol, ampicillin, etoposide, ethyl methanesulfonate, and cyclophosphamide. Detailed procedures are described in the generic test guidance in Supplementary Material S5. To support accessibility and adoption of TGx-DDI, we collaborated with the nonprofit organization U.S. Pharmacopeia (https://www.usp.org/) to establish a TGx-DDI proficiency testing library, which will provide quality assured and validated compounds for laboratories to establish and extend the application of this biomarker to new platforms. Overall, our proposed proficiency testing approach enables consistent application of TGx-DDI across diverse technologies while maintaining the performance standards required for regulatory confidence.

#### Performance considerations and risk mitigation for TGx-DDI

Despite serving as a promising follow-up test that addresses the low specificity of traditional genotoxicity assays, TGx-DDI has a few limitations. This section outlines key considerations for its use in novel compound testing, with a focus on potential challenges and strategies to mitigate associated risks.

Adherence to TGx-DDI SOPs and test guidance is essential to minimize type I and type II errors. We provide rigorous laboratory SOPs that support labs to perform the assay using GLP (good laboratory practice) like standards (Kauffmann et al. 2017). Since the TGx-DDI classifier gene panel is enriched for transcripts regulated by p53, to apply TGx-DDI in cell lines other than human TK6 cells and HepaRG hepatocytes, users must verify the integrity of the p53 pathway and the metabolic competence of the cells (Corton et al. 2019). When necessary, the use of metabolic activation systems, such as S9, is required. We also recommend completing the proficiency test described above to confirm acceptable performance in new model systems.

Given that TGx-DDI relies on the upregulation of stress response genes, obtaining a robust transcriptomic signal is particularly important for reliable data interpretation. Compounds that inhibit transcription, such as certain nucleoside reverse transcriptase inhibitors like Zidovudine, can yield false negatives in TGx-DDI analysis (Buick et al. 2021). To mitigate this risk, we recommend conducting QA/QC analysis as per the bioinformatics pipeline and verifying the expression of key stress response genes (e.g. *ATF3*, *CDKN1A*, and *GADD45A* in TK6 cells; described in Li et al. 2015, 2017) to ensure that the cellular responses are measurable and intact under the test conditions. TGx-DDI also shows reduced sensitivity to antimetabolites; the genotoxic effects of antimetabolites may manifest at later time points, thus additional work would be needed to develop a study design more suited to this mechanism of action (Li et al. 2017). Additionally, nongenotoxic compounds that target the p53 pathway or related signaling pathways (e.g. p53 activators, kinase inhibitors) may produce false-positive calls in TGx-DDI analysis (Corton et al. 2019). In our ring trial, while 10 compounds were classified with 100% accuracy, two non-DDI agents (sunitinib malate and cycloheximide) generated false-positive results and reduced overall specificity. These effects likely reflect nongenotoxic modulation of gene expression, such as altering cell signaling and metabolism as reported in other cells (Shukla et al. 2009; Oksvold et al. 2012; Cho et al. 2019). Combining TGx-DDI with additional endpoints may help mitigate these limitations (discussed in the following section).

Of note, in the present study, each concentration was run in triplicates (3 separate wells treated with the test compound, or control, within the same experiment) to assess the assay pipeline under tightly controlled conditions. Although independent experiments would be valuable for capturing additional sources of variability for new compound analysis, the current design is consistent with standard practice in genotoxicity assays and OECD guidance. Future studies should explore integration of independent repeat experiments to further strengthen robustness. Importantly, our classification paradigm is intentionally designed to be conservative—classifying a compound as DDI-positive if any of the three methods (PA, PCA, HCA) yields a positive result—to minimize the risk of false negatives. Although this may increase the chance of false positives, such calls do not introduce additional human safety concerns within weight-of-evidence frameworks, where other supporting data are considered.

### Compatibility with other genotoxicity tests for weight-of-evidence evaluation

Integrating TGx-DDI with other genotoxicity tests improves predictive performance and provides additional mechanistic insights. The in vitro micronucleus assays (e.g. OECD TG 487, ICH S2 (R1)) are widely used to assess clastogenicity and aneugenicity but suffer from low specificity in some cases (Kirkland et al. 2005; Avlasevich et al. 2021). Exposure to TGx-DDI-positive chemicals results in concomitant increases in micronucleus frequency in TK6 and HepaRG cells (Buick et al. 2015, 2017, 2020; Yauk et al. 2016; Allemang et al. 2021; Fortin et al. 2023). The combined use of TGx-DDI and the micronucleus assay enhances classification accuracy while retaining the ability to detect aneugens (Buick et al. 2020; Allemang et al. 2021). Similar benefits have been observed when TGx-DDI is used with CometChip, which detects DNA strand breaks (Buick et al. 2021, 2022), and MultiFlow, a high-content assay that characterizes genotoxic modes of action (Fortin et al. 2023). Moreover, a recent study in HepaRG cells showed that TGx-DDI and another transcriptomic biomarker GENOMARK (comprising 84 gene with minimal overlap) produced identical classifications of genotoxicants (Thienpont et al. 2024). We recommend incorporating TGx-DDI within a weight-of-evidence framework alongside in silico *or* in vitro methods, and relevant pharmacokinetic and mechanistic data to support informed decision-making in genotoxicity assessments.

### Application beyond the defined context of use

In addition to its original proposed context of use as a follow-up assay for in vitro genotoxicity testing, TGx-DDI shows potential as a valuable tool in chemical screening and drug development. The method is easy to implement, works reliably across laboratories and platforms, and includes a simple, objective data analysis pipeline. Its high sensitivity and specificity in distinguishing irrelevant in vitro positive results can help reduce unnecessary follow-up testing, thereby supporting more safe and efficient chemical screening and drug development. Although the current qualification supports TGx-DDI as a follow-up assay, future applications may explore its use earlier in the genotoxicity testing workflow, particularly as toxicogenomic approaches continue to evolve as first-tier tools in chemical hazard assessment.

TGx-DDI may be used to identify drugs that activate the p53 pathway, a key player in DNA damage response and a major target in cancer therapy (Brown et al. 2009; Corton et al. 2019). Using this biomarker, Corton et al. (2019) identified approximately 100 p53 activators from a screen of ∼1950 chemicals across 9800 human cell lines. By providing mechanistic information relating to the induction of DNA damage, TGx-DDI helps evaluate the genotoxic potential of the drug candidates and reduces uncertainty in regulatory decision-making during drug development.

When applicable, benchmark concentration analysis of TGx-DDI genes adds quantitative insight to the standard binary DDI or non-DDI classification. These data can be used to compare chemical potencies and, when paired with in vitro to in vivo extrapolation modeling, help connect research findings to real-life human health risk assessments (Beal et al. 2023).

## Conclusion

The TGx-DDI biomarker is a reliable, reproducible, and mechanistically informative tool for identifying DNA damage-inducing agents. In this successful multi-site ring trial, it demonstrated consistent and robust performance across laboratories. The assay is supported by well-developed wet and dry lab protocols, ensuring ease of transfer and reproducibility in diverse testing environments. Incorporating TGx-DDI into regulatory workflows could help distinguish irrelevant positive results and improve the specificity of genotoxicity assessments. Its flexibility and potential for broader applications beyond its original context of use highlights its value as a valuable tool for safer and more efficient chemical and drug evaluation.

## Supplementary Material

kfaf138_Supplementary_Data
