# Supplementary material for: TGx-DDI (toxicogenomic DNA damage-inducing) biomarker validation: multi-site ring trial supporting regulatory use
Source: Toxicol Sci. 2025 Oct 1;208(2):233–43. doi: 10.1093/toxsci/kfaf138 (PMC12646585; doi:10.1093/toxsci/kfaf138)
Supplement: kfaf138_Supplementary_Data [file kfaf138_supplementary_data.zip › kfaf138_Supplementary_Data/toxsci-25-0384-File005.pdf]

TGx-DDI (Toxicogenomic DNA Damage-Inducing) Biomarker Validation: Multi-Site Ring Trial Supporting Regulatory Use

Authors: Xiaotong Wang, Christine E. Crute, Ashley Allemang, Jiri Aubrecht, Florence Burleson, Yasmin Dietz-Baum, Lena Dorsheimer, Albert Fornace, Roland Froetschl, Ulrike Hemmann, Constance Mitchell, Stefan Pfuhler, Andrew Williams, Lorreta Yun-Tien Lin, Syril Pettit, Carole Yauk, Henghong Li

Supplemental Material 1

TGx-DDI Transcriptomic Biomarker:
Ring Trial SOPs for Participating Laboratories

Table of Contents

SECTION 1. VALIDATION STUDY DESIGN **3**

1A. LIST OF MATERIALS PROVIDED 4

SECTION 2. TEST COMPOUND PREPARATION **5**

SECTION 3. TK6 CELL CULTURE AND TREATMENT SOP **9**

SECTION 4. NANOSTRING SOPS **15**

4A. RNA EXTRACTION FROM CELL CULTURE 15

4B. RNA QUALITY CONTROL 18

4C. NCOUNTER GENE EXPRESSION ANALYSIS 22

SECTION 5. DATA ANALYSIS SOP (USING CEBS TGX-DDI BIOMARKER TOOL) **32**

SECTION 6. DATA INTERPRETATION SOP (MAKING A DDI OR NON-DDI CALL) **35**

SECTION 7. STATISTICAL ANALYSIS **36**

APPENDIX I. QA/QC INSTRUCTIONS **40**

APPENDIX II. TGX-DDI DATA ANALYSIS PROTOCOL USING NSOLVER AND NIEHS/CEBS TGX-DDI WEB TOOL **49**

Section 1. Validation Study Design

Objectives

To assess the cross-laboratory reproducibility of TGx-DDI classification calls involving one platform (NanoString), four sites, and 14 chemicals (at three concentrations alongside solvent controls) with positive and negative controls.

Experimental Workflow Overview

- Each site received the test compound and compounds of positive and negative controls in solution form in an Eppendorf tube labeled with a code that masks the compound identity. The detailed dilution procedure for three concentrations including solvent information was provided.

- TK6 cell culture and exposure were carried out following the attached SOP (Section 3). Samples were prepared in triplicate for each condition, including the vehicle control. Parallel cytotoxicity measurement (using the MTT assay) was carried out.

- RNA samples that passed quality assessment, along with a universal human reference RNA sample were subjected to NanoString assay (Section 4).

- Data were imported to an online tool (https://cebs.niehs.nih.gov/tgxddi/tool) and a three-pronged approach was used to make a TGx-DDI classification call. Detailed procedures for data analysis and interpretation were described in Section 5 and appendices I&II.

Data Presentation and Reporting

In response to the FDA request, each site provided their raw data, processed data, analytical output of the CEBS TGx-DDI biomarker tool, their classification call for each of the models (probability analysis, principal component analysis, hierarchical cluster analysis) for each concentration, and their overall call back to the study coordinators, Dr. Syril Pettit (spettit@hesiglobal.org) and Dr. Chrissy Crute (ccrute@hesiglobal.org). The DDI and non-DDI calls in the ring-trial study were collated in summary tables.

Cross-Site Data Analysis

Results (DDI or non-DDI) calls for all sites and all compounds were compiled into a single data table for comparison of results (Annex 3). A concordance analysis was done based on the classification of each chemical.

1a. List of Materials Provided

Below we list the materials that we provided for each section of this protocol:

- Test and control compounds were provided in reconstituted solution form except compound B which was originally in liquid form
- One tube of 1M NH4OH (one of the solvents)
- Three vials of TK6 cells (ATCC® CRL-8015™), with around 4 x 10^6 cells per cryovial
- S9 mix and NADPH regenerating system: Moltox LS9 SD Phenobarbital/Benzoflavone (Male rat liver in 0.15M KCl., 2.1ml, product no. 11-05L.2); Regensys™ NADPH Regenerating System Reagents (product no. 60-200.15 & 60-201.15L)
- Qiagen RNeasy Mini Kit (for 220 samples, Catalog no. 74106)
- QIAShredder (for 220 samples, Catalog no. 79656)
- NanoString probeset and cartridges (for all the NanoString assays)
- Universal Human Reference RNA (uhrRNA Agilent Cat# 740000)

Supplies that were not provided:

- Solvents to reconstitute test compounds except 1M NH4OH.
- Pipets, 15 ml centrifuge tubes, and Eppendorf tubes.
- Cell culture T75 flasks (Fisher Scientific Catalog no. 07-202-003), 12-well plates (Fisher's Scientific Cat. # 07-200-82), 96-well plates (Fisher's Scientific Catalog no. 07-200-87).
- Cell culture medium: RPMI 1640 (Gibco Thermo Fisher Catalog no. 11875093), FBS (Gibco Thermo Fisher Catalog no. 16000044), and Gentamycin (Gibco Thermo Fisher catalog no. 15750078)
- MTT Cell Viability Kit x2, 480 wells each kit (Cayman Chemical Cat. # 10009365)
- Ethanol (96-100%)
- β-mercaptoethanol (β-ME)

Section 2. Test Compound Preparation

SOP for Chemical Distribution and Blinding for Participating Laboratories

All compounds were blinded prior to testing. The study design was based on the standard operating procedure (SOP) established for the validation ring trial by HESI in consultation with the FDA (funded by FDA 5U01FD006676-02). Briefly, each sample was labeled with a code to hide its identity. One member of each laboratory, designated as the 'sample preparation technician', received information to decode the samples. This de-coding was necessary to provide the recipient laboratories with essential safety information. The sample preparation technician prepared the sample dilutions and provided them — with all blinded coding in place — to the investigator who then applied these samples to the cells and collected the resulting data.

Table 1 lists all compounds used in this trial, which were distributed to each lab from Georgetown University. The compounds were labeled with the blinded code. The codes displayed in this table were randomized for the experiments.

Table 1. The compound list including the blind code and Stock solution concentrations.

Class	Blinded Code	Test Compound	CAS No.	Metabolic Activation	Concentration			MW	Solvent	Stock solution concentration (mg/ml)
					High	Medium	Low			
1	A	[redacted]	[redacted]	N	200 nM	66.67 nM	22.22 nM	588.56	DMSO	4.81
1	B	[redacted]	[redacted]	N	2 mM	0.67 mM	0.22 mM	124.16	N/A	1204.82
1	D	[redacted]	[redacted]	N	4 μM	1.33 μM	0.44 μM	304.21	EtOH	48.08
1	E	[redacted]	[redacted]	N	0.5 mM	0.167 mM	0.056 mM	117.11	DMSO	58.82
4	F	[redacted]	[redacted]	N	1 mM	0.33 mM	0.11 mM	182.17	H2O	90.91
4	G	[redacted]	[redacted]	N	1 mM	0.33 mM	0.11 mM	349.40	1M NH4OH	140.35
4	I	[redacted]	[redacted]	N	20 μM	6.67 μM	2.22 μM	532.56	DMSO	2.14
5	J	[redacted]	[redacted]	N	1 mM	0.33 mM	0.11 mM	184.11	MeOH	36.76
5	K	[redacted]	[redacted]	N	1 mM	0.33 mM	0.11 mM	392.46	MeOH	25.00
5	N	[redacted]	[redacted]	N	30 nM	10 nM	3 nM	466.53	MeOH	0.14
5	O	[redacted]	[redacted]	N	10 μM	3.33 μM	1.11 μM	281.35	H2O	14.01
N/A	P	[redacted]	[redacted]	Y	1 mM	0.33 mM	0.11 mM	254.22	H2O	500.00
N/A	Q	[redacted]	[redacted]	Y	20 μM	6.67 μM	2.22 μM	279.10	EtOH	28.01
N/A	R	[redacted]	[redacted]	Y	2 μg/ml	0.67 μg/ml	0.22 μg/ml	193.24	DMSO	10.00
Control	CA*	[redacted]	[redacted]	N		5 μg/ml*		1415.60	H2O	2.00
Control	CB	[redacted]	[redacted]	N		2 mM		194.19	H2O	15.00
Control	CC	[redacted]	[redacted]	Y		10 μg/ml		252.31	DMSO	10.00

*Control compound CA: We started Batch 1 with 10 μg/ml; however, considering that the viability of 10 μg/ml of CA fell around the borderline of the cutoff, from batch 2 on, we decided to reduce the concentration of CA to 5 μg/ml.

Step 1: Prepare the concentrated working solution (CWS) and vehicle control solution (VCS)

1) This step is performed right before the cell treatment.

2) Table 2 describes the dilution procedure to make 100X CWS of the high concentration for each compound.

3) Prepare the corresponding vehicle control solution (VCS). For example, VCS of compound A is prepared by adding 2.5 µl of DMSO (vehicle of compound A as shown in Tables 1 and 2) into 1 ml of culture medium.

Table 2. Concentrated working solution (CWS) preparation.

Blinded Code	To prepare concentrated working solution (100X) in culture medium
**A**	Add 2.5 µl of stock into culture medium to make a total of 1 ml (400X)
**B**	Add 10.3 µl of stock into culture medium to make a total of 0.5 ml (48.52X)
**D**	Add 2.5 µl of stock into culture medium to make a total of 1 ml (400X)
**E**	Add 50 µl of stock into 450 µl of culture medium to make a total of 0.5 ml (10X)
**F**	Add 100 µl of stock into 400 µl of culture medium to make a total of 0.5 ml (5X)
**G**	Add 75 µl of stock into 225 µl of culture medium to make a total of 0.3 ml (4X)
**I**	Add 150 µl of stock into 150 µl of culture medium to make a total of 0.3 ml (2X)
**J**	Add 150 µl of stock into 150 µl of culture medium to make a total of 0.3 ml (2X)
**K**	No further dilution from the Stock solution.
**N**	Add 5 µl of stock into culture medium to make a total of 0.5 ml (100X)
**O**	Add 10 µl of stock into culture medium to make a total of 0.5 ml (50X)
**P**	Add 25.4 µl of stock into culture medium to make a total of 0.5 ml (19.67X)
**Q**	Add 10 µl of stock into culture medium to make a total of 0.5 ml (50X)
**R**	Add 10 µl of stock into culture medium to make a total of 0.5 ml (50X)
**CA**	Add 25 µl of stock into 75 µl of culture medium to make a total of 0.1 ml (4X)
**CB**	No further dilution from the Stock solution.
**CC**	Add 50 µl of stock into 450 µl of culture medium to make a total of 0.5 ml (10X)

Step 2: S9 preparation

1) Mix Regensys A (13.5 ml) with Regensys B (1.5 ml, lyophilized): take 1 ml of cold Regensys A solution to B to reconstitute B, and transfer the dissolved B back to bottle A which results in 15 ml of [A+B mixture]. (Stored at 4°C)

2) Reconstituted lyophilized S9: add 2.1 ml of sterile H2O to reconstitute S9 and the aliquots can be stored at -20°C.

3) Make fresh 10 ml of 10% S9 working solution right before cell treatment that requires S9: add 1 ml of reconstituted S9 in 9 ml of [A+B mixture].

4) The final concentration of prepared S9 for cell treatment is 1% (i.e., add 200 µl of 10% S9 to 2 ml/well for cell treatment).

Step 3: Cell treatment (outlined here, see also SOP in section 3):

To start cell treatment, add CWS into wells containing 2 ml of cells. For all compounds except K and CB, for high, medium, and low concentration, add 20 µl, 6.67 µl, and 2.22 µl CWS into each well respectively; for compound K, add 31.25 µl for high, 10.42 µl for medium, 3.47 µl for low; for compound CB, add 51.79 µl in each well. Add 20 µl of VCS into wells designated as vehicle control, mix well by moving the plate gently back and forth; for compound B, no VCS is needed to add into the cells.

The suggested plate layout is shown below. The first digit would be the blinded code of the compound, followed by '-VC', '-H', '-M', and '-L', which indicates vehicle control, low, medium, and high concentration respectively. The last digit designates the three replicates per treatment condition.

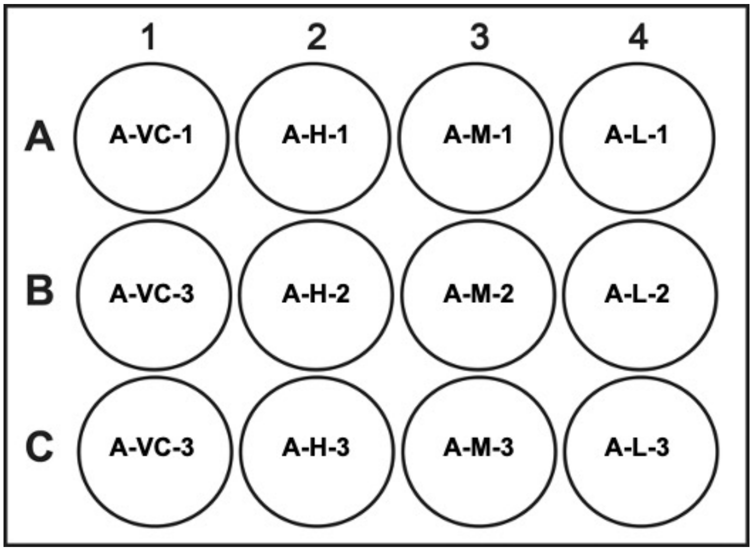

Figure 1. The suggested plate layout (compound A as an example).

We suggest running 14 test compounds in four batches; the compounds and the corresponding controls are shown below:

Batch #1: 3 compounds: F, G, O; CA&CB
Batch #2: 4 compounds: B, A, E, I; CA&CB
Batch #3: 4 compounds: D, J, K, N; CA&CB
Batch #4: 3 compounds that need S9 activation: P, Q, R; CC

Section 3. TK6 Cell Culture and Treatment SOP

Purpose

This SOP provides details and background for exposing TK6 cells to chemicals for transcriptomic analysis using the nCounter assay. This protocol will describe how to handle hazardous material and expose TK6 cells to chemicals in various concentrations.

Equipment

- Incubator with CO_2 gas chamber 37°C
- Biological safety cabinet
- Water bath
- Refrigerator
- Tissue culture flask (polypropylene)
- Microtiter plate centrifuge
- CO_2 cylinder
- Pipet aid
- NALGENE™ Cryo 1°C Freezing Container

Cell Medium Volume Requirements

Vessel	Total Medium Volume
T-25 flask	3–5 mL
T-75 flask	10–15 mL
12-well plate	2 mL/well
96-well plate	100–200 mL/well

1. Precaution

1.1. Handling of Hazardous Material

This section provides general guidelines to be followed when working with hazardous material.

1) General Work Procedures

   a. All items should be placed in the Biological Safety Cabinet (BSC) before work begins.

   b. The BSC should be cleaned before and after use with a mild disinfectant and water.

   c. Work should be done in the middle of the hood to prevent contamination and to protect the employee.

d. All contaminated equipment should be wiped clean with a mild detergent and water.

e. Needles and syringes must be placed in a sharps container labeled "Hazardous Material Are (use/storage/waste)" without being bent, recapped, or removed.

f. Hands should be washed before gloves are put on and after removal of gloves.

2) Designated Work Area

a. All hazardous chemicals should be used in a centralized area.

b. Hazardous material spill guidelines should be available in the work area.

c. The MSDSs should be provided for the chemicals used in this area and they should be kept in the area as well.

3) Biological Safety Cabinet (BSC)

a. All hazardous material work should be conducted in a Class II Biological Safety Cabinet. The blower on the cabinet should be left on at all times.

b. Decontamination should consist of surface cleaning with water and detergent followed by thorough rinsing. The use of detergent is recommended because there is no single accepted method of chemical deactivation for all agents involved.

c. Ethyl alcohol or 70% isopropyl alcohol may be used with the cleaner if the contamination is soluble only in alcohol. Alcohol vapor build-up has also been a concern, so the use of alcohol should be avoided in BSCs where air is recirculated.

4) Personal Protective Equipment

a. Double gloving is required. Double gloving with nitrile or neoprene is acceptable. Gloves should be changed immediately if torn, punctured, or contaminated.

b. Safety goggles should be cleaned with a mild detergent and water for reuse.

c. Personal Protective Equipment (PPE) used for preparation will not be worn outside of the preparation area.

d. All disposable PPE will be discarded in the "Hazardous Material Area

(use/storage/waste)" container in the work area.

1.2. Biosafety

In this SOP, we only deal with the TK6 cell line, which should be handled in the a BSL-2 biosafety cabinet. Treat all cells and media as though they are infectious (i.e., wear gloves, autoclave all spent material, etc.).

1.3. Other Safety

Avoid looking into the hood when the UV light is turned on.

1.4. During Assay

1) Use sterile techniques at all times.

2) Wash your hands and arms with soap before putting gloves on and touching tissue culture flasks and reagents.

3) Turn on the UV light and air 10 min prior to working in the hood.

4) Immediately before working and after working, wipe the working surface of the hood with 70% disinfecting solution.

5) Do not place anything in the tissue culture hood unless you wipe the surface with 70% ethanol first.

6) Keep the incubator very clean; clean the interior surface with alcohol and avoid having any drops of media or contaminated water in it. Make sure to only use distilled water to fill the incubator gaskets. Any contamination within the hood will affect culture growth and would be very difficult to get rid of once accumulated.

7) Media bottles should never be opened outside the hood. Make sure to close it tightly and seal the rim with additional paraffin to prevent introduction to contamination.

8) Cells must not be allowed to overgrow.

2. Procedure

2.1. Materials

- Analytical balance
- Appropriate container for storing stock solution of chemicals

- TK6 cells (ATCC® CRL-8015™) to be provided (maintained at the cell concentration between 2 x 10^5 and 1 x 10^6 cells/ml).
- Tissue culture flasks (T-75), 12-well plates, or 96-well plates
- Serological pipets, sterile
- RPMI 1640 medium (RPMI) (Gibco Thermo Fisher catalog no. 11875093)
- Fetal bovine serum (FBS), certified, US origin (Gibco Thermo Fisher catalog no. 16000044)
- Gentamicin (50 mg/ml) (Gibco Thermo Fisher catalog no. 15750078)
- Growth medium: RPMI supplemented with 10% inactivated FBS and 50 μg/ml of gentamicin
- 1X PBS without calcium and magnesium (https://www.fishersci.com/shop/products/hyclone-phosphate-buffered-saline-pbs- 9/SH3025601 or the equivalent)
- S9 mix and NADPH regenerating system: Moltox LS9 SD Phenobarbital/Benzoflavone (Male rat liver in 0.15M KCl., 2.1ml, product number 11-05L.2); Regensys™ NADPH Regenerating System Reagents (product number 60-200.15 & 60-201.15L)
- MTT Cell Proliferation Assay Kit (Cayman Chemical, item no. 10009365)

2.2. Maintenance of TK6 Cell Culture

1) Use T75 culture flasks for the cell line maintenance.
2) When thaw cells from the frozen cryotube, thaw frozen cells rapidly (< 1 minute) in a 37°C water bath. Transfer into a 15-ml conical tube with 10ml pre-warmed growth medium inside a laminar flow hood; centrifuge the tube at 1000 rpm for 5 min at 4°C.
3) Remove and discard supernatant; resuspend cells in 5ml of growth medium. Transfer the cell suspension into the T75 culture flask with vented caps and add 10 ml more growth medium to the flask.
4) Incubate flasks in 37°C incubator with 5% CO_2.
5) Observe cells daily under an inverted microscope and maintain the cell concentration between 1 x 10^5 and 1 x 10^6 cells/ml. When cell concentration gets close to 1 x 10^6 cells/ml, pass cells into a new culture flask by diluting the cells to 2 x 10^5/ml.
6) Note: It may take 2 passages before TK6 cells reach their normal growth rate. Once TK6 cells reach their normal growth rate (~16hr for doubling), expand for one more passage before seeding for toxicogenomics.

7) Cryo-preservation of cell stocks:
   1. When culturing, it is crucial to store some of your passaged cells for future usage to avoid wasting cells of lower passages. Cryo-preservation medium contains 80% growth medium, 10% FBS and 10% DMSO.
   2. Inside a laminar-flow hood, transfer cell suspension into a 15 ml conical tube; centrifuge the tube at 1000 rpm for 5 minutes at 4°C
   3. Wash cells with 10 ml of 1X PBS.
   4. Centrifuge at 1000 rpm for 5 min at 4°C.
   5. Under sterile conditions discard supernatant; resuspend cells in cryo-preservation media.
   6. Aliquot 1 ml of cell suspension into well labeled cryovials carrying the following information: [cell line- passage- date cryopreserved- initials of technician working].
   7. Place cryovials in slow freezing container (NALGENETM Cryo 1°C Freezing container) with a cooling rate of -1°C/min.
   8. Place freezing container in -80°C for 24hrs, then transfer in liquid nitrogen for long term storage.

2.3. Preparing Cells for Chemical Treatment

1) The day before treatment, count the cell number and dilute cells to 2 x 10^5 cells/ml and seed 2 ml per well in 12-well plates for overnight incubation.

2) On the day of treatment, for each chemical, treat cells at each concentration in triplicate alongside solvent control as shown in Figure 1.

3) To decide the concentrations used in the nCounter assay, the highest concentration will be at which cells survived higher than 40% based on MTT assay.

2.4. Cell Viability Using MTT Proliferation Assay and Cell pellet collection

Treatment without S9:

1) In studies without S9, treat cells at selected concentrations with chemicals and corresponding controls, and harvest cells at 4 hr post-treatment. The positive and negative controls provided should be included in each batch.

2) After 4-hr treatment, transfer cell suspension from each well of 12-well plates to three wells of 96-well plates with 100 µl per well. Centrifuge the 96-well plates at 1000 rpm for 5 min, discard the supernatant and add 100 µl of fresh medium and incubate in 37°C incubator with 5% CO_2 for an additional 20 hr.

3) After 20-hr recovery, perform MTT Assay following the manufacturer’s instructions.

Treatment with S9:

4) The positive control for experiments + S9 (i.e., benzo[a]pyrene) should be included.

5) For agents that require activation, S9 with NADPH generating system cofactors is added to the cells right before the treatment at 1% (v/v), followed by adding the compound solutions and corresponding solvent controls at selected concentrations. The cells are exposed for 3 hrs.

6) After 3hr-treatment in presence of S9 and compounds, transfer cell suspension from each well of 12-well plates to three wells of 96-well plates with 100 µl per well. Centrifuge the 96- well plates at 1000 rpm for 5 min, discard the supernatant carefully using a multichannel pipette and add 100 µl of fresh medium and incubate in 37°C incubator with 5% CO_2 for an additional 24 hrs. After 24-hr recovery, perform MTT Assay following the manufacturer's instructions.

7) Replace the treatment medium with fresh medium (1.7 ml/well) for the remaining cells in 12- well plates and incubate for an additional 4 hrs. Harvest cells for RNA extraction.

Cell Pellet Collection (for both treatments with and without S9):

8) To harvest cells, collect cells with medium in 15 ml conical tubes, centrifuge at 1000 rpm for 5 min, carefully remove medium, gently resuspend cells in 1 ml of 1X PBS without calcium and magnesium and transfer to an Eppendorf tube. Centrifuge again using the bench top centrifuge at 4,000 rpm for 5 min and remove PBS. Cell pellets should be stored at –80°C until RNA extraction.

Note*: Cells should be collected for measurement of transcriptional changes 4 hr following the exposure as later collection times may be suboptimal, potentially reflecting secondary chromosomal damage caused by factors such as cytotoxicity and cellular stress rather than the primary transcriptional response to the test compound.*

Section 4. NanoString SOPs

4a. RNA Extraction from Cell Culture

1. Purpose

This SOP provides details and background for extracting RNA from cultured cells. This protocol describes the RNA extraction procedure using the Qiagen RNeasy Mini Kit.

2. Precaution

2.1. Biosafety

Although clean cell culture has a requirement of being pathogen free, some cell lines are carcinoma-like and must be treated as infected. In this SOP we only deal with the TK6 cell line, which is not associated with cancers. Treat all cells and media as though they are infectious (i.e., wear gloves, autoclave all spent material, etc.).

2.2. During Assay

Maintaining sterility is critical throughout the assay to prevent contamination. Proper storage of samples and RNA at -80°C is essential to avoid RNA degradation. Adherence to RNA sterile handling techniques is mandatory for TGx-DDI analysis. This includes ensuring that work areas, equipment, and consumables are RNase-free and using certified RNase-free reagents and materials.

3. Materials

- RNeasy Mini Kit (Qiagen no. 74106)—250 RNeasy columns
- QIAShredder (Qiagen no. 79656)—250 columns
- Ethanol (96–100%)
- 14.3 M β-mercaptoethanol (β-ME)
- RNase-free H_2O (included in the RNeasy Mini Kit)
- Microcentrifuge

4. Procedure

4.1. Important Notes Before Starting

1) Cell pellets can be stored at –80°C for later use or used directly in the procedure.

Note*: If the sample is stored for more than one month at -80°C and subjected to more than one freeze and thaw, the RNA quality (Section 4b) must be reassessed to confirm that it meets the required criteria before the sample is used in further experiments.*

2) Frozen cell pellets should be thawed lightly so that cell pellets can be dislodged by flicking.

3) Buffer RLT and Buffer RW1 contain a guanidine salt and are therefore NOT compatible with disinfecting reagents containing bleach. Guanidine is an irritant. Take appropriate safety measures and wear gloves when handling.

4) β-ME must be added to Buffer RLT before use. β-ME is toxic; dispense in a fume hood and wear appropriate protective clothing. Add 10 µl of β-ME per 1 ml of Buffer RLT. Buffer RLT is stable for 1 month after addition of β-ME.

5) Buffer RPE is supplied as a concentrate. Before using for the first time, add 4 volumes of ethanol (96–100%) as indicated on the bottle to obtain a working solution.

4.3. Isolation of Total RNA From Cells

1) Loosen the cell pellet thoroughly by flicking the tube, adding 350 µl of Buffer RLT. Vortex or pipet to mix and proceed to step 2.

Note: Incomplete loosening of the cell pellet may lead to inefficient lysis and reduced RNA yields.

2) Homogenize the lysate using a QIAshredder spin column. Pipette lysate directly into a QIAshredder spin column placed in a 2-ml collection tube, and centrifuge for 2 min at full speed (14,000 rpm).

3) Add 350 µl of 70% ethanol to the homogenized lysate and mix well by pipetting. ***Important:*** Do not centrifuge.

4) Transfer up to 700 ml of the sample, including any precipitate that may have formed, to a RNeasy spin column placed in a 2-ml collection tube (supplied). Close the lid gently, and centrifuge for 15 s at ≥ 8000 × g (≥ 10,000 rpm). Discard the flow-through. Reuse the collection tube in step 6.

5) Add 700 µl of Buffer RW1 to the RNeasy spin column. Close the lid gently, and centrifuge for 15s at ≥ 8000 × g (≥ 10,000 rpm) to wash the spin column membrane. Discard the flow-through. Reuse the collection tube in step 6.

Note: After centrifugation, carefully remove the RNeasy spin column from the collection tube so that the column does not contact the flow-through. Be sure to empty the collection tube completely.

6) Add 500 ml of Buffer RPE to the RNeasy spin column. Close the lid gently, and centrifuge for 15s at ≥ 8000 × g (≥ 10,000 rpm) to wash the spin column membrane. Discard the flow-through. Reuse the collection tube in step 7.

Note: Buffer RPE is supplied as a concentrate. Ensure that ethanol is added to Buffer RPE before use (see section 4.1, "*Important Notes Before Starting*").

7) Add 500 µl of Buffer RPE to the RNeasy spin column. Close the lid gently, and centrifuge for 2 min at ≥ 8000 × g (≥ 10,000 rpm) to wash the spin column membrane. The long centrifugation dries the spin column membrane, ensuring that no ethanol is carried over during RNA elution. Residual ethanol may interfere with downstream reactions.

Note: After centrifugation, carefully remove the RNeasy spin column from the collection tube so that the column does not contact the flow-through. Otherwise, carryover of ethanol will occur.

8) Place the RNeasy spin column in a new 2-ml collection tube (additional collection tubes can be ordered from Qiagen with Cat. No. / ID: 19201 if the ones provided in the kit run out) and discard the old collection tube with the flow-through. Close the lid gently, and centrifuge at full speed for 1 min. Perform this step to eliminate any possible carryover of Buffer RPE, or if residual flow-through remains on the outside of the RNeasy spin column after step 7.

9) Place the RNeasy spin column in a new 1.5-ml collection tube (supplied). Add 40 ml of RNase-free water directly to the spin column membrane. Close the lid gently, and centrifuge for 1 min at ≥ 8000 × g (≥ 10,000 rpm) to elute the RNA.

10) Measure RNA concentration using Nanodrop. Record the 260/280 ratio as well.

11) Store RNA at -80°C and avoid repeated freeze-thaw cycles.

4b. RNA Quality Control

(This and the following sections are not needed for the laboratories sending out samples to CRO for NanoString service)

Purpose

The Agilent RNA 6000 Nano Bioanalyzer kit is used along with the Agilent 2100 Bioanalyzer to assess RNA quality by obtaining an RNA Integrity Number (RIN). This number reflects the degree of degradation of RNA using a scale of 1–10. The quality of the RNA sample is crucial to the success of subsequent genomic experiments involving the use of RNA.

Supplies Needed (All NanoString supplies were ordered by GU and sent to the user directly from the manufacturer – See section 1a)

- RNA 6000 Nano Assay Kit (12 samples per chip) Chip Priming Station
- IKA Vortex Mixer (Blue)
- RNase Zap (Ambion)
- RNase-free water
- Pipettes (10 µl, 200 µl, 1000 µl) with RNase-free tips
- 0.5 ml or 1.5 ml centrifuge tubes (RNase-free) compatible with heat block
- Heat block for ladder/sample denaturation
- Timer
- Microcentrifuge (≥ 1300 × *g*)
- Total RNA samples (25–500 ng/µl)
- RNA Nano Ladder

*****Note:*** Be sure that benchtop, pipettes, and equipment have been treated with RNase Zap before beginning assay. Always wear gloves and change gloves often to avoid contamination of reagents and supplies with RNases.**

Before Starting

- Remove kit reagents from refrigerator and store at room temperature for 30 min before using (protect from light).
- Put Nano Ladder aliquot (1.3 µl for each chip) and samples on ice to thaw.
- Turn heat block to 70°C.
- Turn on bioanalyzer and start up computer and 2100 Expert software.
- Set up Chip Priming Station (Base Plate at position 'C' and Syringe Clip in Top notch — this

should already be set correctly).

Protocol (Adapted from RNA 6000 Nano Kit Guide)

1) Clean the Bioanalyzer
    a. Place 350 µl of RNase Zap into one well of the Zap cleaning chip (clear).
    b. Place in bioanalyzer, close lid and let sit for 1 min, remove chip.
    c. Set this chip aside for clean up after completion of experiment.
    d. Add 350 µl of RNase-free water to one well of the Water cleaning chip.
    e. Place in bioanalyzer, close lid and let sit for 10 sec, remove chip.
    f. Leave the lid to the bioanalyzer open for 10 sec before closing.
    g. Set this chip aside for clean up after completion of experiment.
2) Prepare Gel
    a. Can use previously prepared gel for up to 1 month.
    b. To prepare fresh gel:
        i. Place 550 µl of Nano Gel Matrix (Red) into spin filter tube.
        ii. Centrifuge for 10 min at 4000 rpm at room temperature.
        iii. Aliquot 65 µl into clean 0.5-ml tube labeled 'Filtered Gel.'
3) Prepare Gel+Dye Mix
    a. Vortex Nano Dye (Blue) concentrate for 10 sec and spin down.
    b. Add 1 µl of dye to 65 µl of filtered gel.
    c. Vortex and centrifuge for 10 min at 14,000 rpm at room temperature.
    d. Use this within 1 day of preparation (protect from light).
4) Loading Chip With Gel+Dye Mix

a. Open new Nano chip and place on priming station.

i. Ensure syringe clip is in top notch position.

ii. Attach syringe by screwing onto base plate.

b. Add 9 µl of Gel+Dye mix into Dark 'G' well only of chip.

c. Set timer to 30 sec.

d. Make sure syringe plunger is at 1 ml.

e. Close priming station until a 'click' is heard and quickly press down syringe plunger until it is held down by clip.

f. Start timer for exactly 30 sec.

g. Release the plunger by depressing clip release and let it slowly rise to 1 ml for 5 sec. Move plunger up slowly manually until it reaches 1 ml.

h. Pipette 9 µl of Gel+Dye mix into each of the other two wells marked with a 'G.'

5) Add 5 µl of Nano Marker (Green) into the Ladder well and into each Sample well. (For running empty sample wells, add 6 µl of Nano Marker.)

6) Heat denature the ladder and RNA samples at 70°C for 2 min. Place at room temperature, not on ice after heating.

7) Load samples onto chip; organize samples to match chip layout

a. Pipet 1 µl of ladder into Ladder well.

b. Pipet 1 µl of sample into each Sample well. Do not leave any wells empty. (Empty wells should contain 6 µl of Nano Marker.)

8) Vortex chip on vortex mixer for 1 min. (Push chip up against top bar to ensure a tight fit and vortexer has 1-min built-in timer after pushing Start.)

9) Place chip in bioanalyzer, close lid. Be sure to load into bioanalyzer within 5 min after preparation.

20

10) On Instrument Square of 2100 Expert Software, ensure all check marks are green.

11) Select Assay: Assay > RNA > Eukaryotic Total RNA Nano Assay.

12) Click on Start button.

13) Select Data Square.

14) Enter Sample Names, Comments, Kit, and Chip Lot numbers.

15) Click on Gel Tab to view samples as they are analyzed.

16) For Nano ladder well, be sure the green marker band is aligned with the 25-nt band of the ladder (if not, adjustments to the marker must be made). Six bands should appear above the green 25-nt marker band.

17) Wait for all 12 lanes to finish running and look at RINs for each sample. (A RIN of 10 denotes fully intact RNA and scores lower than 10 reflect varying degrees of degradation). RIN of greater than 7 is needed before continuing.

18) When done, immediately remove sample chip and perform the ‘Clean the Bioanalzyer’ steps as mentioned above. Do not leave the used sample chip in bioanalyzer for longer than 1 hr to avoid contamination of the electrodes. Also ‘Clean the Bioanalyzer’ in between running multiple chips. Flick out liquid from cleaning chips into sink when finished.

19) Select File > Save As to save the .xad file to proper location. Save after each chip run.

20) Select File > Print (make sure all item boxes at top are selected), check pdf box, click ‘…’ to choose location, and then click ‘Save’ to save .pdf file. Save after each chip run. RIN values must reported.

21) Place RNA 6000 Nano kit back at 4°C for storage. Cleanup workspace, equipment, and pipettes with RNase Zap.

4c. nCounter Gene Expression Analysis

1. Purpose

This protocol describes the required procedures for the analysis of nCounter® gene expression assays.

2. Scope

This procedural format is utilized by the Fornace Laboratory at Georgetown University.

3. Materials

3.1. Reagents and Hardware From NanoString

1) Customized Codeset (72 genes) (store at -80°C)
2) nCounter® Master kit (100050), including
    a. nCounter® Cartridges (12 samples per cartridge)
    b. nCounter® Prep Plate (includes wash buffers, magnetic beads, and immobilization and imaging reagents)
    c. nCounter® Prep Rack (includes racked tips and foil piercers, 12-tube strips, strip tube caps, tube sheaths, cartridge well seals, and hybridization buffer)
3) nCounter® Prep Station and Digital Analyzer
4) Memory Stick (for each custom codeset, one memory stick is provided)

Note:

- **Cartridge:** Store for up to 1 year at -20°C.
- **nCounter® Prep Plate:** Stable for up to 1 year at 4°C.
- **Hybridization Buffer & Prep Pack:** Store for up to 2 years at room temperature. Remaining Prep Pack materials, store at room temperature. No expiration date.

3.2. Other Reagents and Hardware

1) Purified total RNA (store at -80°C)
2) Milli-Q water or equivalent
3) Thermo Cycler

3.3. Required Software

1) nSolver™ Analysis software

4. Procedure

The following procedure is a modification of the NanoString nCounter® XT Assay User Manual, MAN-10023-11, July 2016 and nCounter® Analysis User Manual, MAN-C0035-05, July 2015.

4.1. Prepare Sample Hybridization (at Room Temperature)

There are 12 RNA samples for each test compound, including the vehicle control and the treatment at three concentrations. The 12 samples for each compound are loaded on one chip. Each sample includes 3 µl of Reporter CodeSet, 5 µl of hybridization buffer, 5 µl (100 ng) of sample RNA, and 2 µl of Capture ProbeSet.

1) Dilute total sample RNAs to 20 ng/µl in 96-well plate. Use total 100 ng of RNA for hybridization.

2) Remove aliquots of both Reporter CodeSet and Capture ProbeSet reagent from the freezer and thaw at room temperature. Invert several times to mix well and spin down reagent.

Note: *After it has thawed, inspect the tube of Reporter CodeSet to make sure no colored precipitate is present. If you see a colored precipitate, heat the entire tube to 75°C for 10 min and cool at room temperature before using.*

Note on RNA input: *Based on recommendations from NanoString and our experience, using a higher RNA input for NanoString analysis can enhance data accuracy, as it increases the number of data points without overloading the chip. However, we recommend to maintain a consistent RNA input across all samples to ensure reliable and comparable results.*

3) Create a master mix by adding 70 µl of hybridization buffer to the tube containing the Reporter CodeSet. Do not remove the Reporter CodeSet from this tube. Do not add the Capture ProbeSet to the master mix. Invert repeatedly to mix and spin down mastermix.

4) Label the hybridization tubes. If using strip tubes, make sure they fit in a microfuge.

5) Add 8 µl of master mix to each of the 12 tubes (one strip). Use a fresh tip for each pipetting step to accurately measure the correct volume.

6) Add 5 µl of sample RNA to each tube.

7) Invert the Capture ProbeSet tube to mix and spin down the contents. Add 2 µl of Capture ProbeSet to each tube immediately before placing at 65°C. Cap tubes and mix the reagents by inverting the tubes several times and flicking with a finger to ensure complete mixing. Briefly spin down and immediately place the tubes in the preheated 65°C thermal cycler.

Note: Minimizing the time between addition of the Capture ProbeSet and incubation at 65°C will increase assay sensitivity.

8) Incubate reactions for at least 16 hr. Maximum hybridization time should not exceed 48 hr. Ramp reactions down to 4°C and process the following day. Do not leave the reactions at 4°C for more than 24 hr or increased background may result.

Note: The purpose of selecting a fixed hybridization time followed by a ramp down to 4°C is to ensure equivalent hybridization times of all assays being directly compared in the same series of experiments. Counts continue to accumulate with time, with total counts typically increasing 5% per hour between 16 and 24 hr. Although a 16-hr incubation is adequate for most purposes, a longer incubation will increase sensitivity by increasing counts without significantly increasing background.

4.2. Set up nCounter® Prep Station

1) Prior to processing, cartridges and Prep Plates must be at room temperature.

   a. Remove the nCounter Prep Plates from storage at 4°C and the nCounter Cartridges from storage at -20°C. Allow them to equilibrate to room temperature for 10–15 min.

   b. Centrifuge the Prep Plates at 2000 × g for 2 min to collect all liquids in the bottom of the wells prior to loading the Prep Plates onto the Prep Station deck. After centrifugation, visually inspect plates to ensure that reagents have collected at the bottom of each well. If not, repeat the centrifugation step. If the problem persists, slowly invert the plate several times to resuspend the beads into the liquid reagent and repeat the centrifugation step.

2) Turn on both the Prep Station and Digital Analyzer, select “Life Sciences” mode.

3) On the Main Menu, select “start processing” to set up a new run.

   >>> The ‘Select Protocol’ screen will appear.

4) Select “High Sensitivity” from three protocols for processing samples (High Sensitivity, Standard, or Legacy Protocol). Press next.

   >>> The ‘Sample Selection’ screen will appear.

5) Select the sample positions that will be processed. Blue tubes will be processed, and grey tubes will not be processed. If processing fewer than 12 samples, begin with sample 1. Press next.

   >>> The ‘Warm Reagents & Cartridge’ screen will appear.

6) Load the Prep Plates onto the Prep Station deck. Visually inspect plates to ensure that reagents have collected at the bottom of each well. Press next.

24

>>> The ‘Waste Receptacles’ screen willappear.

7) Remove the liquid waste container from the combined receptacle and dispose of the liquid appropriately. Tips should be discarded into the appropriate container as outlined in Lab Safety Manual. Press next.

>>> The ‘Reagent Plate’ screen will appear.

8) Remove the clear plastic lids and place the Prep Plates on the deck. Press next.

>>> The ‘Tips & Foil Piercers’ screen will appear.

Note: Do not remove the foil or pierce the wells on the reagent plate. The Prep Station will pierce the wells during processing. The Prep Plate should be oriented with the label facing the user. If the plate is placed in the wrong direction, the Prep Station will pause the protocol until the user intervenes.

9) Remove the metal tip carrier from the Prep Station deck by lifting straight up. Place the tips and the foil piercers into the carrier. It is helpful to place the carrier at eye level to align the plastic tips in the carrier.

10) Replace the loaded tip carrier back onto the Prep Station deck with the grey foil piercers closest to the user. Press next.

>>> The ‘Tip Sheaths’ screen will appear.

11) Place the tip sheaths on the deck and press firmly into place. Press next.

>>> The ‘Sample Cartridge’ screen will appear.

12) Open the cartridge wrapping (keep it for record), pay attention to not break the seed drying beads package and label the top and bottom of the cartridge. Properly place the cartridge on deck (top of the cartridge on the left side, hole of the cartridge at the top). Press next.

>>> The ‘Electrode Fixture’ screen will appear.

13) Carefully lower the electrode fixture in place over the cartridge. Press next.

>>> The ‘Empty Strip Tubes’ screen will appear.

14) Place the empty strip tubes on the deck. Confirm tube 1 lines up with position 1 on sample

holder. Close tube holder. Press next. **Only use strip tubes provided by NanoString**. Other tubes have different dimensions and will cause system failure.

>>> The 'Hybridized Samples' screen will appear.

15) Take out the sample strip from the thermocycler, quickly spin down and visually check the volume of each tube and **open the lid** and place it on the deck of Prep Station, ensuring tube 1 lines up with position 1 on sample holder and close tube holder. Press next.

>>> The 'Notification Options' screen will appear.

Note: The strip tube is asymmetrically keyed, and if the strip tube is placed incorrectly, the lid will not close properly and the Prep Station will not be able to start processing. Do not let the hybridized samples sit at room temperature longer than 15 min. If there is a delay before starting the run, store hybridizations with nCounter CodeSets at 65°C until they can be processed.

16) Enter email address. Press enter.

17) Finally, select whether the Prep Station should make an audible alarm when processing is finished. When all alerts have been set, press next.

>>> The 'Start Processing' screen will appear.

18) Press start when ready to begin processing.

>>> The 'Validating deck layout' screen will appear.

Note: The nCounter Prep Station will first check that all consumables and reagents have been placed properly on the deck. To do this, the Prep Station confirms that the sensors for the sample cartridge, electrode fixture, and heater lid are all in the correct state. The pipette head then checks that tips, tip sheaths, strip tubes, and Prep Plates are all in place by touching them with a set of validation tips. Do not be alarmed that the Prep Station is touching the consumables; this is a part of normal operation. If the Prep Station determines that a consumable is misplaced, it will instruct the user to adjust the configuration. The 'Validating deck layout' screen will eventually update to the 'System Processing' screen. Both screens display the current time of day and the estimated time of day that the run will complete. They also provide the option to pause the run.

19) When the run is complete, the blue 'System Processing Complete' screen will appear, and the timer will count up

20) Press next.

>>> The 'Run Successfully Complete' screen will appear.

21) The 'Run Successfully Completed' screen lists the steps to follow once the run is complete, including:

    a. Remove and discard empty reagent plates.

    b. Remove and discard the empty tip racks and foil piercers.

    c. Remove and discard the sample strips.

    d. Remove the sample cartridge and seal the wells.

22) To release the fixture after the run is complete, press the lever in the center top of the device toward the front with a finger.

23) After processing is complete, it is important to do the following:

    a. Seal the wells immediately with the adhesive film provided to prevent evaporation.

    b. Samples should be protected from as much light as possible. Store them in the dark to prevent photobleaching.

    c. Store samples in the refrigerator at 4°C. Once sealed, samples can be stored at 4°C for up to a week with minimal degradation.

    d. Empty the waste containers.

24) Press finish to return to the Main Menu.

4.3. File Structure of nCounter Analysis System

There are three types of files used by the nCounter Analysis System: the Cartridge Definition File (CDF), the Reporter Library File (RLF), and the Reporter Code Count (RCC) file.

Each custom CodeSet ships with a USB flash drive containing the following files for use on the Digital Analyzer:

- Three read-only folders for Cartridge Definition Files (CDFData), Reporter Library Files (RLFData), and Reporter Code Count files (RCCData)

27

- A CDF template

- A compressed file containing the nSolver Analysis Software installer for Microsoft® Windows®, a PDF copy of the nSolver Analysis Software Manual, and a folder containing sample RCC files

- Product-specific assay manuals, product inserts, and data analysis guides in PDF format

- The nCounter Analysis System User Manual

- Material Safety Data Sheet(s)

1) The CDF is created by the user and defines sample-specific data to associate with the data output. It also defines the parameters for the imaging instrument to use during image collection and processing.

2) The Reporter Library File (RLF) is generated by NanoString and is unique to each custom codeset. It contains the information used during image processing to assign target identities to the barcodes.

3) The Reporter Count Code (RCC) is generated by the digital analyzer and contains the data for one of the 12 flow cells (assays) in a cartridge, detailing the number of counts for each target.

4.4. Cartridge Definition Files Preparation

One CDF document is created per one cartridge (12 samples) run on the Prep Station. All CDFs have to be saved under the “CDFdata” folder of the NanoString USB stick. Data contained in the CDF include Sample ID, Owner ID, Comments, and Reporter Library File (RLF).

1) Using Excel, open the .csv template on the USB flash drive that comes with the Codeset.

2) Write the cartridge ID and your email if you have email capability enabled on the Digital Analyzer and would like email delivery of your data.

3) Leave the “ArchiveFolder” blank

4) Write the samples ID in the good order according to the lane ID. Use thecorresponding working list to do so.

5) Under the column “Owner”, write “Your Name”.

6) Under the column “Comments”, write the project name.

7) Under the column “Date”, write the date of the Prep Station run.

8) FOVCount — This field specifies the number of images (fields of view) to analyze per assay, which corresponds to the amount of data to collect. Check that “555” is present for all lanes of the column “FovCount”.

9) Write the name of the Reporter Library File (RLF) that will be used (can be found in the “RLFData” folder of the NanoString USB stick).

10) GeneRLF — This field defines the RLF to associate with the data. It is extremely important that this filename be correct or data will be misinterpreted. The “.rlf” file type extension should NOT be used here.

11) Save the document as with the strip ID name, and keep the .csv extension. The file name will include BOTH “.cdf” and “.csv” extensions

12) Open the document with Notepad and check that the separators are all commas. If not, replace all the semi-colons with commas using the “replace” tab.

13) “Manually” change the extension of the document from .csv to .cdf. Ensure that the created .cdf is located in the “CDFdata” folder of the memory stick.

4.5. Uploading RLF and CDF Files

1) Uploading RLFs on the digital analyzer is required when receiving a new codeset. Once uploaded, it will always be available until manually deleted from the instrument.

    a. Insert the USB drive and from the main menu press “upload files”.

    b. Press “upload RLF” and the select RLF source screen appears.

    c. The folder and RLF files from the memory stick that have not been uploaded yet will display as selected.

    d. Select the appropriate RLF and press “next” to upload the file that is saved on the local analyzer drive.

2) Uploading CDF file

a. Insert the USB drive, and from the main menu press “upload files”.
b. Press “upload CDF”, and the select CDF source screen appears.
c. Browse to the location on your memory stick where the CDF file is stored. Press “next” and the select CDF destination screen appears.
d. Browse to the folder location on the analyzer where you want to save the CDF and press “Save”.

4.6. Initiating a Run

1) From the Main Menu, select start counting.

   >>> The ‘Select Stage Position’ screen will appear.

2) Select the cartridge position for which cartridge information will be entered by touching that cartridge position on the screen. The selected cartridge will appear in green. If the wrong cartridge is selected, touch the correct position and the active cartridge will display in the new position. Press next.

   >>> The ‘Select Cartridge Definition Mode’ screen will appear.

3) On the select cartridge definition mode screen, press load existing.

   >>> After pressing load existing, the ‘Select CDF’ screen will appear.

4) Select the CDF to be used and press next.

   >>> The ‘Cartridge information’ screen will appear.

5) Check that all information is correct and press “done”. You are now back to the “select stage position” screen.

6) Repeat steps 2 to 5 as much as necessary depending on how many cartridges have to be read by the digital analyzer.

7) Wipe the bottom of all cartridges with a lint-free tissue. Place cartridges into the stage position corresponding to the data entered in steps 2–7 above. Be sure that each cartridge is seated flat in the slot. Close the magnetic clips gently.

8) Proceed to a last check: select the stage position to see if the ID on the screen is the same as

the cartridge ID inside the machine.

9) Close the instrument door. Press “done” and then “start” on the initiate imaging screen.

10) Once imaging begins, the counting cartridge ID screen displays the following information:

    a. The cartridge ID for the active cartridge (the cartridge currently being scanned)

    b. Cartridge scan status/progress
        - Blue — cartridge scan completed and/or in progress
        - Green — cartridge yet to be scanned during the run
        - Clear (white) — cartridge position for which no data have been defined

    c. Real-time data on the status of data collection
        - Current time — the current time of day as defined in the system setup utility
        - Time left (#) — the approximate amount of time to complete the active cartridge
        - Time left (all) — the amount of time to complete all cartridges
        - Finish time — the time of day the run will be finished (current time + total run time)

11) From the counting cartridge screen, it is possible to pause the run to add additional cartridges by pressing “pause” and then “add cartridge” etc. (steps 2 to 5).

12) When the data collection has completed, the stage will move into a position for the door to be opened and cartridges removed. Take out the read cartridges, wrap them in aluminum foil, and store them at 4°C for up to 2 to 3 months.

13) If the analyzer is connected to the network, RCC files will be received by email or they can be transferred to a memory stick (plugged in the analyzer) by pressing “download data” from the main menu. Select the data of interest and press again “download data”.

14) If any problem occurs during the run, contact NanoString support and send them the log file. To access the log file, insert the USB drive in the Prep Station and on the main menu press “maintenance”, “troubleshoot”, and then “download log files”. Select the run of interest and download it.

15) Turn off the instrument by pressing “shutdown system” into the maintenance menu. Press “Yes”.

Section 5. Data Analysis SOP (Using CEBS TGx-DDI Biomarker Tool)

Quality assurance/Quality control (QA/QC)

The 'RCC data' file produced by the NanoString Instrument contains the raw data. The analysis pipeline should begin with platform specific QA/QC of raw sequencing reads to assess read quality, using the set of QA/QC parameters established in the analysis pipeline of the NanoString nSolver™ Analysis Software. Samples that fail to meet the QC criteria or generate QC flags should be excluded from further analysis. A comprehensive instruction for examining the QA/QC flags generated by the software is provided in Appendix I.

Prior to proceeding, ensure there are sufficient solvent controls and experimental samples (at least n=2) to proceed with the analysis for each compound.

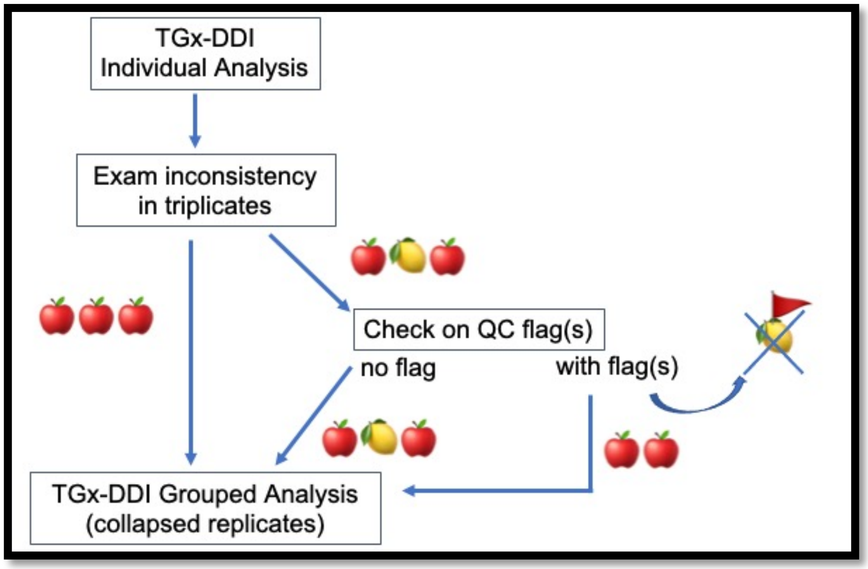

Figure 2. Ring-trial data analysis flowchart.

If a sample has to be removed, the analysis can continue with the rest of the samples if at least two out of three replicates from that treatment group are retained. If two or more samples are excluded from a triplicate, the sequencing assay should be re-run using samples that pass the quality check and cytotoxicity thresholds. All samples in the experiment should be repeated. If the remaining RNA is not enough for the repeats, or the RNA quality does not meet the specified criteria (e.g., RIN > 7), the entire experiment should be repeated starting from the cell culture step. In a case that the samples fail to meet the cytotoxicity thresholds, it is recommended to refine the dose selection by repeating range-finding experiments.

Instructions for the NIEHS/CEBS Classification Tool

Data are normalized by using the geometric mean of positive control counts in the codeset. The normalized data are exported as gene counts; fold change is calculated and log2 transformed and saved as a tab-delimited text file. Gene Symbols should be in the first column, followed by the log2 fold changes in subsequent columns that are labeled according to data type (e.g., chemical) and/or concentration. The data are submitted to the online tool (https://cebs.niehs.nih.gov/tgxddi/tool) using the Generic Array option or using the Batch Data option from the Microarray Platform drop menu. Detailed instructions for using the nSolver and NIEHS/CEBS web tool for TGx-DDI data analysis are provided in Appendix II.

For Generic Array

Probe	Chemical A conc. 1
GADD45A	-1.577
CDKN1A	0.006
.	.
.	.
.	.
TRIM22	0.847

For Batch Data

Probe	Chemical A conc. 1	Chemical A conc. 2	Chemical B conc. 1	Chemical B conc. 2
GADD45A	-1.577	0.144	0.387	1.618
CDKN1A	0.006	0.099	-0.132	-0.082
.	.	.	.	.
.	.	.	.	.
.	.	.	.	.
TRIM22	0.847	0.122	-0.525	1.102

These data files are uploaded to the TGx-DDI biomarker tool (see Appendix II for details).

1) In the "Study Information" section, select any option for the data fields displayed.

2) Select the Microarray platform.

- Generic Array — The following fields are required:

- Test Chemical Name
- Concentration
- Graph Label Name
- The $\log_2$ Ratio column (default is 2)

- Batch Data — The following fields are required:

    - Test Chemical Name

3) Choose the Generic File or Batch File to be uploaded.

4) Click [Submit] to begin data analysis.

5) Review this information in the table for accuracy and completeness, then click [Start Process] to begin data analysis.

6) In the "Study Information" section, select any option for the data fields displayed or use default information displayed.

7) Clicking [Submit] now displays a table showing the results (Data Files, Plot Files, Predicted Class, Estimated Probabilities of DDI and non-DDI with the Study Information).

8) Downloading all files and the result table or adding new data can be done from this page.

9) Review this information in the table for accuracy and completeness.

Section 6. Data Interpretation SOP (Making a DDI or Non-DDI call)

The calculation of DDI probability by the online tool is solely based on the nearest shrunken centroid probability classifier (PA). To apply the three-pronged approach, users should examine the principal component analysis (PCA) and hierarchical cluster analyses (HCA) by clicking on the clustering link in the online tool.

Overall Classification

The overall DDI classification was derived through a step-by-step process:

1. At the sample level, each experimental sample received a classification call. If a positive DDI call was made in any of the analysis methods (PA, PCA, HCA) in the three-pronged approach, the sample was classified as DDI. If all the methods generated a non-DDI call, the compound was classified as non-DDI. Compounds may produce an 'inconclusive' call under specific conditions: when the PA probability is below 0.9 for either DDI or non-DDI classification, when the compound falls on the PC1 axis, or if the compound does not fall distinctly on either the DDI or non-DDI branch of the dendrogram. For samples without a definitive DDI classification, a majority rule was used to classify them as non-DDI or inconclusive.
2. A grouped analysis was then conducted to classify each concentration of a compound. As with the classification of individual samples, if a majority of samples (e.g., two out of three) within a concentration produced a DDI call, the concentration was called DDI in the 'grouped analysis'. Similarly, samples were classified by the majority rule as either non-DDI or inconclusive.
3. Finally, a compound was classified as DDI if a DDI call was made at any concentration (else it was considered non-DDI or inconclusive).

Note that overtly cytotoxic concentrations (i.e., viability <40% based on MTT assay at 24 hr) should not be used for classification. Chemicals that yield a DDI call at any concentration (meeting the viability criteria) will be classified as DDI.

Overall, any DDI call in the three-pronged approach would lead to a DDI call. If an ‘inconclusive’call is made in any of the three classifications, the overall call for the chemical would be based on the majority (e.g., one inconclusive’in PCA, but non-DDI for HCA and PA = non-DDI). If all three calls are ‘inconclusive’, the chemical cannot be classified using the TGx-DDI biomarker.

Section 7. Statistical Analysis

Each laboratory used the 'RCC data' file produced by the NanoString Instrument and follow the NanoString workflow with the nSolver Analysis Software. Each laboratory was responsible for conducting their own QC of data within the nSolver software. Data were then be normalized using the nSolver software and the estimated log2 fold changes for each chemical at the three concentrations were saved as a tab-delimited text file (one file per chemical).

Laboratories used the online tool (https://cebs.niehs.nih.gov/tgxddi/tool) using the Batch Data option in the microarray platform drop-down menu, which performs the classification for a single chemical with the three concentrations. The software tool only requires the user to enter the test chemical name and the file name to be uploaded to the tool. By clicking "Submit" the online tool conducted all the necessary analysis for the TGx-DDI biomarker. Using the data interpretation procedure outlined in **Section 5**, the laboratory applied the three-pronged approach for each concentration (details are explained in the following section). A chemical was classified as DDI positive if any of the three concentrations are classified as DDI. A chemical was classified as DDI negative if for all concentrations the three-pronged approach gave a non-DDI classification.

Any DDI call in the three-pronged approach lead to a DDI call. For samples without a definitive DDI classification, a majority rule was used to classify them as non-DDI or inconclusive.

For each laboratory, the Cooper statistics (accuracy, balanced accuracy, sensitivity, specificity) were estimated (see below).

Definitions of the Cooper Statistics

	**Truth Standard**	
**TGx-DDI**	**DDI**	**Non-DDI**
**DDI Call**	*A*	*B*
**Non-DDI Call**	*C*	*D*

$$Sensitivity = \frac{A}{A + C}$$

$$Balance\ Accuracy = \frac{Sensitivity + Specificity}{2}$$

$$Specificity = \frac{D}{D + B}$$

$$Accuracy = \frac{A + D}{A + B + C + D}$$

Three-Pronged Approach

The three-pronged approach is comprised of the nearest shrunken centroids probability analysis (PA), principal component analysis (PCA), and hierarchical cluster analysis (HCA).

Nearest Shrunken Centroids Probability Analysis

The probability analysis (PA) uses the class probabilities and discriminant functions outlined in Tibshirani et al. (2002). A detailed, documented step-by-step approach applying the Tibshirani et al. (2002) methodology to obtain the nearest shrunken centroids along with the accompanying R Code and data are presented in the Data Brief article by Williams et al. (2015). Given the shrunken centroids, the probability for a new test agent, x*, was estimated using the discriminant scores for the DNA damage-inducing (DDI) and non-DDI nearest shrunken centroid, x^k. The discriminant score is written as follows:

$$\delta_k(x^*) = \sum_{i=1}^{64} \frac{\left(x_i^* - x_i^k\right)}{(s_i - s_o)^2} - 2log(0.5)$$

where s_0 is a positive constant set to be the median of the within-class standard deviations, s_i, for all genes in the biomarker. The shrunken centroid coefficients, x_i^k, are presented in Table 1 of Williams et al. (2015) with the estimated standard deviations. The discriminant scores were used to construct the DDI probability estimate, by analogy to Gaussian linear discriminant analysis:

$$Prob(DDI) = \frac{exp\left(\frac{-1}{2}\delta_{DDI}(x^*)\right)}{exp\left(\frac{-1}{2}\delta_{DDI}(x^*)\right) + exp\left(\frac{-1}{2}\delta_{non-DDI}(x^*)\right)}$$

A positive DDI call was made if the estimated probability was > 90% and a non-DDI call was made if the estimated probability was < 10%; otherwise, the call was 'inconclusive'. The 90% and 10% thresholds were selected and not data derived.

Principal Component Analysis

The PCA call for a new test agent was conducted in the NIESH CEBS classification tool by estimating the first principal component using the loadings determined from the training set. In the PCA, the correlation matrix was used in estimating the principal components. In this analysis, the DDI or non-DDI call were made based on the sign of the estimated principal component. If the first principal component was < 0, a DDI call was given. Similarly, if the value was > 0, a non-DDI call was made. The online tool provided this analysis as a scatterplot of the first and second principal components. A red vertical line at 0 for the first principal component was displayed to aid the user with regard to making the call.

Please see below for the equation of PC1 to be used for PCA analysis of the test compounds.

Gene	Mean	Standard Deviation	PCA Loading	Gene	Mean	Standard Deviation	PCA Loading
ACTA2	0.086	0.131	-0.093	HIST1H3D	0.0269	0.1551	0.0913
AEN	0.071	0.207	-0.152	ID2	0.0641	0.1926	0.1109
ARRDC4	0.111	0.196	-0.129	IKBIP	0.0513	0.1558	-0.1383
B3GNT2	-0.006	0.188	0.113	ITPKC	0.0757	0.1007	-0.1347
BLOC1S2	0.107	0.170	-0.154	ITPR1	0.0060	0.1722	0.1048
BRMS1L	0.078	0.136	-0.125	LCE1E	0.1403	0.2682	-0.1321
BTG2	0.187	0.189	-0.140	LRRFIP2	0.0265	0.1281	0.1123
C12orf5	0.119	0.172	-0.140	MDM2	0.1470	0.1780	-0.1422
CBLB	-0.022	0.125	0.129	MEX3B	0.1100	0.1435	-0.0932
CCP110	0.064	0.117	-0.152	NLRX1	-0.0273	0.1227	-0.1214
CDKN1A	0.247	0.203	-0.121	PCDH8	0.1902	0.2420	-0.1437
CEBPD	-0.113	0.242	-0.101	PHLDA3	0.1160	0.2117	-0.1601
CENPE	-0.059	0.137	0.110	PLK3	0.0641	0.2312	-0.1126
COIL	-0.086	0.179	-0.107	PPM1D	0.1801	0.1959	-0.1510
DAAM1	0.085	0.189	0.119	PRKAB1	0.1538	0.2269	-0.1583
DCP1B	0.066	0.123	-0.153	PRKAB2	0.0737	0.1283	-0.1331
DDB2	0.114	0.155	-0.157	PTGER4	0.0523	0.1954	0.1185
DUSP14	0.099	0.107	-0.131	RAPGEF2	0.0214	0.1611	0.1261
E2F7	0.100	0.172	-0.151	RBM12B	-0.0296	0.1165	-0.1323
E2F8	-0.055	0.202	-0.128	RPS27L	0.0897	0.1147	-0.1330
EI24	0.021	0.103	-0.151	RRM2B	0.1712	0.2116	-0.1290
FAM123B	-0.120	0.168	-0.139	SEL1L	0.0353	0.1360	0.0816
FBXO22	0.045	0.111	-0.147	SEMG2	0.0719	0.1428	-0.1118
GADD45A	0.261	0.202	-0.111	SERTAD1	0.2469	0.2590	-0.1260
GXYLT1	0.045	0.068	-0.117	SMAD5	0.0580	0.1283	-0.1337
HIST1H1E	-0.128	0.295	0.081	TM7SF3	0.0535	0.1115	-0.1444
HIST1H2BB	0.014	0.226	0.076	TNFRSF17	-0.0879	0.2837	-0.1101
HIST1H2BC	0.009	0.245	0.087	TOPORS	-0.0478	0.1523	-0.1181
HIST1H2BG	0.029	0.279	0.085	TP53I3	0.0637	0.1498	-0.1483
HIST1H2BI	0.011	0.236	0.076	TRIAP1	0.0421	0.2183	-0.1482
HIST1H2BM	-0.002	0.263	0.075	TRIM22	0.0865	0.2124	-0.1517
HIST1H2BN	0.004	0.118	0.081	USP41	0.0346	0.0876	-0.0529

$$PC1 = \sum_{Gene\,=\,1}^{64} PC1\ Loading_{Gene} \frac{(log2FC_{gene} - Mean_{gene})}{Std.Dev_{Gene}}$$

38

Hierarchical Cluster Analysis

The inconclusive chemical could either cluster with the DDI agents (DDI call) or non- DDI (non-DDI call) agents or cluster on its own as a singleton cluster (inconclusive).

Any positive call (probability > 0.9 of DDI by probability analysis *OR* clusters with DDI agents by HCA *OR* has a negative PC1 in the PCA) lead to a positive DDI call. If the classification was not clear, then the inconclusive chemical may be a borderline case or there may be an outlier for at least one of the genes in the biomarker. If the classification was not a clear DDI or non-DDI call, then the experiment was repeated.

These tools do not provide confidence intervals. We made DDI or non-DDI calls only. However, we had a high degree of confidence in the non-DDI calls under those biological conditions. Probability analysis derived an estimated probability of being non-DDI (< 10% probability of being DDI) or DDI (> 90%). Anything in between was inconclusive. Anything inconclusive will warrant further scrutiny. Classification would then be based on PCA and HCA.

References

Tibshirani R, Hastie T, Narasimhan B, and Chu G. Diagnosis of multiple cancer types by shrunken centroids of gene expression. Proc Natl Acad Sci U S A. 2002;99:6567–6572.

Williams A, Buick JK, Moffat I, Swartz CD, Recio L, Hyduke DR, Li HH, Fornace AJ, Jr., Aubrecht J, and Yauk CL. A predictive toxicogenomics signature to classify genotoxic versus non-genotoxic chemicals in human TK6 cells. Data Brief. 2015;5:77–83.

Appendix I. QA/QC instructions

Samples that should be excluded *before* beginning nSolver analysis:
QC flags may occur due to poor quality samples. Therefore, if the RNA integrity number (RIN) is < 7 the sample should not be sent for nanoString analysis (or, if already analyzed, the RCC file should be excluded).

Note: QC flags may also occur at high concentrations because of cytotoxicity and inhibition of transcription. The MTT threshold of 40% viability should be scrutinized/applied, and overly cytotoxic samples should be excluded from nSolver analysis.

Basic QC flag examination
For each compound's experiment, evaluate the following sections for the QC flags indicated below. We recommend excluding samples from further nSolver analysis if any of the following conditions associated with QC flag(s) occur. See workflow below for how to proceed with removing samples with flags. For a detailed description of QC flags and troubleshooting considerations refer to the "Supplementary Information" at the end of the document.

Remove samples with the following QC flags:
In the experiment 'Raw data' tab

1. A 'binding density QC flag' with a binding density less than 0.07.
2. An 'imaging QC flag' with a value less than 0.75.

In the experiment 'Normalized data' tab

3. An 'mRNA content normalization flag'.

In the experiment 'Ratio data' tab

4. If no flags are present in the other tabs, you should not see any flags in the ratio data tab.
    a. Note: If samples with QC issues are included here, there may be ratio data normalization flags that impede data export. Carefully scrutinize the data to make sure that all samples that had any QC flags described above are removed.

Assess replicates

I. If a sample has to be removed because of one or more of these flags, the analysis can continue with the rest of the samples as long as 2 out of 3 replicates remain from that treatment group.

II. If two or more samples are excluded from a triplicate, the nanoString assay should be re-run if RNAs are in good quality and the concentration is not overtly cytotoxic.

III. The cell culture experiment should be repeated from cell culture if there is not enough RNA left or the RIN does not meet the requirements.

WHEN TO REMOVE THE SAMPLES FROM THE EXPERIMENT – see workflow below

Workflow for analysis of each experimental compound

Note: Samples with low RIN should not have been run and should not be included in the analysis.

Analysis #1 (Individual replicates)

1. Assess QC flags – record all samples with flags that need to be removed
2. Determine if there are sufficient solvent controls (n=2) and experimental samples to proceed with the analysis (described above I – III) for each compound.
3. If proceeding, edit the experiment in nSolver (snapshot below). Leaves out the RCC files for the flagged samples by selecting the sample that needed to be removed in the page “Add Samples/Lanes”, and clicking “Exclude Selected”.

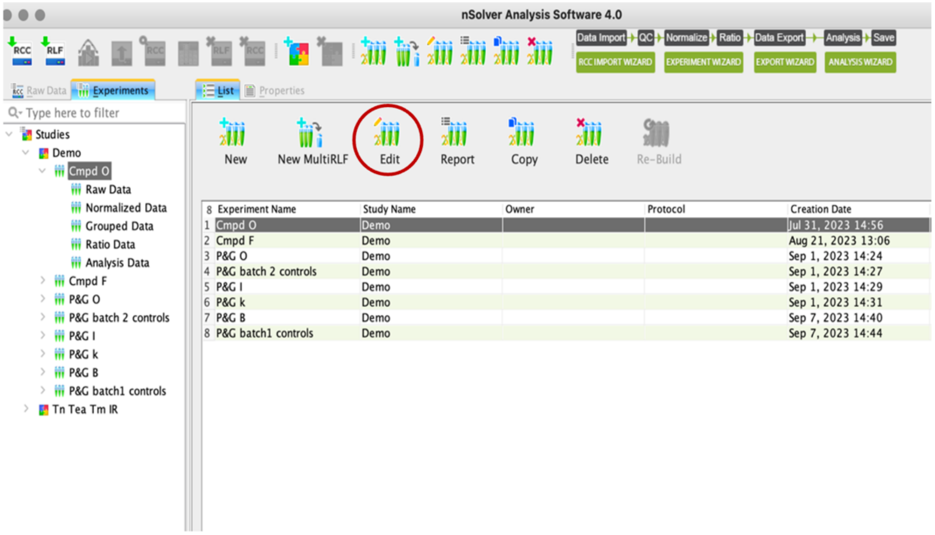

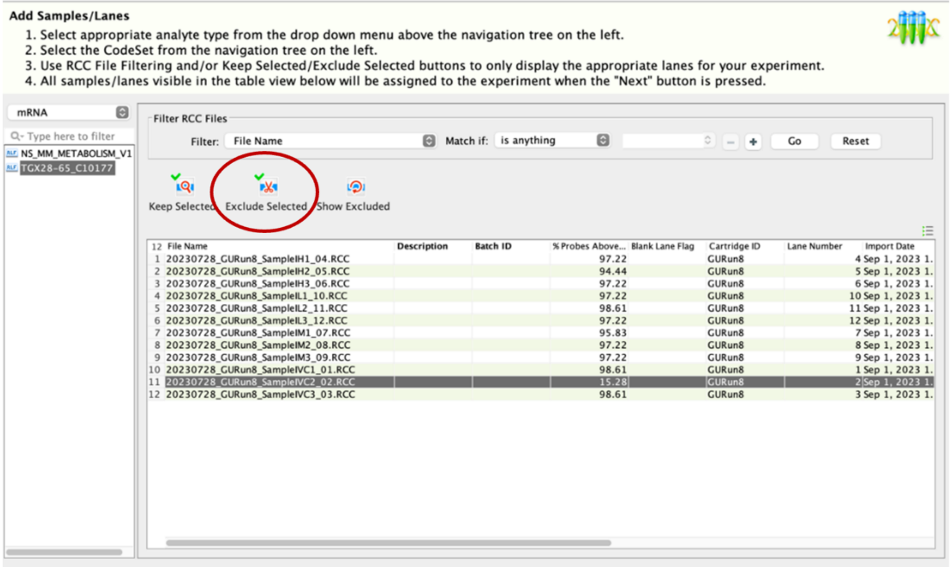

4. Rerun the nSolver analysis.

Analysis #2 (Grouped analysis)

Perform the Grouped analysis as described in the SOP, excluding the same RCC files as in the individual replicate analysis.

Further detailed instructions provided below.

Raw data tab flags

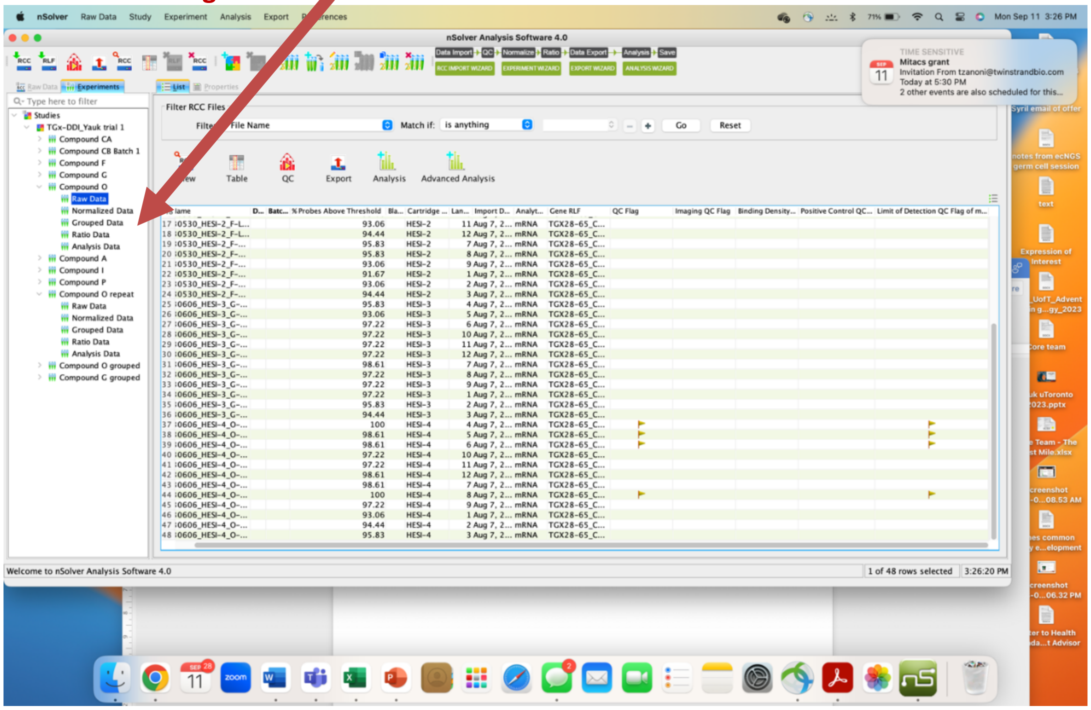

The following QC flags can be found on this tab:

- Imaging QC
- Density binding
- Positive control QC
- Limit of detection of QC flag mRNA

Note: These specific values can be found right clicking the table header in Raw Data and selecting “show all hidden columns”

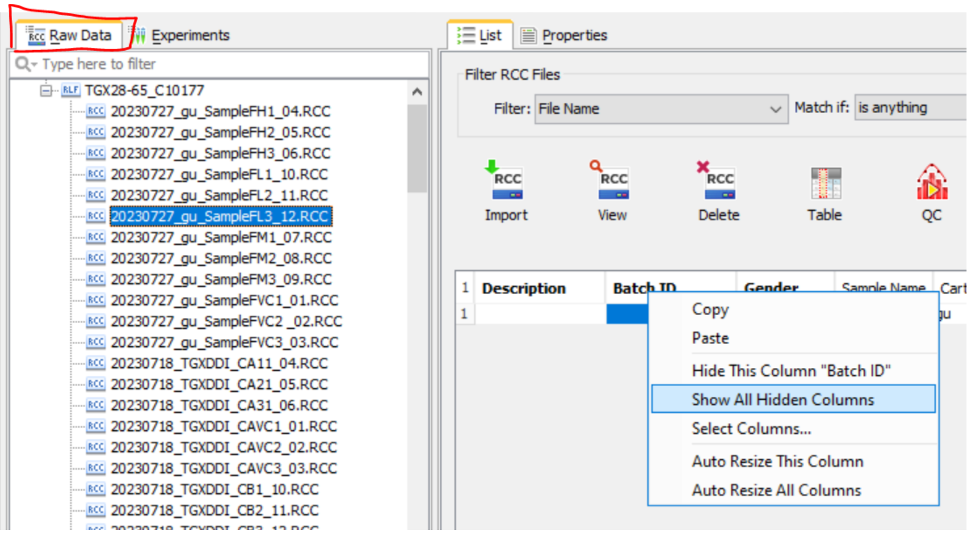

Instructions: Remove samples with Imaging QC or Density binding QC flags using the cutoffs mentioned above.

Normalized data tab flags

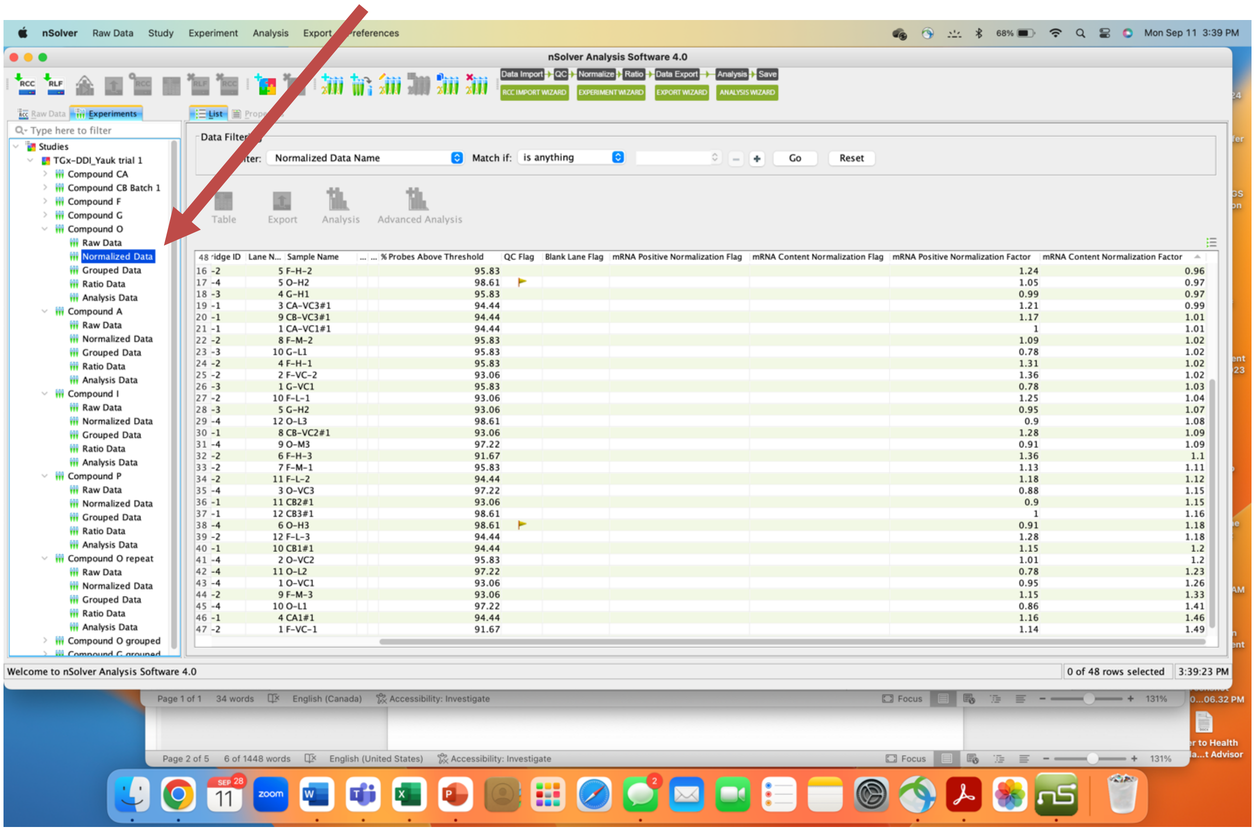

The following QC flags can be found on this tab:

mRNA positive normalization

mRNA content normalization

mRNA positive normalization factors

mRNA content normalization factors.

Instructions: ***Remove samples with mRNA content normalization flag.***

Grouped data tab flags

IGNORE THESE QC FLAGS

Ratio data tab flags

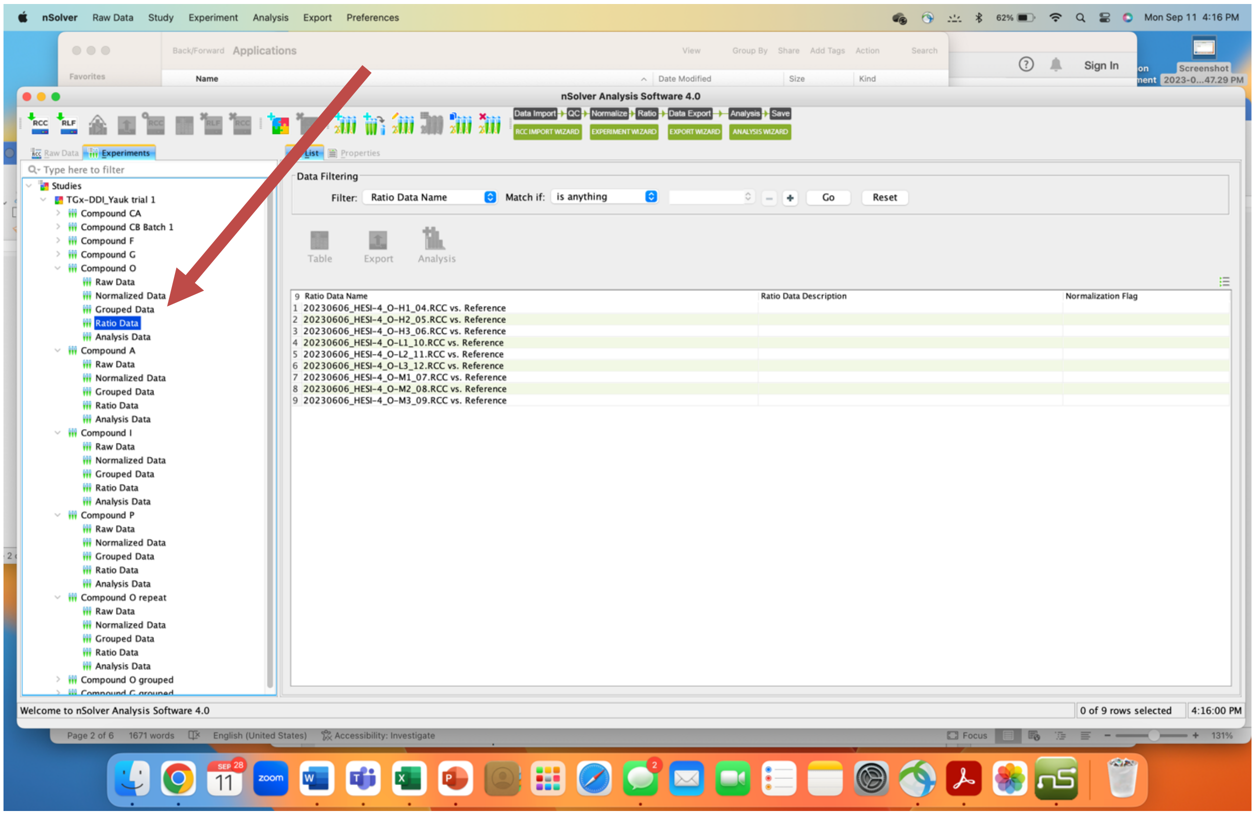

The following QC flags can be found on this tab:

Normalization flag

Instructions:

If samples with QC issues are included here, there may be ratio data normalization flags that impede data export. Carefully scrutinize to make sure that all samples that had QC flags described above are removed.

Supplementary Information

Below we provide some additional information on the QC flags used for eliminating samples in the TGx-DDI workflow.

QC flags in raw data
There are two quality control parameters common to all nCounter assays (Binding Density QC and Imaging QC) in the Raw Data QC that are relevant to this ring trial. These QC filters enable the identification of outliers that should be removed from further analysis.

Binding Density QC
This metric is a measurement (in spots per square micron) of the concentration of barcodes seen by the instrument. The Digital Analyzer may not be able to distinguish each probe from the others if too many are present. The ideal range 0.1 - 2.25 spots per square micron has been established for assays run on an nCounter *MAX* or *FLEX* system and 0.1 - 1.8 spots per square micron on the nCounter *SPRINT* system.

Measurements outside of these ideal ranges will be flagged, but should be checked to see how much they deviate from the ideal range. If they are *only slightly outside of range, they do not indicate a problem* in the data and can be bypassed. If they deviate a great deal, troubleshooting should continue since this may indicate reduced resolution.

Troubleshooting a Binding Density Flag

- **Check the input amount.** More sample input will result in an increased Binding Density.
- **Consider the expression level** of the targets in the CodeSet. If the targets in the CodeSet are highly expressed, Binding Density will go up simply because more mRNA molecules are being targeted in your samples.
- **Consider the size of the CodeSet**. If a CodeSet contains probes for more targets, then Binding Density will usually be higher.

Imaging QC
This metric reports the percentage of fields of view (FOVs) the Digital Analyzer or Sprint was able to capture. At least 75% of FOVs should be successfully counted to obtain robust data. A flag in this area may indicate something as simple as a crooked or smeared cartridge, which can be remedied by rescanning (MAX or FLEX systems only; ideally within one week). Consistently reduced percentages, however, can be indicative of an issue associated with the instrumentation.

Troubleshooting an Imaging Flag

- **Check alignment of the cartridge** in the instrument – if crooked, reload and rescan.
- **Check the bottom of the cartridge** - clean with 70% EtOH and a lint-free wipe, reload, and rescan.

We will also set our own filter on % probes passing threshold using criteria X. Per the nSolver User Manual, the % Probes Above Threshold column is useful for a second check on the overall quality of the data; a value that deviates a great deal from the values of the other samples may indicate a QC issue.

Positive Control QC Parameters

Positive controls assess three general QC purposes:

- Overall assay efficiency. nSolver raises a warning flag when the geometric mean of positive controls is more than three-fold different from the geometric mean of all samples.
- Assay linearity. Decreasing linear counts are expected from POS_A to POS_E (POS_F is considered below the limit of detection).
- Limit of detection (LOD). It is expected that counts for POS_E will be higher than background, which is represented by the mean of the negative controls plus two standard deviations (for most assays) or simply the mean of the negative controls (for miRNA assays).

Positive Control Linearity QC

This metric performs a correlation analysis in log2 space between the known concentrations of positive control target molecules added by NanoString and the resulting counts. Correlation values lower than 0.95 may indicate an issue with the hybridization reaction and/or assay performance.

Troubleshooting a Positive Control Linearity Flag

If all the following are true, it is safe to include the flagged sample:

- r-squared values are ~ 0.90
- only a single POS control is abnormal
- the raw counts for the sample appear normal

If one or more of the above is *not* true, contact support@nanostring.com

Positive Control Limit of Detection QC
This measures the limit of detection of the assay by comparing the results from the positive control probes and those from the negative control probes. Specifically, it is expected that the 0.5 fM positive control probe (*Pos_E*) will produce raw counts at least two standard deviations higher than the mean of the negative control probes. You can modify the number of standard deviations used to estimate significance. In the event of a flag, see the *Troubleshooting* box, here, and the *What to Do If You Have a Flag* section.

Troubleshooting a Positive Control Limit of Detection Flag

Too high background may be due to:

- Mixing of reporter and capture probes in hybridization buffer master mix.
- Too much time elapsed between adding capture probe and loading in thermal cycler.
- High counts in one of the negative controls – NEG control could be elevated due to cross hybridization with targets in the sample.

Too low POS counts may be due to:

- Sub-optimal hybridization – check thermal cycler temperature and consider whether sample impurities (chaotropic salts, for example) may have been introduced.

What to Do If You Have a Flag
QC flags presented in your data imply that closer examination of the affected lane is warranted prior to proceeding with analysis. Oftentimes, flags are raised due to intended attributes of a particular CodeSet or sample. QC flags do NOT necessarily mean that data is unreliable.

To determine whether a QC flag is indicating a critical problem, examine the raw and normalized data (normalized data is generated while making an Experiment; see the *Creating Experiments* section). Specifically, check low count transcripts to determine if flagged samples have a poorer limit of detection than non-flagged samples. There are several ways to do this:

- A simple visual scan of the data may suffice to detect problems in the flagged samples. This can be performed on raw data which have been background subtracted in nSolver to identify targets that are below the background.

- Review the results from the positive and negative controls. Positive controls with low counts or negative controls with counts significantly above background can trigger flags and should be checked to see if they indicate more serious issues with the data.

- Outlier samples can be identified by generating a heat map of normalized data from all samples to see if the flagged samples in question are strongly divergent from other samples with similar pathology. You will need to proceed through the steps to create an Experiment before doing an Analysis, at which point you can refer to the *Agglomerative Cluster (Heat Map)* section.

- You can examine the calculated QC metrics. Right-click or command-click on one of the table column headers in the raw data table or use the column options icon to *Show all Hidden Columns*. This will reveal a column next to each QC flag column containing the numbers on

which the QC check was based. If these QC metrics only deviate from the threshold by a very small margin (i.e., the FOV registration is 74% instead of 75%), then the resultant data may be quite robust and usable. To review the parameter settings, select the QC button above the table.

In addition to these general guidelines, refer to the troubleshooting boxes in the individual *QC Parameters* sections, above, for more troubleshooting ideas on individual QC parameter flags. Normalization QC flags are addressed in the *Normalization* section.

In cases where there are no QC flags, it is still advised that you examine the calculated QC metrics and check whether there are outliers. For example, if the Imaging QC for 11 out of 12 samples is 95% or higher and one sample presents a value of only 76%, this would not trigger a QC flag. However, it is still important to investigate why this one sample may have had a significantly lower imaging quality score.

QC flags in normalized data

A QC flag for content normalization indicates that the flagged sample had a content (or housekeeping gene) normalization factor more than 10-fold different from the average sample in the same experiment. Review your Normalized Data Table (see the *Normalized Data Table* section) to see any Content Normalization flags your data may have.

Content Normalization flags can be caused by:

- A significant reduction in overall assay efficiency for that sample.
- An effective reduction in quantity or quality (fragmentation) of the input analyte. The likelihood of a reduction in assay efficiency can be assessed by the presence of any other QC flags for that sample.
- Insufficient RNA targets to count. If the sample has no other QC flags except that for Content Normalization, this indicates that the assay is working well, but contains low RNA concentrations or highly fragmented RNA (such as from an archival FFPE sample).

Considerations for what to do if you have a Normalization QC Flag:

- If you see a normalization flag for the POS controls, you may have had an assay-level problem.
- The biggest effect of any QC flag will be on low-expression targets.
- If the sample failed the QC by a wide margin, you may want to consider dropping the sample.
- If the normalization factor was only slightly outside the recommended range or if the genes being studied were moderately to highly expressed, you may consider overlooking the flag and keeping the sample for analysis.

48

Appendix II. TGx-DDI data analysis protocol using nSolver and NIEHS/CEBS TGx-DDI web tool

A. Perform the analysis on individual assays

1. Open nSolver 4.0. Click 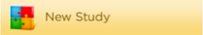 to create a new study.
   → nSolver 4.0 is available at https://nanostring.com/products/analysis-solutions/ncounter-analysis-solutions/. The 'Optional Advanced Analysis software' is not needed.

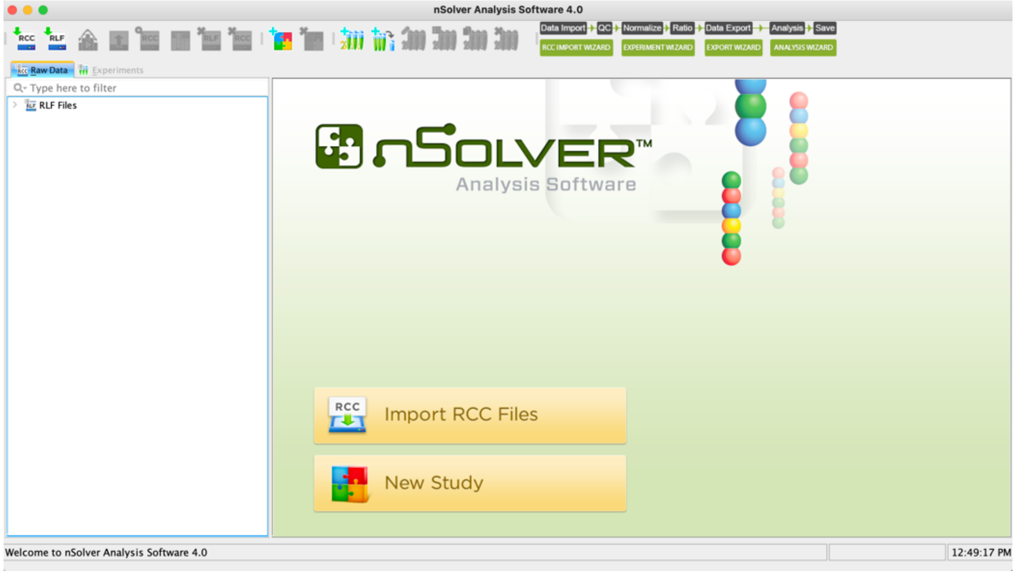

2. Click 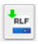 to import RLF file (TGX28-65_C10177.rlf)

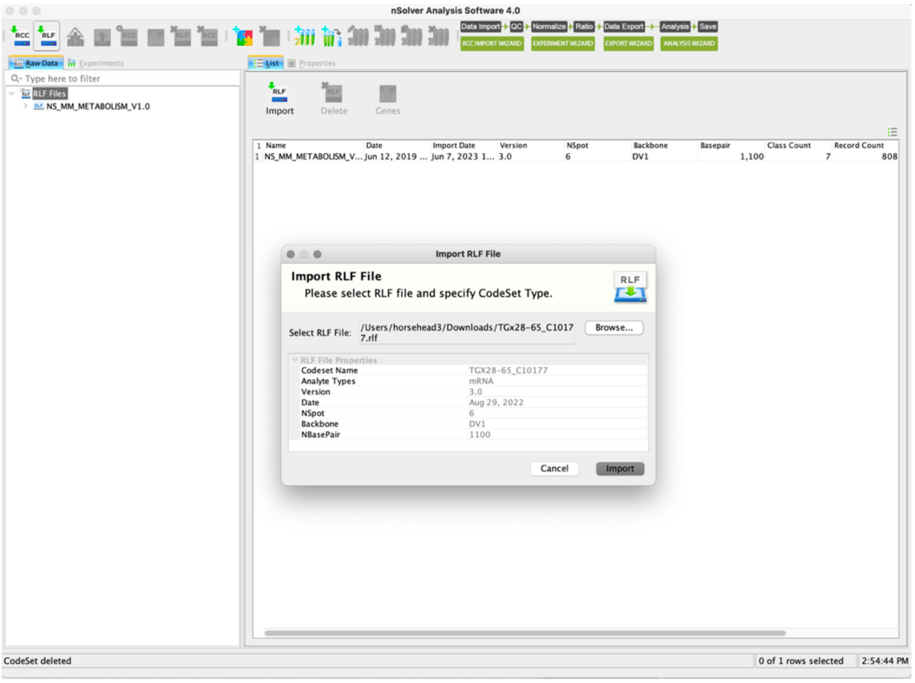

3. Import RCC files.

→ This may be done one RCC file at a time, or you may import the entire dataset all at once.

3.1. Navigate to the RLF (i.e. TGX28-65_C10177) for your dataset from the left panel and select. From the right panel, click 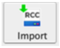 to import the RCC files and click "Next".

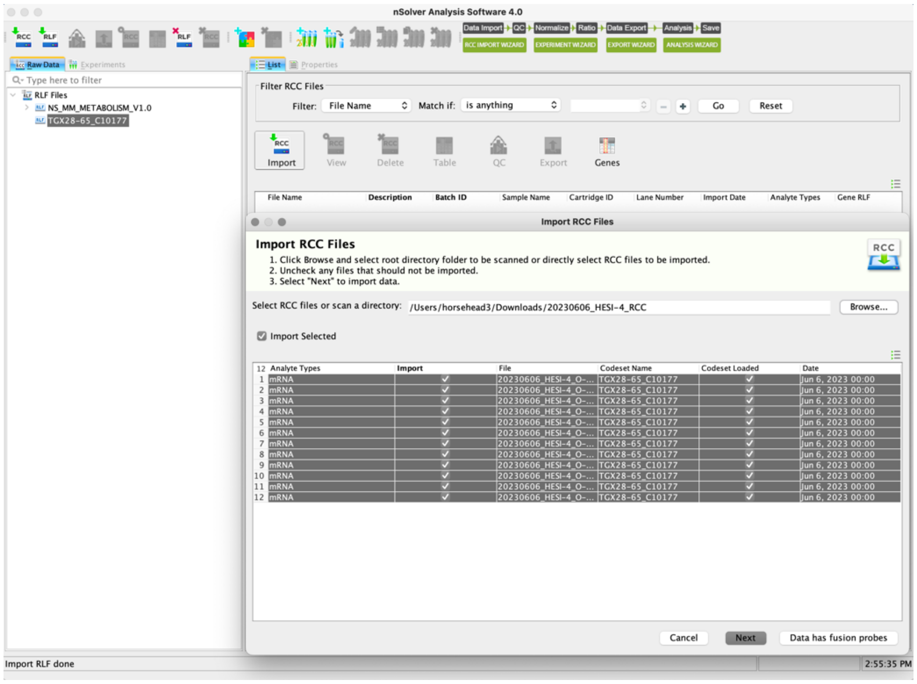

3.2. Run the QC analysis using the default settings shown below. Click 'Import' or 'Run QC' (options differ slightly between the Mac and PC versions of the software).

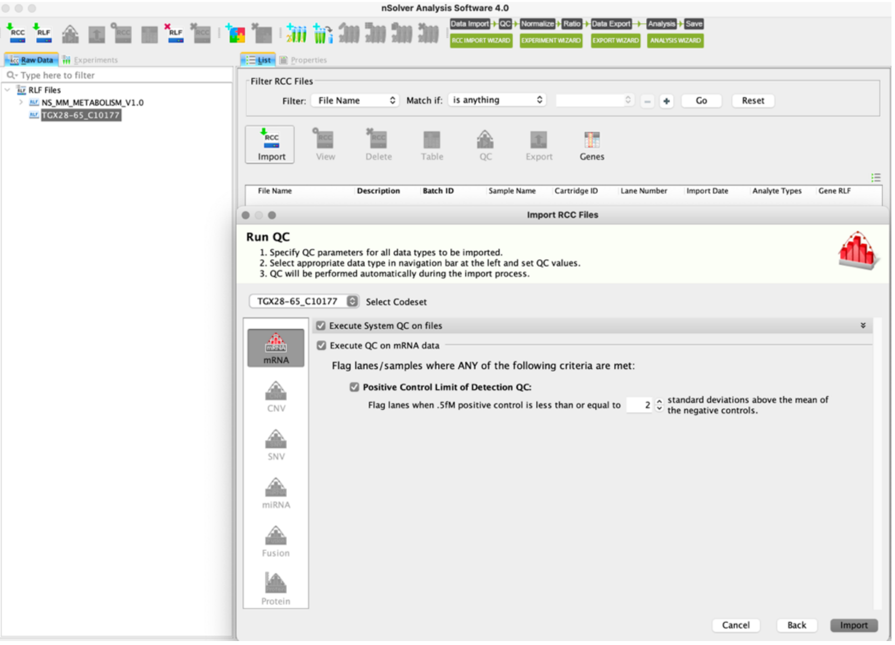

4. Create a new experiment (see how in point 4.1) <u>for each compound</u> (i.e., the data from one RCC file). Also create a separate experiment for each negative or positive control (i.e. individual experiments for batch 1 CA, batch 2 CA, batch 3 CA, batch 1 CB, batch 2 CB, batch 3 CB, and batch 4 CC, respectively). You will have 21 experiments in total.

    4.1 To create a new experiment, navigate to the left panel and select "Experiments" tab. Select the current study. Click 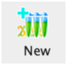

from the right panel to create a new Experiment. Type in the name and click "Next".

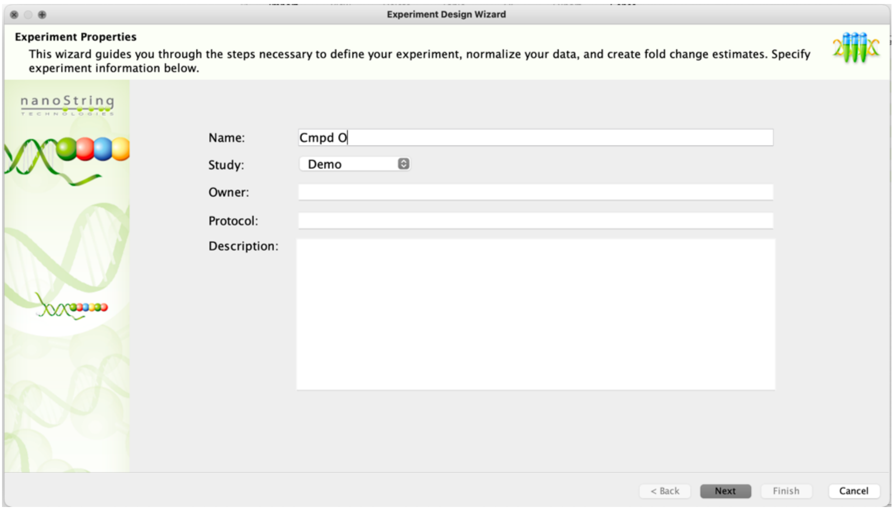

4.2 Select the RLF file from the left panel. From the right panel, select all RCC files that are analyzed in this experiment. When selecting the files for inclusion in the experiment, scroll to the far right of the spreadsheet and note any QA/QC flags. Then click “Next”.

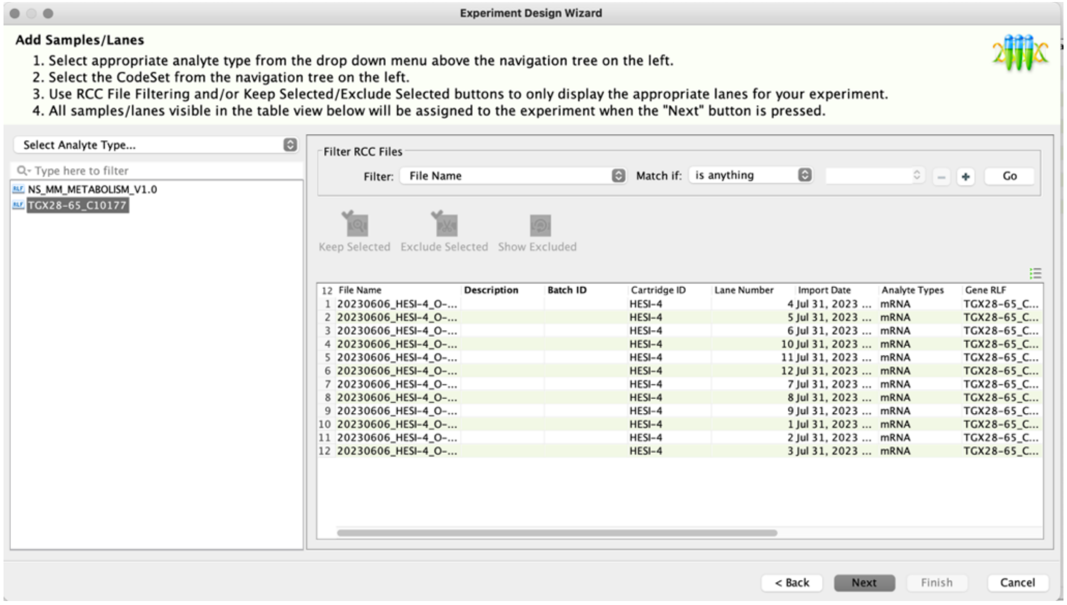

4.3 Click “Next” to skip the ‘Add Sample Annotation’ step.

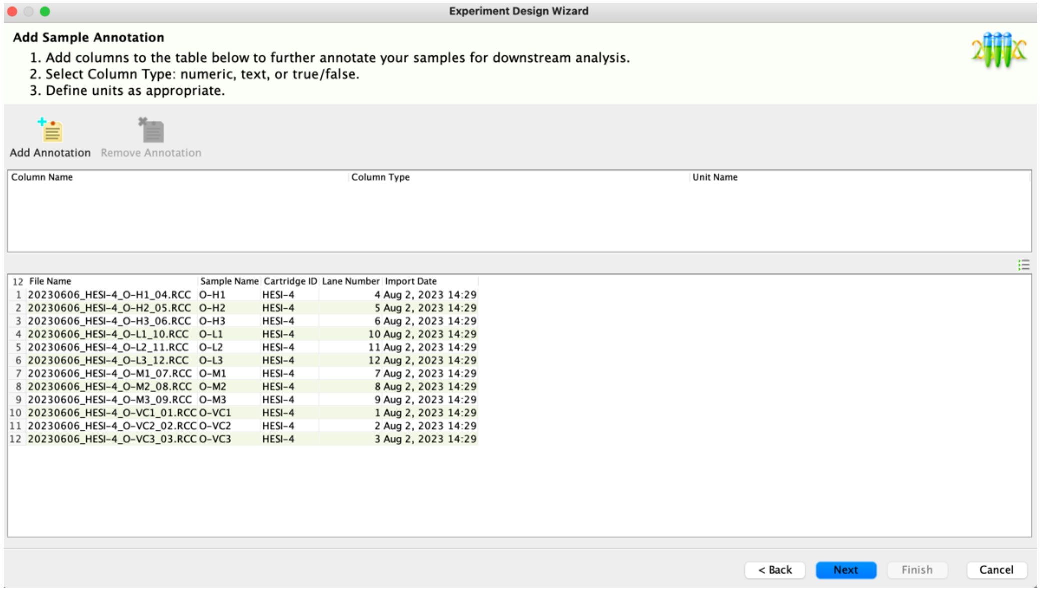

4.4 Check the box for Background Subtraction/Thresholding and click “Next”. Check that the other default parameters match the ones in the figure below.

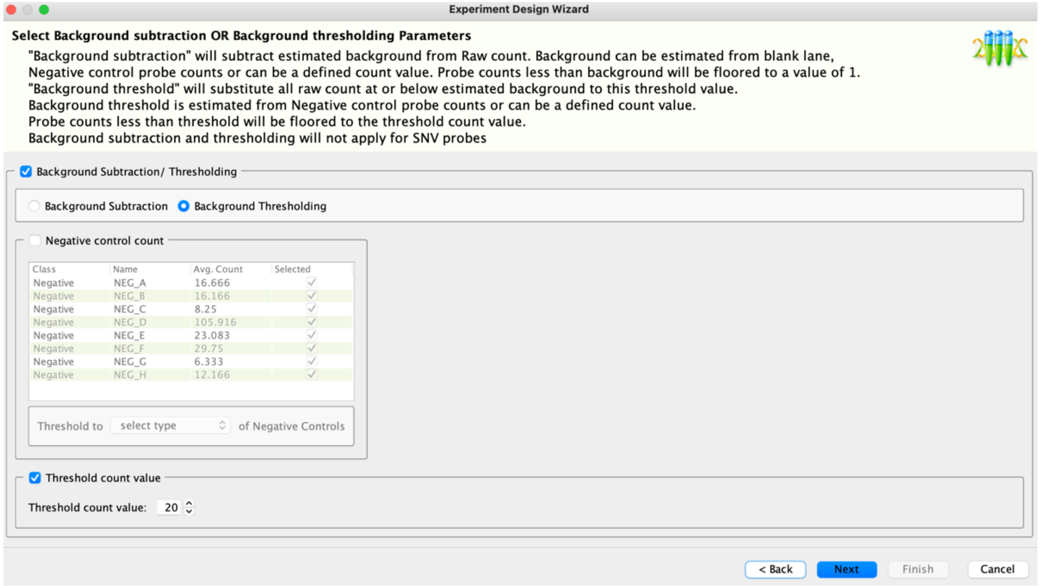

4.5 Use the default normalization settings as shown below. Review to ensure they match the settings below and click “Next”.

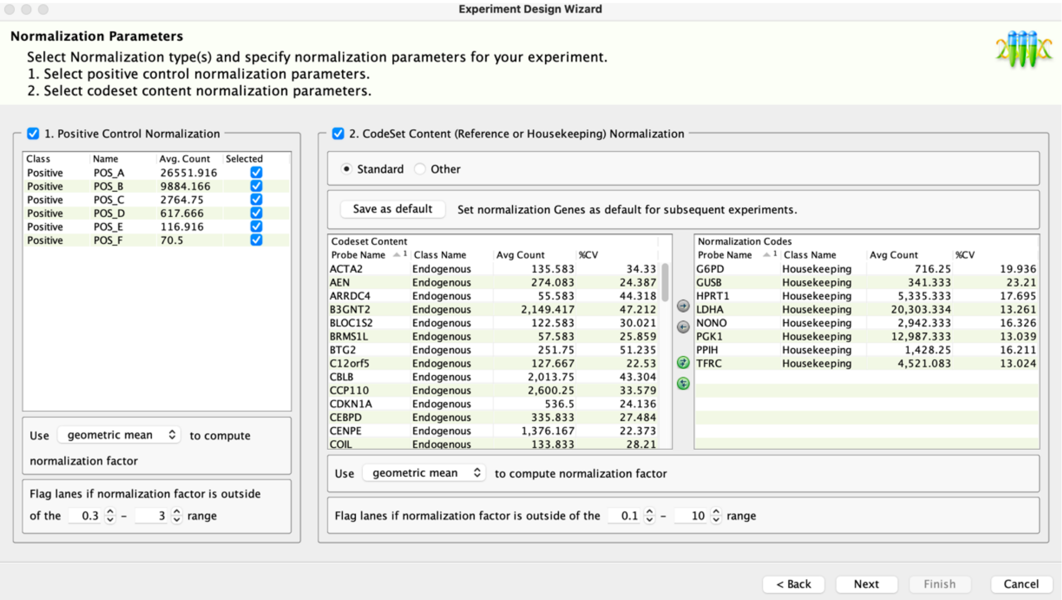

4.6 Calculate fold change. Check the box “Using user selected reference samples”. Move the Vehicle Control Samples (i.e. O-VC1, O-VC2, O-VC3) to the right as Base Samples and click “Next”.

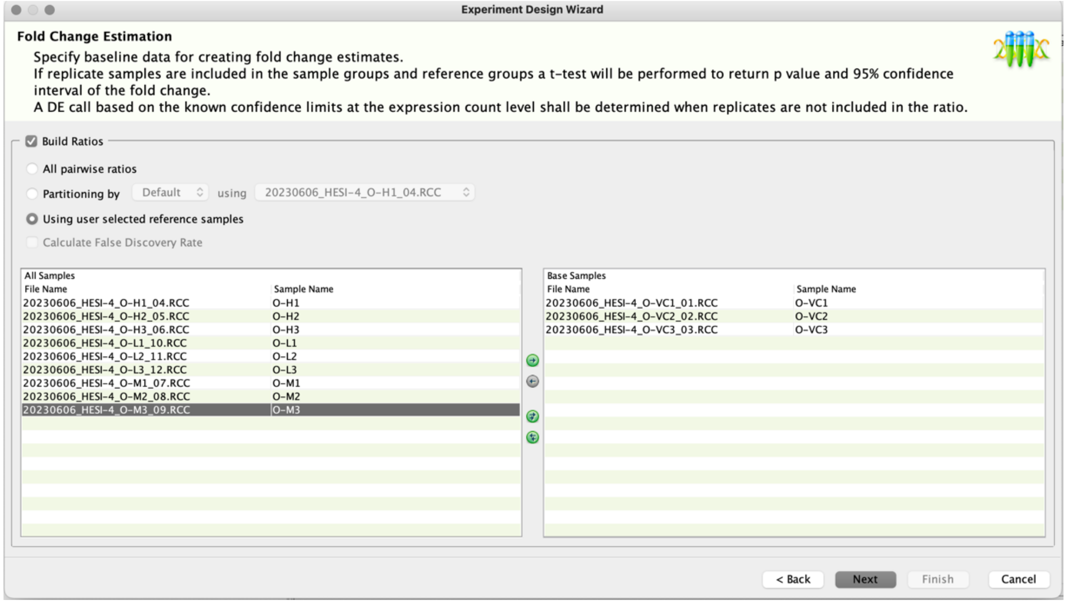

On the prompted window, uncheck the three ratio data names of vehicle control samples (for example, #10, 11, 12 in the screenshot below), and click "Finish".

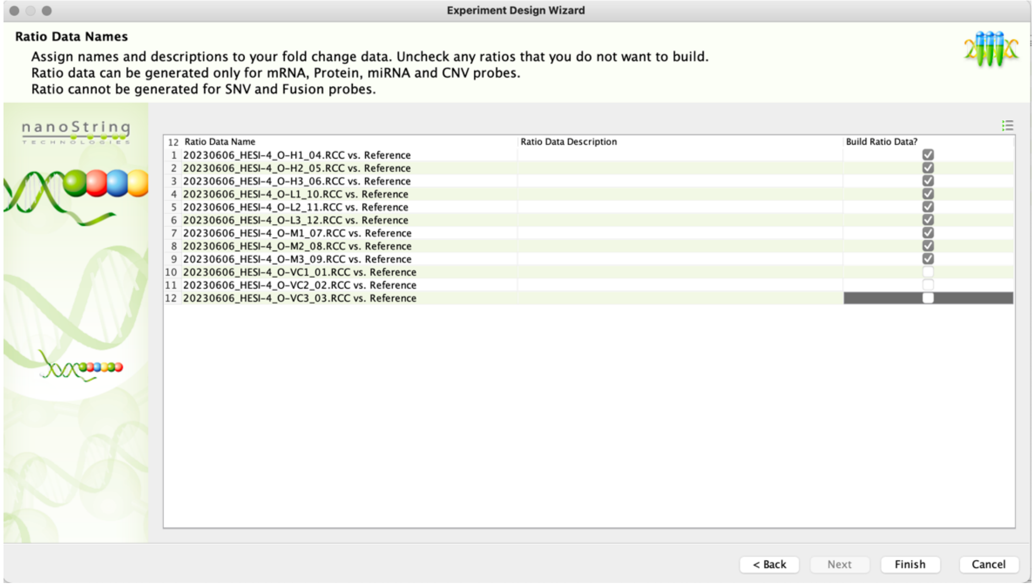

5. Export the log_2Ratio data.

5.1 Navigate the left panel and select "Experiments" Tab. Click on the Studies to expand. Click on to the current study and select "Ratio Data" to display. The "Ratio Data Name" will then be listed on the right panel and all relevant files will be shown.

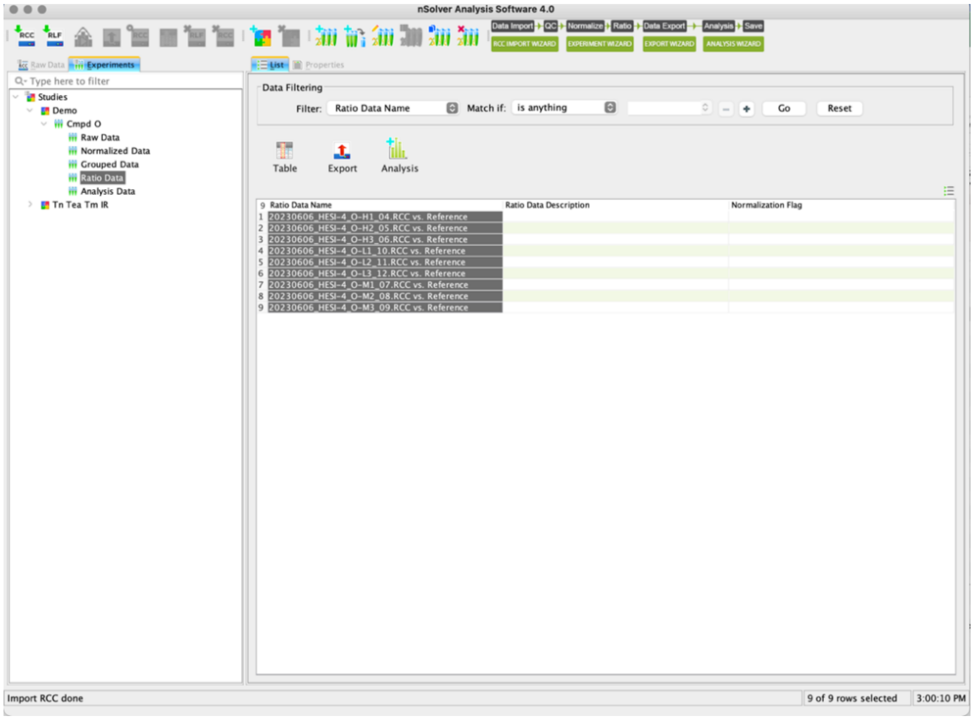

5.2 Select all listed data and click 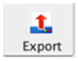 to export. Choose DEFAULT for Locale settings and TAB for Delimiter settings and then click "OK". This window may not appear in some versions of the software. In this case, proceed to step 5.3.

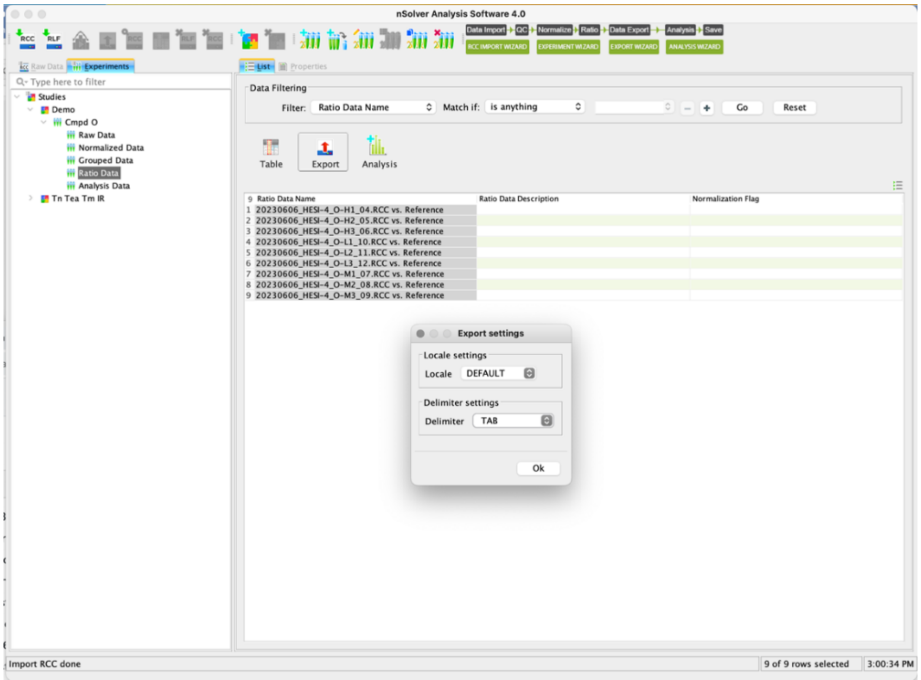

5.3 Select "Custom Text Format Export". Check the box for "Exclude lanes/samples flagged in normalization" and click "Next".

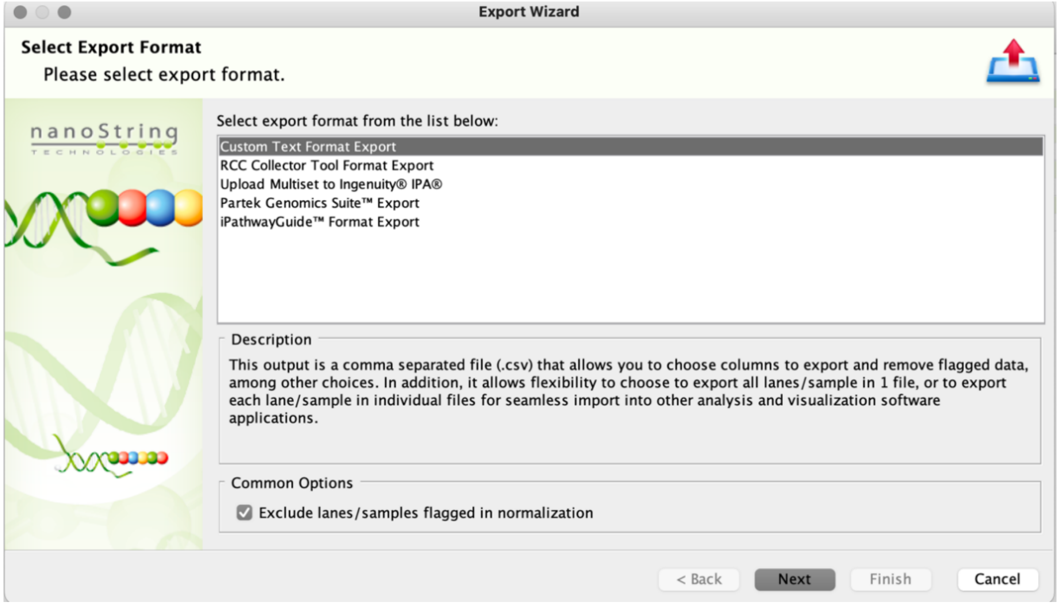

Keep the “Probe Name” and leave the selected data checked (compound x vs reference data). Choose “Tab Delimited” for Format Options. Select Log2 Ratio for Ratio Options and then click “Finish”.

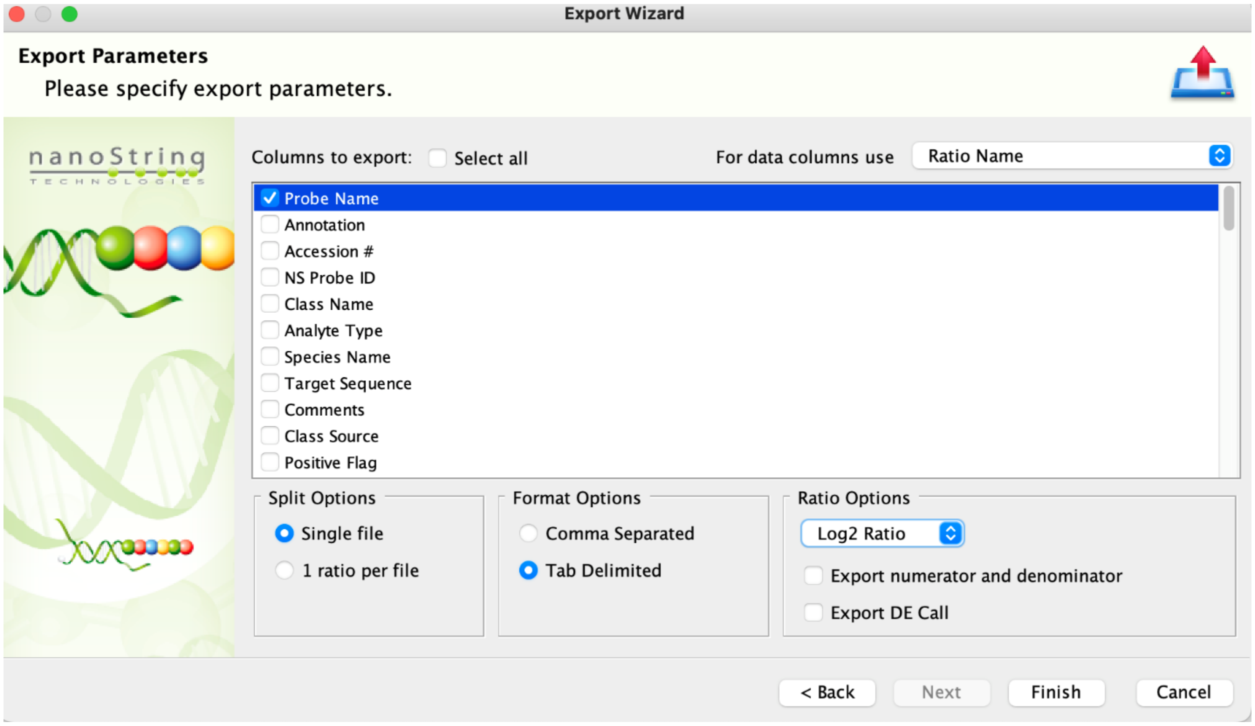

5.4 Name and save the Export Data as Text files (*.txt).

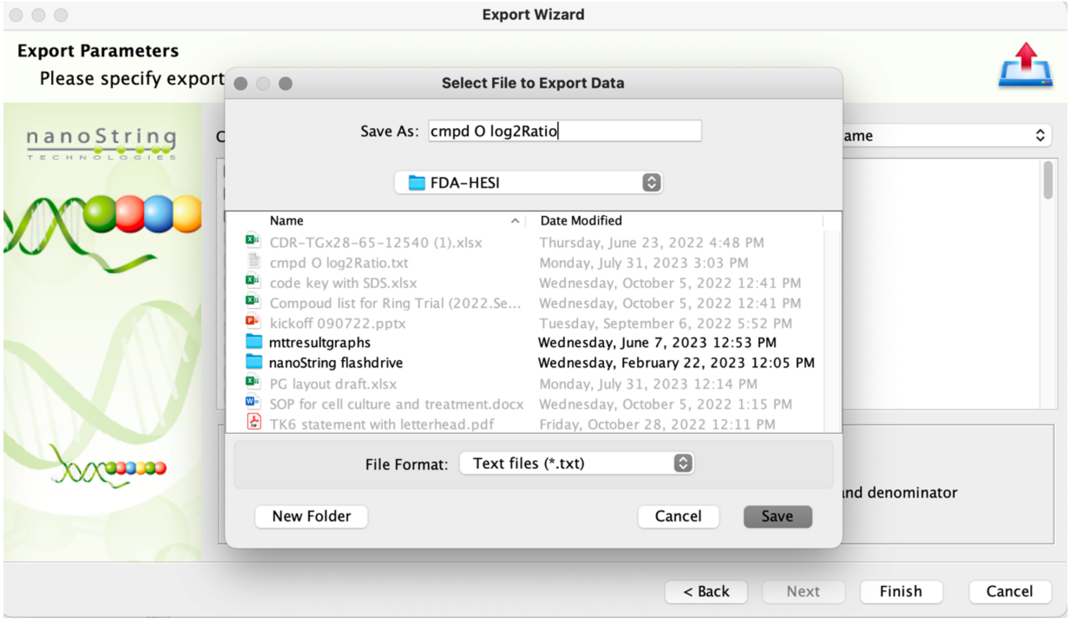

6. Prepare the data file for the TGx-DDI analysis using the NIEHS CEBS tool.

   6.1 Open the downloaded txt file in Excel, rename the ratio data name in the title line. The new name comprises of the compound code (for example “O”), the concentration (“H”, “M”, or “L”), followed by a hyphen (“-”), then (“10 mM”). The 10 mM is added here as a cheating placeholder as the webtool requires to include the concentration and the unit in the title line when a batch analysis is run. The screenshot of an example file after formatting is shown below. It must be formatted exactly as shown for the software to run correctly.

	A	B	C	D	E	F	G	H	I	J
1	Probe Name	OH-10 mM	OH-10 mM	OH-10 mM	OL-10 mM	OL-10 mM	OL-10 mM	OM-10 mM	OM-10 mM	OM-10 mM
2	ACTA2	0.87	0.67	0.91	0.11	0.02	0.11	0.59	0.55	0.62
3	AEN	0.36	0.32	0.26	0.4	0.37	0.35	0.47	0.48	0.39
4	ARRDC4	1.57	1.26	1.71	0.85	0.58	0.84	0.94	0.9	0.98
5	B3GNT2	1.63	1.72	1.78	0.82	0.83	0.96	1.34	1.38	1.27
6	BLOC1S2	0.88	0.54	0.68	0.54	0.35	0.37	0.54	0.59	0.68
7	BRMS1L	0.54	0.49	0.44	0.06	-0.05	0.08	0.25	0.12	0.06
8	BTG2	1.5	1.36	1.37	0.1	0.22	0.13	0.98	1.17	1.05
9	C12orf5	0.03	0.22	0.15	-0.62	-0.29	-0.48	-0.1	-0.06	-0.31
10	CBLB	1.63	1.72	1.76	1.37	1.44	1.52	1.6	1.56	1.54
11	CCP110	0.68	0.76	0.82	1.1	1.16	1.18	0.93	0.92	0.91
12	[illegible]	[illegible]	[illegible]	[illegible]	[illegible]	[illegible]	[illegible]	[illegible]	[illegible]	[illegible]

→ Save the data file as an .xlsx file. The software will not work in any other format.

7. Analysis the data using the NIEHS CEBS tool.

   7.1 Upload the data file on the website (https://cebs.niehs.nih.gov/tgxddi/tool), select “Batch Data” in Microarray platform.

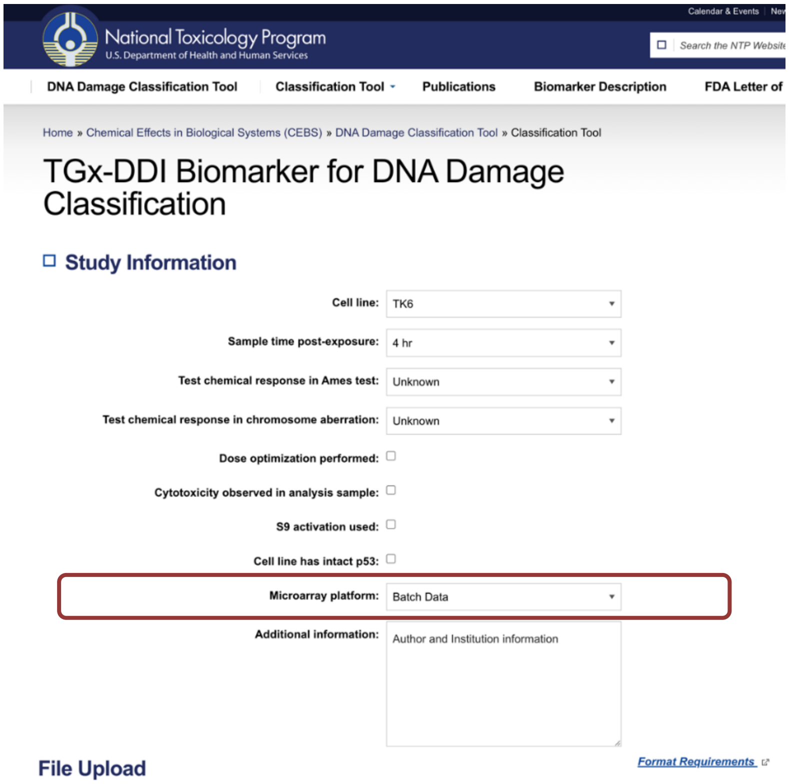

At the lower section of the page, fill in the compound code, and then select the .xlsx data file, then click “SUBMIT”.

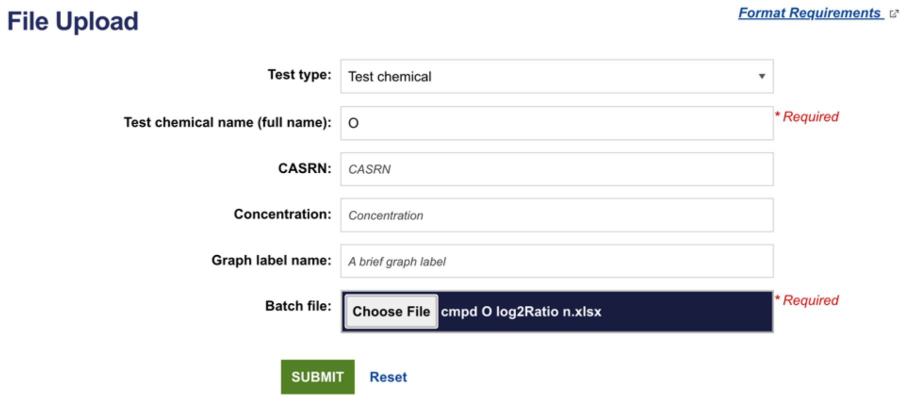

The refreshed window should look like the image below, with content filled in each for the five columns. Click “START PROCESS”.

Home » Chemical Effects in Biological Systems (CEBS) » DNA Damage Classification Tool » Classification Tool » Column Analysis

All Columns Analysis

SHARE THIS:

Here is a list of all the columns based on the uploaded file: **cmpd O log2Ratio n.xlsx**

The graph label, dose, and dose unit extracted from a column name will be used for graphic labels. Please be aware that it may take a significant number of minutes to process all columns of data in the file.

Column Name	Graph Label	Concentration	Concentration Unit	Column Index
Probe.Name				0
OH-10.mM	OH	10	mM	1
OH-10.mM	OH	10	mM	2
OH-10.mM	OH	10	mM	3
OL-10.mM	OL	10	mM	4
OL-10.mM	OL	10	mM	5
OL-10.mM	OL	10	mM	6
OM-10.mM	OM	10	mM	7
OM-10.mM	OM	10	mM	8
OM-10.mM	OM	10	mM	9

START PROCESS ☐ CANCEL

→ The analysis results will show up in a table. Save the summary of results by clicking "DOWNLOAD RESULT TABLE" and save the detailed results for each test case by clicking "DOWNLOAD ALL FILES".

REMOVE	Fold Change<br>Gene Cluster<br>Chemical Cluster	Heatmap ☐ ☐<br>Cluster ☐ ☐<br>Both as PDF	Non DNA Damage Inducing	6.33e-10	0.999999999367087	Test chemical	OM_7	10 mM
REMOVE	Fold Change<br>Gene Cluster<br>Chemical Cluster	Heatmap ☐ ☐<br>Cluster ☐ ☐<br>Both as PDF	Non DNA Damage Inducing	7.99e-12	0.999999999992013	Test chemical	OM_8	10 mM
REMOVE	Fold Change<br>Gene Cluster<br>Chemical Cluster	Heatmap ☐ ☐<br>Cluster ☐ ☐<br>Both as PDF	Non DNA Damage Inducing	3.78e-14	0.999999999999962	Test chemical	OM_9	10 mM

☐ ADD NEW DATA ☐ DOWNLOAD RESULT TABLE ☐ DOWNLOAD ALL FILES ☐ CLEAR ALL RESULTS

B. Examine whether there are inconsistencies in the triplicates

Go through the summary tables of TGx-DDI results. Mark individual assays that yield an opposite call from the other two of the triplicates within that concentration. Check the QC flags of these selected files on the RCC Files page. Write down the RCC file name of those having one or more flags and giving an inconsistent call. These files will be removed from the grouped analysis shown in section C (as per

Figure 1).

C. Perform the grouped analysis

1. All assays will be analyzed in grouped manner, whether they yield consistent calls for all triplicates or not. Select the experiment that was set up for individual analysis, the ‘copy’ button on the top panel to make a copy of the selected experiment. Name it “Cmpd X grouped”. Click “Next”.

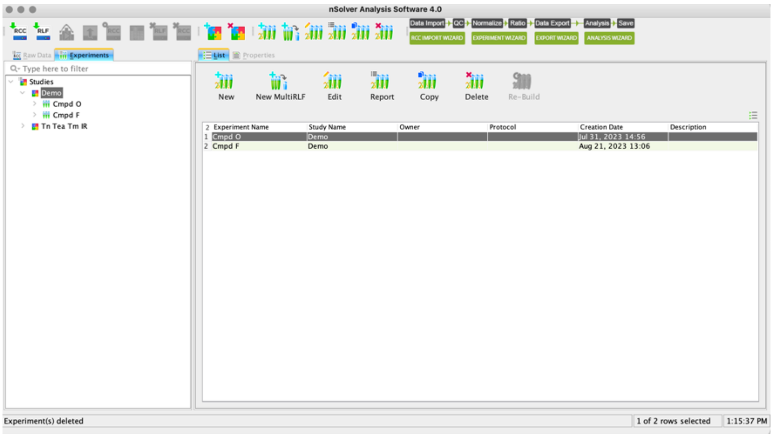

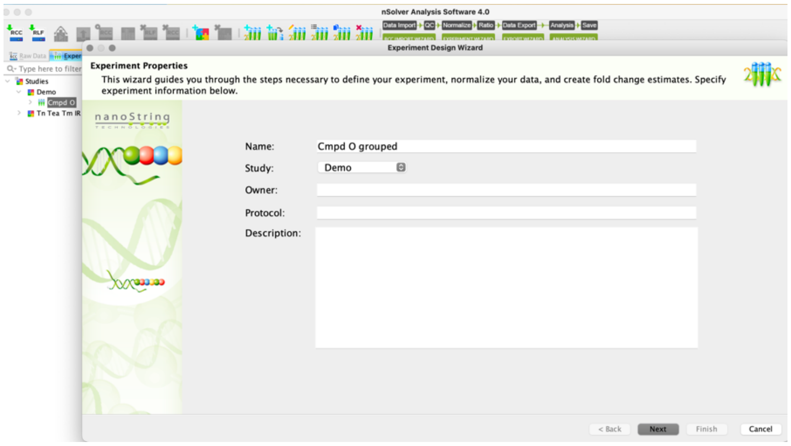

2. On the page “Add samples/Lanes” (you should see the 12 rcc files associated with this cmpd only at this step. If for any reason, other files show up, please select the 12 rcc files with this cmpd and click “Keep Selected” on the top panel):
    a. If all assays showed consistent calls no change needs to be made.
    b. If there are assay(s) that need to be removed, select the file(s) and click “Exclude Selected”.

   Click “Next”

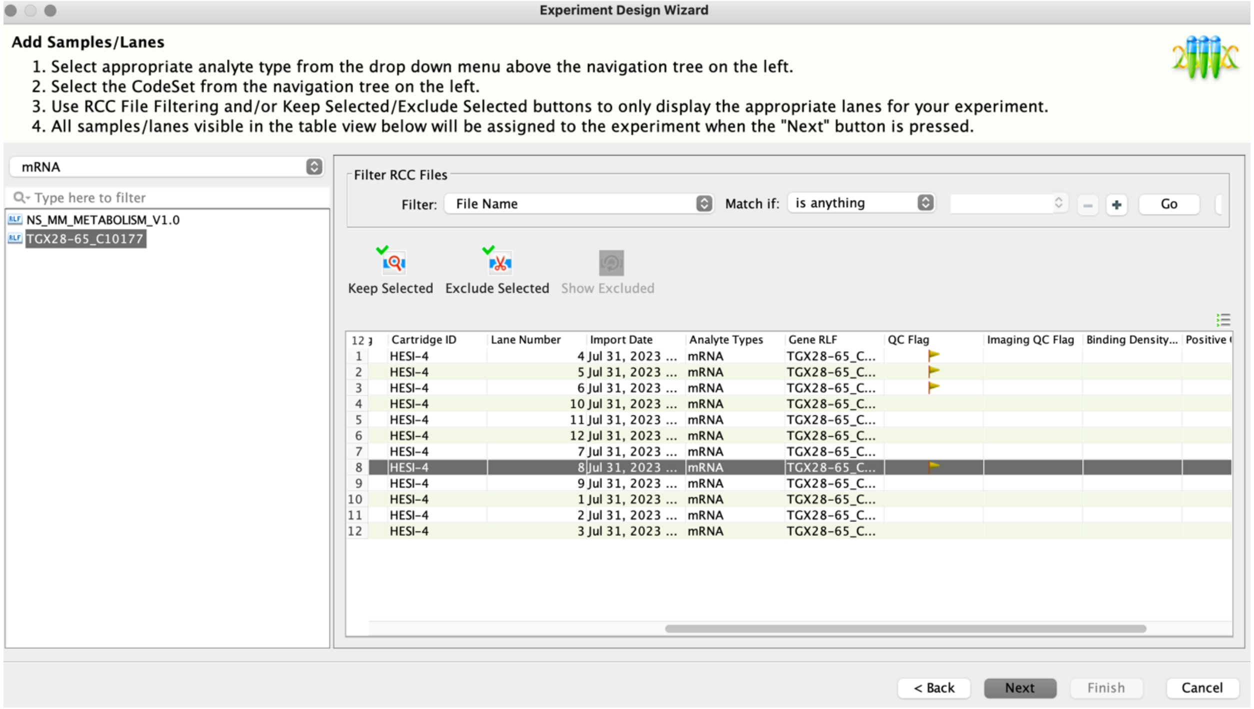

3. On the “Add Sample Annotation” page, click “Add Annotation” and fill in “Group” under the “Column Name”. Click ‘enter’ or ‘return’ (on Mac keyboard).

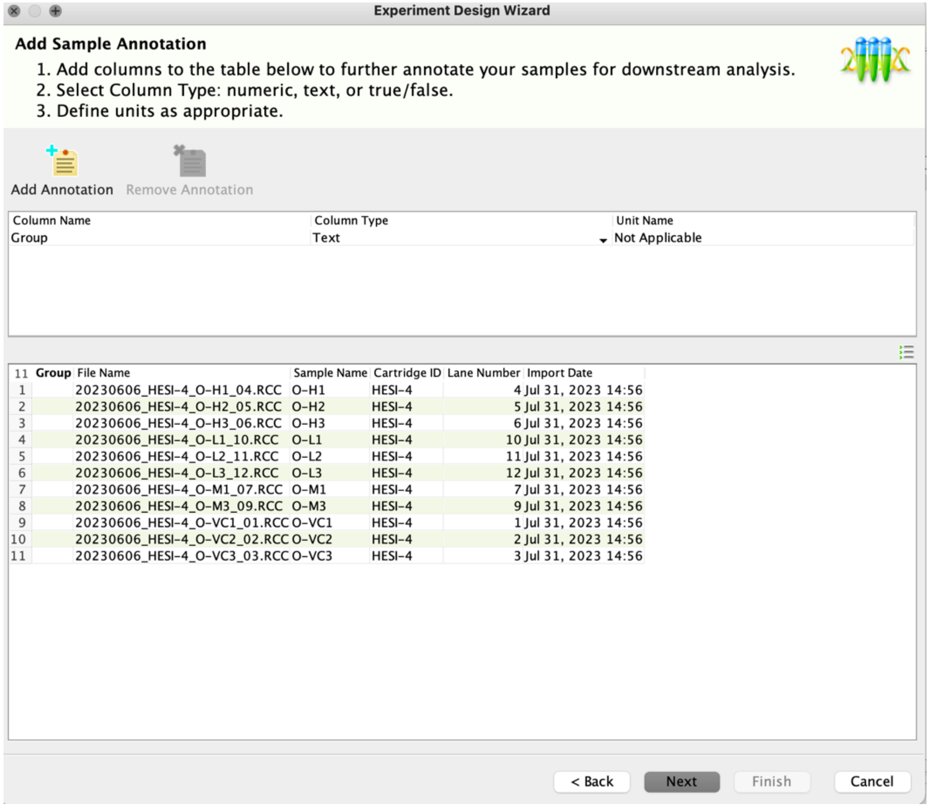

4. In the table, in the column of "Group", fill in the group name, [cmpd code]-[concentration, i.e. H/M/L/VC] as in the example below. Click "Next".

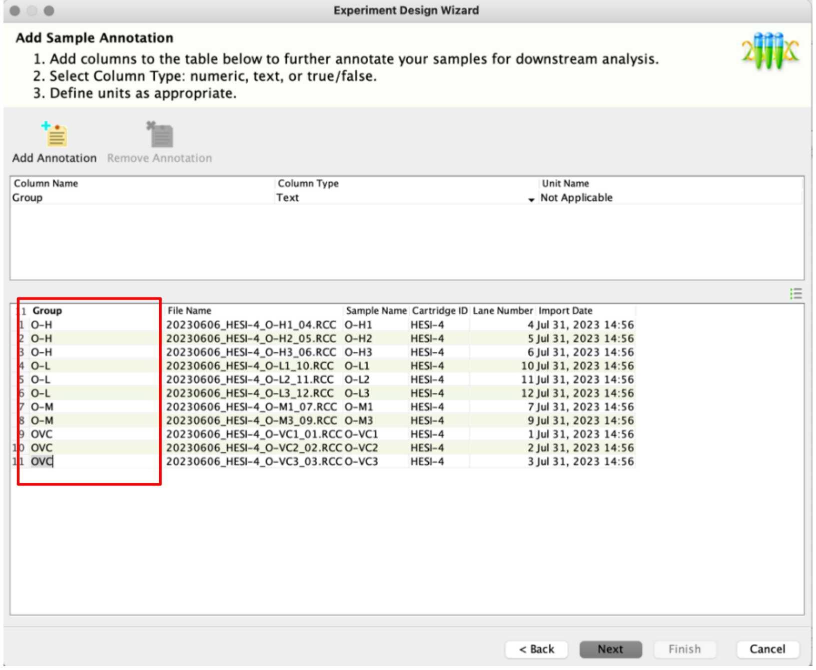

5. Select background subtraction and Normalization parameter as shown below.

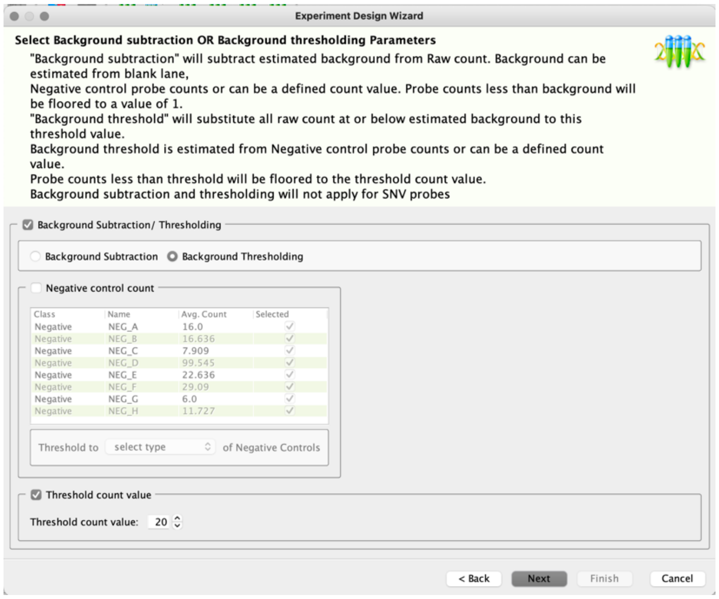

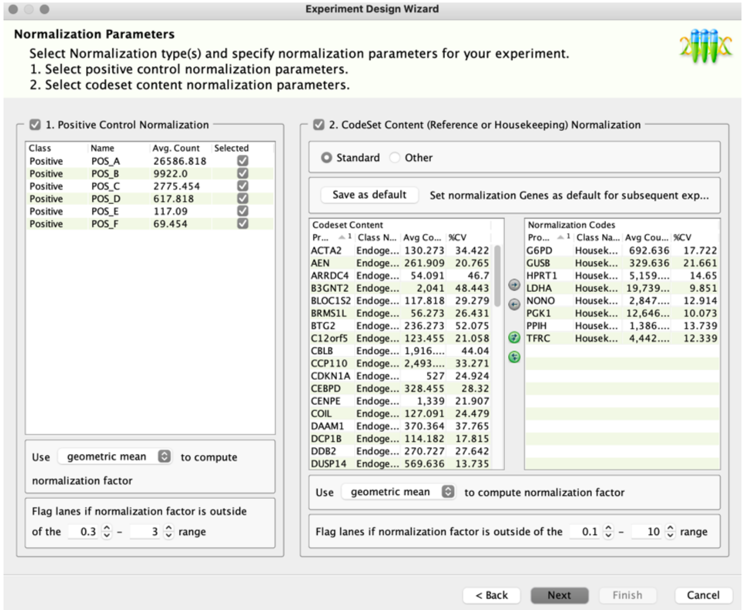

6. On page “Fold change Estimation”, select “Partitioning by” and choose “Group” using “xVC”. Click “Next”

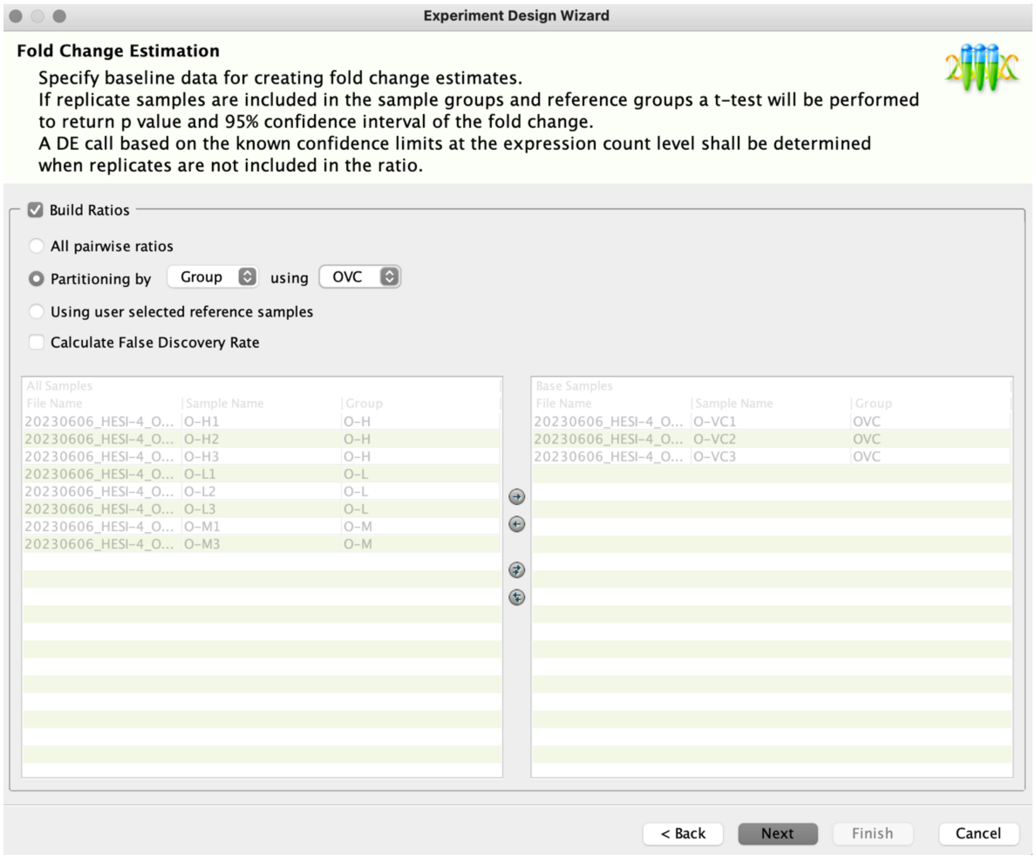

The Ratio Data Names page looks like the screenshot below. Click “Finish”.

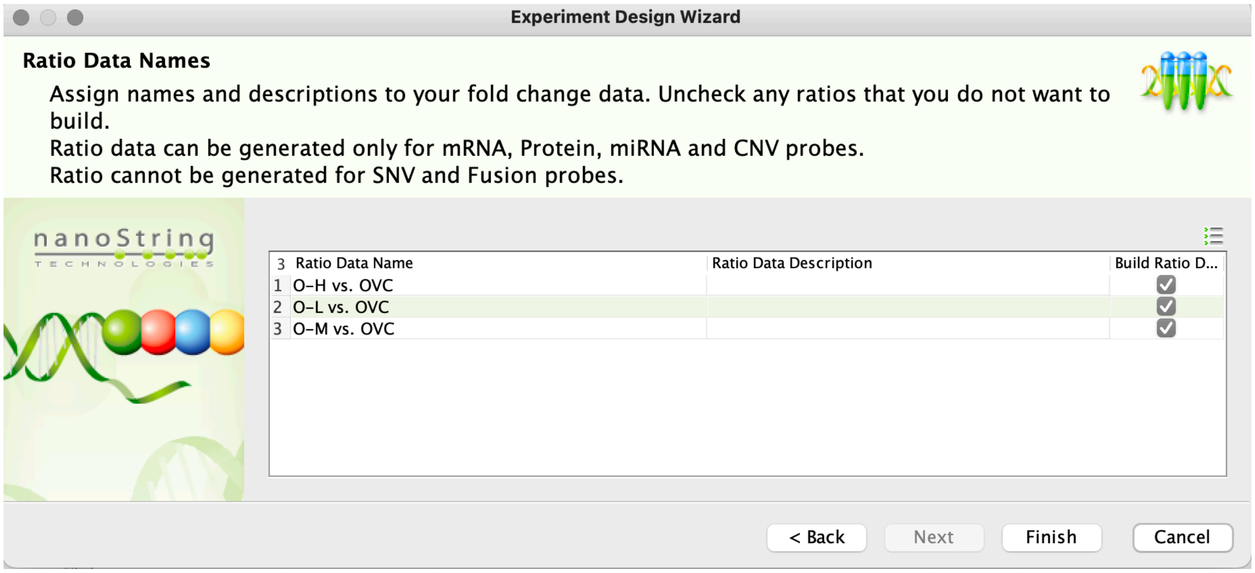

7. Select all listed data and click 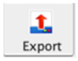 to export. Choose DEFAULT for Locale settings and TAB for Delimiter settings and then click "OK". This window may not appear in some versions of the software. In this case, proceed to the next step.

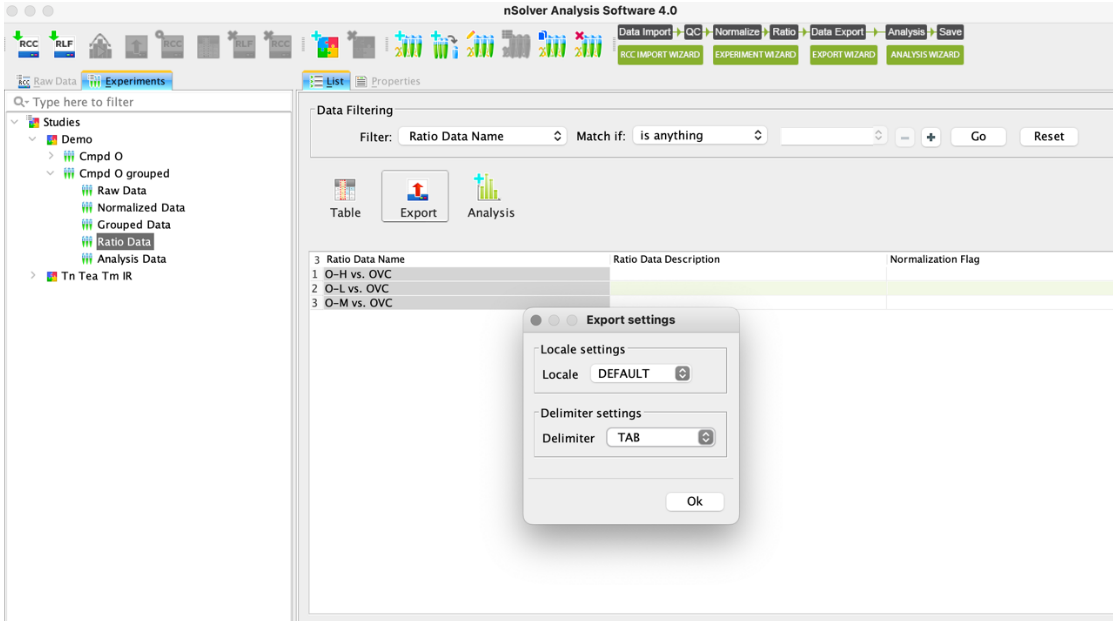

8. Keep the "Probe Name" and leave the selected data checked. Choose "Tab Delimited" for Format Options. Select Log2 Ratio for Ratio Options and then click "Finish".

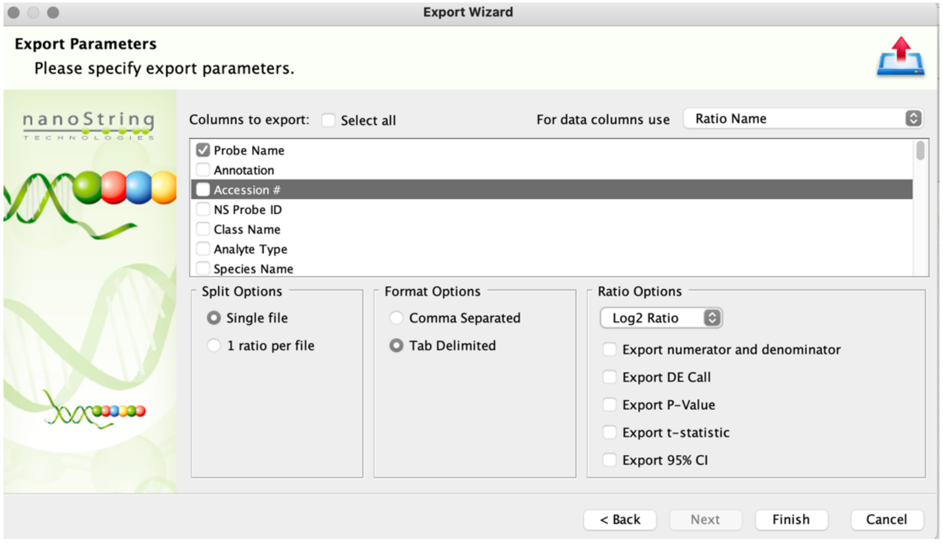

9. Name and save the Export Data as Text files (*.txt). Include "grouped" in the file name to distinguish from the output of the individual analysis.

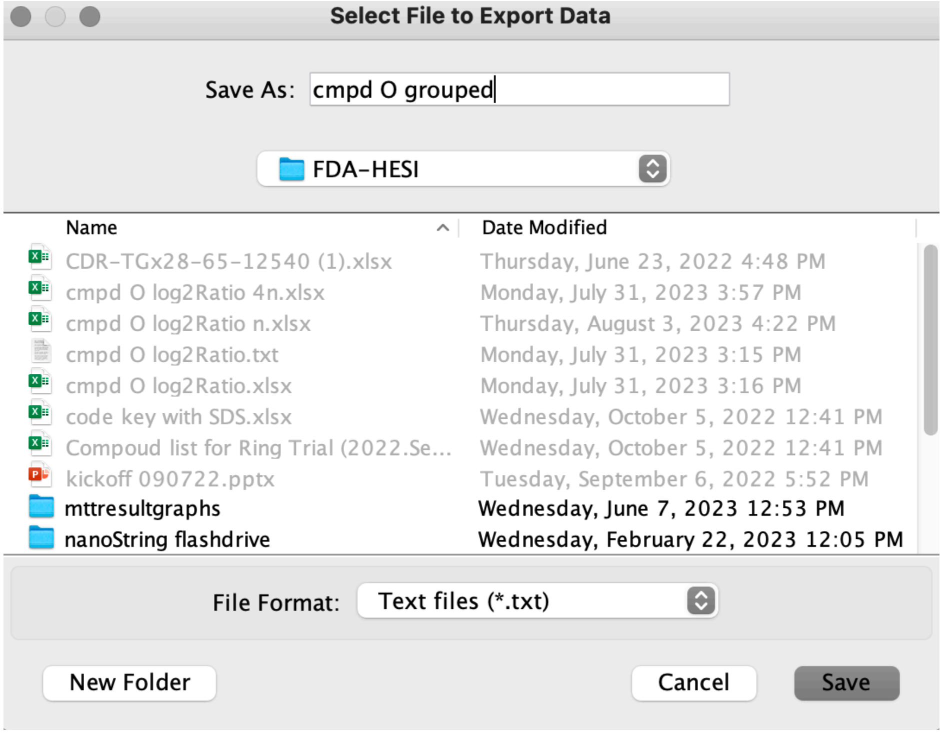

10. Format the data file for the TGx-DDI analysis using the NIEHS CEBS tool as described above, run, and get reports as described in Section A. 6 and 7.

D. Data Storage: Upload each analysis into your lab's folder in Sharepoint here.

- Each lab has two folders: one to store analysis by group average and the other to store analysis by individual replicate.

		Name		Modified	Modified By
		nSolver analysis by group average	•••	August 14	Chrissy Crute
		nSolver analysis by individual replicate	•••	August 14	Chrissy Crute

Drag files here to upload
