# Supplementary material for: TGx-DDI (toxicogenomic DNA damage-inducing) biomarker validation: multi-site ring trial supporting regulatory use
Source: Toxicol Sci. 2025 Oct 1;208(2):233–43. doi: 10.1093/toxsci/kfaf138 (PMC12646585; doi:10.1093/toxsci/kfaf138)
Supplement: kfaf138_Supplementary_Data [file kfaf138_supplementary_data.zip › kfaf138_Supplementary_Data/toxsci-25-0384-File009.pdf]

GENERIC TEST GUIDANCE

TGx-DDI Transcriptomic Biomarker

A composite *in vitro* transcriptomic biomarker to support a weight-of-evidence approach in assessing the genotoxicity of novel compounds

Table of Contents

GENERAL INTRODUCTION *3*

INITIAL CONSIDERATIONS AND LIMITATIONS *5*

PRINCIPLE OF THE TEST *7*

DEMONSTRATION OF PROFICIENCY *8*

PROCEDURE *10*

Overview **10**

Range Finding Experiments **11**

Cell Culture **12**

Biosafety 12
Cells 12
Maintenance of Cell Culture 13
Preparation of Cells for Chemical Treatment 13

Test Compound Preparation **13**

Step 1: Prepare the concentrated working solution and vehicle control solution 13
Step 2: S9 preparation 14

Chemical Exposure **14**

Treatment without S9 14
Treatment with S9 15
Cell Pellet Collection 15

Cytotoxicity Measurement **15**

RNA Extraction **16**

RNA Quality Control **17**

Gene Expression Analysis **17**

Data Analysis **18**

Quality Assurance/Quality Control (QA/QC) 18
NIEHS/CEBS TGx-DDI Biomarker Tool 19

Data Interpretation (Making A DDI Or Non-DDI Call) **20**

REFERENCES *22*

APPENDIX I. STATISTICS *24*

GENERAL INTRODUCTION

TGx-DDI (TGx = toxicogenomics; DDI = DNA damage-inducing) is a composite *in vitro* transcriptomic biomarker developed in TK6 human lymphoblastoid cells. It provides mechanistic insights into transcriptional changes in key DNA damage response pathways (e.g., p53 responsive genes), indicating whether the test compound causes DNA damage potentially relevant to *in vivo* human genotoxicity or carcinogenicity (1).

This TGx-DDI transcriptomic biomarker addresses the low specificity of standard *in vitro* chromosome damage assays in the current genotoxicity testing battery. It offers a novel method to improve genotoxicity assessments by classifying DNA damage-inducing agents based on transcriptomic data. TGx-DDI is proposed to be applied to agents tested positive in the in *vitro* assays indicating structural chromosome damage (clastogenicity) such as the *in vitro* mammalian cell chromosomal aberration assay or in vitro micronucleus assay, but not numerical changes (aneugenicity). Within a weight-of-evidence framework, it can be used to help interpret situations where the Ames test or *in vivo* micronucleus test outcomes are negative or inconclusive.

Extensive evidence already exists to demonstrate the accuracy and reproducibility of the TGx-DDI biomarker across multiple technical platforms, study sites, compound types, and cell lines (1–13). To date, 90 distinct compounds have been evaluated using this biomarker across six analytical platforms in more than four laboratories. The TGx-DDI biomarker is compatible with liver microsomal S9 extracts, enabling the evaluation of chemicals requiring metabolic activation in cell models lacking intrinsic metabolic competence (2,4,5,8,10). This compatibility extends its application to a broader range of test compounds and cell lines.

In summary, the application of TGx-DDI transcriptomic biomarker in a weight-of-evidence analysis presents a significant advance in the field of genotoxicity testing by addressing the low specificity of conventional *in vitro* chromosome damage assays. This biomarker has been rigorously validated, demonstrating high reproducibility, sensitivity, and specificity across multiple platforms and laboratory settings. The biomarker shows

exceptional accuracy in distinguishing DNA damage-inducing agents from irrelevant positive findings, reducing unnecessary additional follow-up *in vitro* and *in vivo* tests. The comprehensive wet and dry lab protocols are thoroughly refined and standardized, ensuring seamless transferability and reproducibility across laboratories. Furthermore, its robust performance in its intended context of use underscores the utility of TGx-DDI as a valuable tool for regulatory decision-making. By providing additional mechanistic insights into DNA damage responses, TGx-DDI enhances the weight-of-evidence approach in genotoxicity assessments, supporting safer and more efficient drug development processes.

This test guidance is formulated based on an international cross-laboratory validation study conducted from 2023 to 2024, currently under review by the US Food and Drug Administration (FDA) for qualification. In this FDA-funded ring-trial study, the TGx-DDI biomarker was validated in four study sites using 14 test compounds, comprising six DDI and eight non-DDI agents covering a broad range of mechanisms, in TK6 human lymphoblastoid cells. Positive controls (bleomycin and benzo[a]pyrene) and a negative control (caffeine) were incorporated to ensure test performance. Herein, this guidance outlines procedures for applying the TGx-DDI biomarker to produce classification calls in diverse laboratory settings for novel compound testing.

INITIAL CONSIDERATIONS AND LIMITATIONS

The TGx-DDI biomarker is first developed in human TK6 lymphoblastoid cells and extensively validated using TK6 cells and HepaRG hepatocytes, two widely accepted *in vitro* models for genotoxicity testing. These two cell lines remain the preferred cell models for TGx-DDI analysis. However, the molecular basis of this biomarker indicates its applicability to any cell type with a functional p53 signaling pathway. TGx-DDI is less effective in cells with weak or impaired p53 responses and thus verifying the p53 gene status of the chosen cell line is crucial (14).

The metabolic activation of test compounds can significantly influence their biological effects and the resulting transcriptional responses. In TK6 cells, the co-treatment with the liver microsomal S9 extracts enables TGx-DDI to effectively classify compounds that require metabolic activation (2,4,5,8,10). TGx-DDI demonstrates 100% sensitivity, 86% specificity, and 91% overall accuracy in classifying DDI and non-DDI agents in TK6 cells (FDA-funded ring-trial study). Similarly, TGx-DDI achieves a predictive accuracy of 90% in metabolically competent HepaRG hepatocytes but reduced accuracies of 60% and 80% in HepG2 cells and embryonic stem cell-derived hepatocytes (15). If a selected cell line lacks metabolic competence, it is essential to verify the expression of key enzymes responsible for the metabolism of the compounds of interest. Alternatively, incorporating a metabolic activation system, such as S9, is essential to ensure data reliability.

The TGx-DDI classification relies on the expression profiles of a panel of 64 biomarker genes (1,2). Ensuring that a robust signal is achieved in the profiling process is important to minimize errors and enhance data reliability. For the former, Quality Assurance/Quality Control (QA/QC) parameters in standard bioinformatic pipelines can be used to confirm sufficient signal from the technology. Additionally, evaluating transcriptional responses in key stress response genes (e.g., *ATF3*, *CDKN1A*, *GADD45A*, and *BTG2*) (1)(7) can help identify potential issues, such as a lack of cellular response or dosing errors, as reported in our previous studies (2).

The TGx-DDI biomarker, enriched for transcripts regulated by p53, may produce false-positive results with compounds that target the p53 pathway (e.g., p53 activators) or closely related signaling pathways (e.g., kinase inhibitor-based cancer therapeutics). These compounds can elicit positive responses in TGx-DDI without causing DNA damage. Similarly, aneugens that disrupt chromosomal segregation without affecting p53 are typically classified as non-DDIs, as expected. We also note that TGx-DDI is not amenable to testing compounds that inhibit transcriptional machinery. For instance, nucleoside reverse transcriptase inhibitors (e.g., Zidovudine) are likely to be classified as non-DDIs due to their mechanism, which prevents the upregulation of stress response genes (12). Moreover, TGx-DDI exhibits lower sensitivity for detecting antimetabolites, which may require longer exposures to show effects (2). Users are advised to consider the mechanism of action of the compound under test, and where justified, alternative exposure durations may be used to address potential delayed effects.

As a complementary tool, TGx-DDI is easily incorporated with various traditional and newer genotoxicity tests and toxicological endpoints. Improved predictive accuracies are achieved when TGx-DDI is used in conjugation with other standard genotoxicity biomarkers and tests, such as *in vitro* micronucleus assay (4–7,10,11), CometChip (9,12), and MultiFlow (10). The combination of TGx-DDI with other tests addresses their respective limitations and demonstrates improved test performance.

Based on these considerations, we recommend integrating TGx-DDI with *in silico* or other *in vitro* tests to evaluate the TGx-DDI calls within a weight-of-evidence framework; other factors such as the pharmacokinetics and the mechanisms of the test compounds (when available) should be considered together with the test results.

Lastly, the TGx-DDI biomarker is proposed to serve as an additional line of evidence rather than a replacement for standard genotoxicity assessments. Importantly, TGx-DDI is developed specifically for nonclinical *in vitro* use and is not intended for direct application in humans.

PRINCIPLE OF THE TEST

The TGx-DDI biomarker classification is based on transcriptomic profiling to identify DDI compounds by detecting specific changes in the expression of 64 genes, primarily regulated by the p53 signaling pathway. A conservative three-pronged analytical approach is applied to make an overall TGx-DDI classification: nearest shrunken centroid probability analysis (PA), principal component analysis (PCA), and hierarchical cluster analysis (HCA) with a reference panel of prototype agents. A test chemical is classified as DDI, non-DDI, or inconclusive by comparing its gene expression profile in exposed cells to reference profiles derived from known genotoxic and non-genotoxic compounds.

A 3- to 4-hour exposure window is selected to capture early transcriptional responses associated with DNA damage. Longer exposure may introduce secondary chromosomal damage caused by cytotoxicity or general cellular stress, which could lead to misclassification or false positives.

Selection of top concentrations for TGx-DDI analysis is critical to ensure the validity of test outcomes, as it is for all *in vitro* genotoxicity tests. Cytotoxicity thresholds (defined in section “Range Finding Experiments”) are established to prevent false-negative DDI calls resulting from concentrations that are not sufficiently high. These thresholds are consistent with those used in standard genotoxicity assays (e.g., OECD Test Guidelines 473 and 487) (16,17). Concentrations that are overtly cytotoxicity (> 60%) should be excluded, as they can compromise the transcriptional machinery and cell integrity, affect gene expression levels, and reduce test reliability.

DEMONSTRATION OF PROFICIENCY

Laboratories are required to demonstrate technical proficiency using the technology that they intend to use for the analysis (or the first time they apply the biomarker in regulatory testing).

Test exemption: A proficiency test may be waived if the laboratory outsources the analysis fully to qualified research companies:

- Inotiv (https://www.inotiv.com/services/in-vitro-toxicology)
- ScitoVation (https://scitovation.com/services/cell-based-assays/genotox/).

The laboratory should perform two sets of experiments:

1) Apply the TGx-DDI protocol to classify the positive/negative control compounds (caffeine, bleomycin, benzo[a]pyrene), if the expected TGx-DDI calls are obtained;

2) Test an additional set of 5 compounds including two DDI agents not requiring metabolic activation, one DDI agent that requires metabolic activation, and two non-DDI agents. Table 1 provides a list of recommended compounds for demonstrating technical proficiency with the TGx-DDI analyses. These compounds exhibit robust and reproducible results with TGx-DDI across cell lines and platforms.

The laboratory is considered proficient if correct DDI calls are produced for all the compounds. Testing should be carried out in full adherence with the SOPs provided for the cell culture treatments, analytical workflow, and TGx-DDI results interpretation. We have established a collaboration with the non-profit organization US Pharmacopeia (USP) to develop a TGx-DDI proficiency testing library. USP specializes in small molecule reference libraries for drug development (https://www.usp.org/). The full TGx-DDI proficiency testing library will be made available in their catalogue to provide

quality assured and validated compounds for labs to establish this method, or to bridge into new platforms.

Table 1. Recommended compounds for TGx-DDI proficiency testing.

Compounds	CAS No.	M.W.	Metabolic Activation	Expected DDI call
**Set 1**				
Caffeine	58-08-2	194.19	N	–
Bleomycin (sulfate)	9041-93-4	1415.60	N	+
Benzo[a]pyrene	50-32-8	252.31	Y	+
**Set 2**				
Etoposide	33419-42-0	588.56	N	+
EMS (Ethyl methanesulfonate)	62-50-0	124.16	N	+
D-Mannitol	69-65-8	182.17	N	–
Ampicillin	69-53-4	349.40	N	–
Cyclophosphamide	6055-19-2	279.1	Y	+

Y, yes; N, no.

CAS No. = Chemical Abstracts Service Registry Number

*Test concentrations need to be adjusted based on the responses of the selected cell lines, cell batches, and other experimental factors.

PROCEDURE

Overview

Figure 1 presents a standard experimental workflow for testing novel compounds using the TGx-DDI biomarker. Further instructions for each step are provided in the following sections.

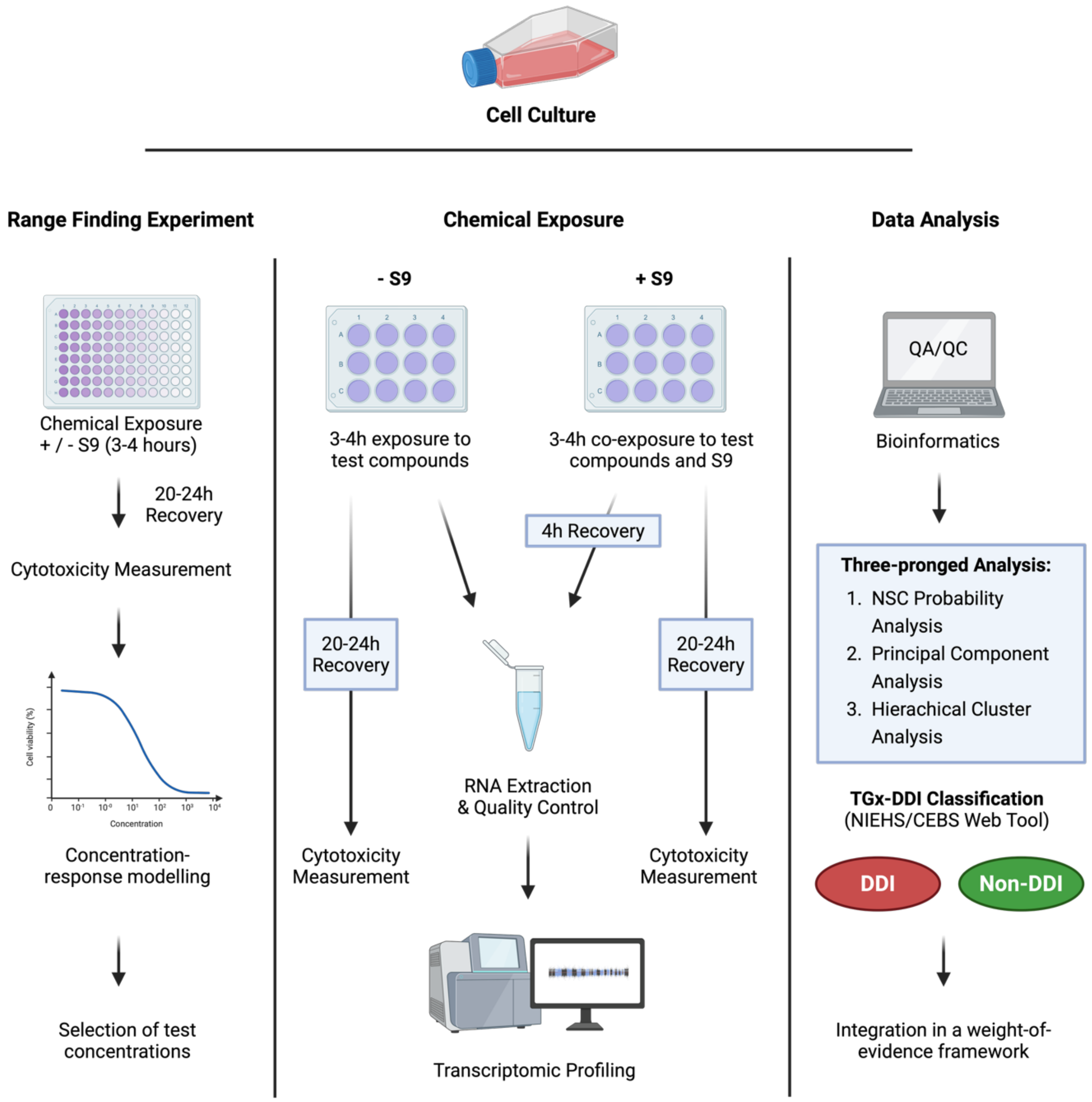

Figure 1. Experimental workflow of the TGx-DDI assay (created in BioRender).

Range Finding Experiments

The selection of top concentrations is critical to ensure the validity of test outcomes. The laboratories may determine the test concentrations based on previously available cytotoxicity data for the test compounds. When no additional information is available, laboratories are required to start with range-finding experiments to ensure that the test concentrations meet the required cytotoxicity.

Typical procedures

Begin testing each compound at a starting concentration of 1 mM or 500 µg/mL, whichever is lower, or at the concentration where precipitation is observed. This approach aligns with the guidelines and is appropriate for cases where no prior information about the compound is available. The cells are exposed to the solvent or up to eight concentrations (with a spacing factor of 2–3) of the compound in the presence or absence of S9 for 3-4 hr, followed by a 20-24 hr recovery period (see procedures in section "Chemical Exposure"). The test can be done in single replicates with at least n=2. Cytotoxicity should be assessed using any assays capable of reliably measuring cell viability, cell death, or proliferation. Examples include the MTT cell proliferation assay as used in previous TGx-DDI validation studies, or Relative Population Doubling (RPD) or Relative Increase in Cell Count (RICC) as recommended in OECD Test Guidelines 473 and 487 (16,17).

We recommend selecting a minimum of three concentrations (and five as optimal) for the test, including a "high" concentration maintaining 40–50% viability, a "middle" with 50–80% viability, and a "low" with >80% viability. The selected top concentrations should meet the following cytotoxicity thresholds: (1) induce 50%-60% cytotoxicity; or (2) at the solubility limit*; or (3) exceed 1 mM.

*Solubility limit refers to the maximum concentration of a substance that can dissolve in a solvent and no precipitate is visible by eye or with the aid of an inverted microscope in the culture medium at the end of the treatment.

Cell Culture

Biosafety

Cell culture procedures should be conducted following standard aseptic techniques to maintain sterility and prevent contamination. All cell handling must be performed in a Class II Biosafety Cabinet. Cells, media, and reagents should be treated as potentially infectious. Proper biosafety precautions, including the use of appropriate personal protective equipment such as gloves, lab coats, and eye protection, should be followed at all times. All spent media and disposable materials must be autoclaved before disposal to ensure biological safety. Routine cleaning and decontamination of work areas, incubators, and equipment should be performed using appropriate disinfectants.

Cells

TGx-DDI has been extensively validated in TK6 human lymphoblastoid cells and HepaRG hepatocytes. We recommend using these two cell lines for testing new compounds. Nevertheless, TGx-DDI may be adaptable to other human or mammalian cells with functional p53 signaling pathways. Laboratories must complete proficiency tests (refer to section "demonstration of proficiency") using their selected cell lines. Cell cultures should be regularly monitored for contamination and maintained under optimal growth conditions to ensure experimental reproducibility and reliability.

We recommend obtaining qualified TK6 cells from a reputable source (e.g., ATCC or other recognized cell repositories) to ensure cell line authenticity and consistency. The number of subcultivations should be limited to minimize the potential for genetic drift or phenotypic variation over time. For long-term culture maintenance, periodic monitoring of TK6 cell stability is advisable to confirm the continued suitability of the cell line.

Notably, when using cell models with limited endogenous metabolic capacity, an exogenous metabolizing system should be incorporated to ensure the bioactivation of test compounds (as utilized in TK6 cells) (2,4,5,8,10).

Maintenance of Cell Culture

Cells should be maintained in an appropriate culture vessel (e.g., T-flask) with suitable growth medium and incubated under optimal conditions (e.g., 37°C, 5% CO_2). Regular monitoring under an inverted microscope is recommended to assess cell health, morphology, and confluency. Cell density should be maintained within the recommended range for the specific cell line in use. Before experimental use, cells should be cultured for at least two passages to ensure recovery and normal growth characteristics.

Preparation of Cells for Chemical Treatment

Prior to treatment, cells should be counted and diluted to the appropriate seeding density to ensure a sufficient number of cells for RNA extraction and subsequent sequencing analysis. Cell should be seeded in suitable culture vessels (e.g., 12-well plates) and incubated overnight to allow for proper attachment and acclimation before chemical exposure.

Test Compound Preparation

Step 1: Prepare the concentrated working solution and vehicle control solution

Ideally, this step should be performed immediately before cell treatment. Saline or cell culture medium are the preferred solvents/vehicles for dissolving the test chemical. If the test chemical does not dissolve or remain stable in saline or culture medium, DMSO can be considered as the secondary option. Other solvents/vehicles may be used if supported by sufficient scientific rationale. The stability of the test chemical in the final solvent/vehicle must be taken into consideration.

Note: The term ‘test compound’ in this Test Guidance refers to the substance being tested, regardless of whether it is a mono-constituent substance, multi-constituent substance, or mixture. While limited information is available on the applicability of TGx-DDI to multi-constituent substances and mixtures, these test methods are technically applicable for testing such substances.

We recommend preparing the test compounds in 100X concentrated working solution of the top concentration and then diluted in culture medium to the designated test concentrations. A corresponding vehicle control solution is prepared in culture medium to match the final conditions of the treated samples.

Step 2: S9 preparation

If the experiment includes a metabolic activation system, the recommended system is typically a co-factor-supplemented post-mitochondrial fraction (S9) derived from the livers of rodents, often rats, treated with enzyme-inducing agents such as Aroclor 1254 or a combination of phenobarbital and β-naphthoflavone (16,17). The recommended S9 fraction concentration ranges from 1 to 2% (v/v) in the final test medium, with a treatment duration of 3–4 hours to avoid excessive cytotoxicity caused by prolonged exposure. For preparation, follow the specific instructions provided for any pre-made S9 mixture or system used. A fresh working solution should be prepared immediately before the cell treatment.

Chemical Exposure

Positive and negative controls should be included in each batch of experiments to ensure assay performance and data interpretability. While the use of duplicate cultures at each concentration is advisable, either replicate or single treated cultures may be used, consistent with OECD Test Guidelines for in vitro mammalian assays. In addition, researchers are encouraged to explore the integration of independent repeat experiments, where feasible, as these can capture broader sources of variability and further strengthen assay robustness.

Treatment without S9

In tests without S9, cells are treated for 3 to 4 hours at selected concentrations with chemicals and corresponding controls.

- **For cytotoxicity measurement**: transfer a portion of cells from each well of the culture vessel to three wells of a smaller plate (96-well plate, 100 µl per well).

Centrifuge the 96-well plates at 1000 rpm for 5 min, discard the supernatant, and add 100 µl of fresh medium. Incubate the plates in a 37°C incubator with 5% CO_2 for an additional 20-24 hours. After the recovery period, perform the cytotoxicity measurement following the manufacturer's instructions.

- **For RNA extraction**: the remaining cells are harvested immediately after the 3 to 4-hour treatment.

Treatment with S9

Benzo[a]pyrene should be included in the test as a positive control. The S9 fraction (or equivalent) is added to the cells along with the compound solutions and corresponding solvent controls at selected concentrations. The cells are exposed for 3 to 4 hours.

- **For cytotoxicity measurement**: as with treatments without S9, transfer the cells to smaller wells, centrifuge, replace the supernatant with fresh medium, and incubate under standard conditions. After the recovery period (e.g., 20-24 hours), perform the cytotoxicity measurement according to the manufacturer's instructions.
- **For RNA extraction**: after the 3-4 hour treatment, the treatment medium is replaced with fresh medium for the remaining cells and incubated for a short additional period (e.g., 4 hours). Cells are then harvested for RNA extraction.

Cell Pellet Collection

This procedure applies for both treatments with and without S9. To harvest cells for RNA extraction, collect the cell suspension with medium in 15 ml conical tubes, centrifuge at 1000 rpm for 5 min. Carefully remove medium and gently resuspend cells in 1 ml of 1X Phosphate Buffered Saline without calcium and magnesium. Transfer the cell suspension to a new tube, then centrifuge again at 4,000 rpm for 5 min. Remove PBS and store the cell pellets at –80°C until RNA extraction.

Cytotoxicity Measurement

Cytotoxicity of the test compounds should be assessed in parallel using any appropriate assays capable of reliably measuring cell viability, cell death or proliferation.

To proceed with further analysis, the cytotoxicity should meet the following criteria: The top concentration should induce 55% ± 5% cytotoxicity, unless the concentration is > 1mM or the compound reaches its solubility limit. Additionally, concentrations that are overtly cytotoxicity (i.e., cytotoxicity > 60%) should be excluded for further experiments. If a majority of samples in any test condition fail to meet these criteria, it is recommended to redefine the test concentrations by running an additional range-finding experiment.

RNA Extraction

Note: This step may be omitted if the selected gene expression analysis method is compatible with direct use of cell lysates.

Laboratories may choose from commercially available RNA extraction kits or utilize custom protocols tailored to their experimental setup. In either case, the method should ensure the isolation of intact RNA with minimal contamination by proteins, DNA, or other substances that could interfere with the subsequent gene expression analysis. Any deviations from standard protocols or kit instructions should be documented and justified.

Cell pellets can be stored at -80°C for later use or used directly in the procedure. Typically the samples are stored for less than one month and subjected to only one freeze and thaw cycle.

Maintaining sterility is critical throughout the assay to prevent contamination. Proper storage of samples and RNA at -80°C is essential to avoid RNA degradation. Adherence to RNA sterile handling techniques is mandatory for TGx-DDI analysis. This includes ensuring that work areas, equipment, and consumables are RNase-free and using certified RNase-free reagents and materials.

RNA Quality Control

Note: This section is not required for laboratories sending out their samples to sequencing service.

Prior to any TGx-DDI analysis, RNA quality should be assessed. Only samples with RNA Integrity Number (RIN) > 7 should be used. An Agilent Bioanalyzer or TapeStation or other instruments can be used following instructions and kits provided by the manufacturer. Additional RNA quality metrics, such as the ratio of the absorbance readings at 260 nm and 280 nm (A260/A280), should be considered if they may affect the subsequent gene expression analyses. A ratio between 1.8 to 2.0 typically indicates pure RNA.

If the sample is stored for more than one month at -80°C and subjected to more than one freeze and thaw, the RNA quality must be reassessed to confirm that it meets the required criteria before the sample is used in further experiments.

Gene Expression Analysis

Note: This section is not required for laboratories sending out their samples to sequencing service.

The TGx-DDI has demonstrated high sensitivity and specificity in various transcriptomic platforms, including microarray, NanoString, RT-qPCR, RNA-Seq, and TempO-Seq. Laboratories have the flexibility to select a platform that aligns with their operational capabilities, provided they adhere to the kit- and platform-specific manufacturer instructions. To ensure reliability and validity of the results, laboratories must complete proficiency tests (refer to section “demonstration of proficiency”) using their selected platform before proceeding to analyze new test compounds.

We recommend that laboratories include the following set of reference RNA during routine testing:

- Human Universal Reference RNA - uhrRNA Agilent Cat# 740000, Santa Clara, California
- Human Brain Total RNA brRNA - ThermoFisher AM7962, Waltham, Massachusetts

These reference standards can be used to identify sources of technical error (e.g., library preparation failures) and track performance of the gene expression technology across experiments and time.

Data Analysis

Raw sequencing data should be analyzed following standard workflows. Any software versions, parameters, and deviations from standard protocols must be documented. Laboratories should ensure proper data storage, sharing, and reporting, and submit data to repositories as required.

Quality Assurance/Quality Control (QA/QC)

The analysis pipeline should begin with platform specific QA/QC of raw sequencing reads to assess read quality, using tools such as the NanoString nSolver™ Analysis Software, FastQC, or equivalents. Samples that fail to meet the QC criteria or generate QC flags should be excluded from further analysis. Prior to proceeding, ensure there are sufficient solvent controls and experimental samples (at least n=2) to proceed with the analysis for each compound. A workflow is summarized in Figure 2.

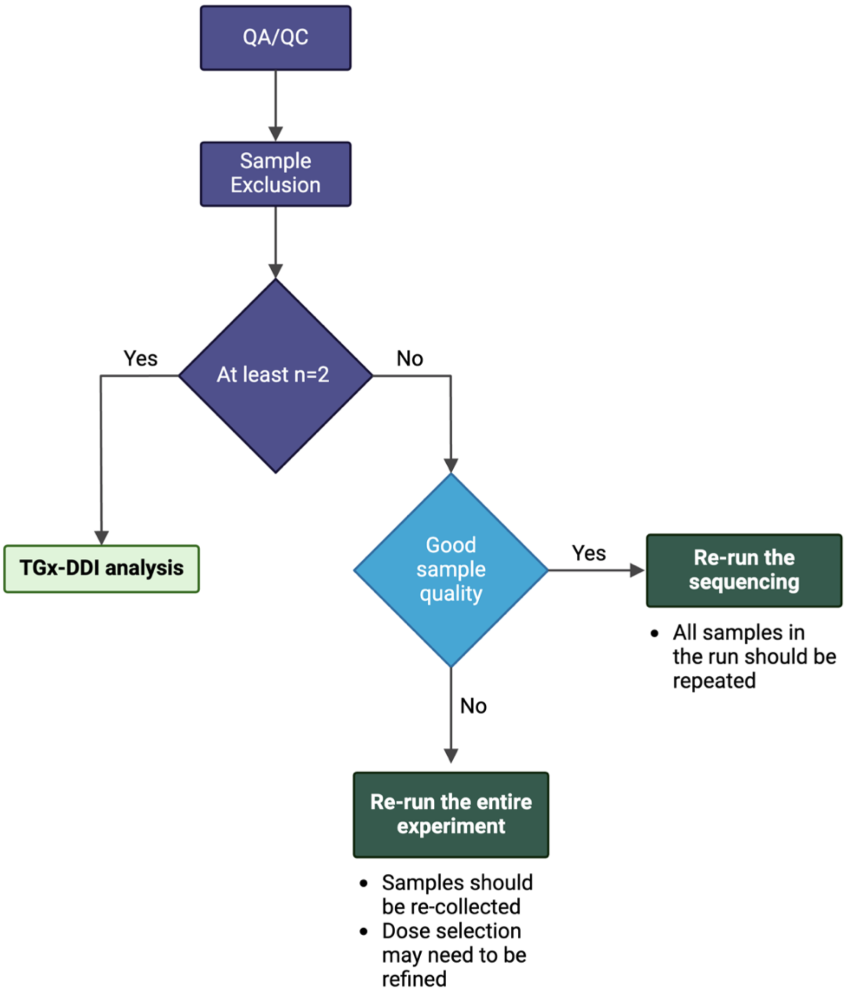

Figure 2. Flow-chart of post-QC assessment of sample replicates.

If a sample has to be removed, the analysis can continue with the rest of the samples if at least two replicates from that treatment group are retained. If a majority of the samples are excluded from the replicates (e.g., two out of three), the sequencing assay should be re-run using samples that pass the quality check and cytotoxicity thresholds. All samples in the experiment should be repeated. If the remaining RNA is not enough for the repeats, or the RNA quality does not meet the specified criteria (e.g., RIN > 7), the entire experiment should be repeated starting from the cell culture step. In a case that the samples fail to meet the cytotoxicity thresholds, it is recommended to refine the dose selection by repeating range-finding experiments (created in BioRender).

NIEHS/CEBS TGx-DDI Biomarker Tool

Data are normalized by using the geometric mean of positive control counts in the codeset and exported as gene counts. Fold change is calculated and $\log_2$ transformed

for each chemical at each concentration and saved as a tab-delimited text file. Gene Symbols should be in the first column, followed by the log_2 fold changes in subsequent columns that are labeled according to data type (e.g., chemical) and/or concentration. The data are submitted to the online TGx-DDI Biomarker for DNA Damage Classification Tool (https://cebs.niehs.nih.gov/tgxddi/tool) for making a classification call. The software tool only requires the user to enter the test chemical name and the file name to be uploaded to the tool. By clicking “Submit” the online tool conducts all the necessary analysis for the TGx-DDI biomarker and produce classification calls. Using the data interpretation procedure outlines in Section “Data Interpretation”, the laboratories should apply the three-pronged approach for each concentration. A chemical should be classified as DDI positive if any of the three concentrations are classified as DDI. A chemical should be classified as DDI negative if for all concentrations the three-pronged approach produce a non-DDI classification. Detailed SOPs for using the NIEHS/CEBS web tool for TGx-DDI data analysis can be found in annexes of the TGx-DDI Biomarker Final Qualification Report.

Data Interpretation (Making A DDI Or Non-DDI Call)

The calculation of DDI probability by the online tool is solely based on the nearest shrunken centroid probability classifier (PA). To apply the three-pronged approach, users should examine the principal component analysis (PCA) and hierarchical cluster analyses (HCA) by clicking on the clustering link in the online tool. A detailed explanation of the statistical analyses performed for the three-pronged approach is provided in Appendix I Statistics.

The overall DDI classification was derived through a step-by-step process.

1. At the sample level, each experimental sample received a classification call. If a positive DDI call was made in any of the analysis methods (PA, PCA, HCA) in the three-pronged approach, the sample was classified as DDI. If all the methods generated a non-DDI call, the compound was classified as non-DDI. Compounds may produce an 'inconclusive' call under specific conditions: when the PA probability is below 0.9 for either DDI or non-DDI classification, when the

compound falls on the PC1 axis, or if the compound does not fall distinctly on either the DDI or non-DDI branch of the dendrogram. For samples without a definitive DDI classification, a majority rule was used to classify them as non-DDI or inconclusive.

2. A grouped analysis was then conducted to classify each concentration of a compound. As with the classification of individual samples, if a majority of samples (e.g., two out of three) within a concentration produced a DDI call, the concentration was called DDI in the 'grouped analysis'. Similarly, samples were classified by the majority rule as either non-DDI or inconclusive.
3. Finally, a compound was classified as DDI if a DDI call was made at any concentration (else it was considered non-DDI or inconclusive).

Note that overtly cytotoxic concentrations (i.e., viability <40% based on cytotoxicity measurement at 24 hr) should not be used for classification. Chemicals that produce a DDI call at any concentration meeting the cytotoxicity thresholds will be classified as DDI.

Overall, any DDI call in the three-pronged approach will lead to a DDI call. If an ‘inconclusive’ call is made in any of the three classifications, the overall call for the chemical would be based on the majority (e.g., one ‘inconclusive’ in PCA, but non-DDI for HCA and PA = non-DDI). If all three calls are ‘inconclusive’, the chemical cannot be classified using the TGx-DDI biomarker.

REFERENCES

1. Li H, Hyduke DR, Chen R, Heard P, Yauk CL, Aubrecht J, et al. Development of a toxicogenomics signature for genotoxicity using a dose-optimization and informatics strategy in human cells. Environ Mol Mutagen. 2015;56(6):505–19.

2. Li HH, Chen R, Hyduke DR, Williams A, Frötschl R, Ellinger-Ziegelbauer H, et al. Development and validation of a high-throughput transcriptomic biomarker to address 21st century genetic toxicology needs. Proc Natl Acad Sci. 2017;114(51):E10881–9.

3. Cho E, Buick JK, Williams A, Chen R, Li H, Corton JC, et al. Assessment of the performance of the TGx-DDI biomarker to detect DNA damage-inducing agents using quantitative RT-PCR in TK6 cells. Environ Mol Mutagen. 2019;60(2):122–33.

4. Buick JK, Moffat I, Williams A, Swartz CD, Recio L, Hyduke DR, et al. Integration of metabolic activation with a predictive toxicogenomics signature to classify genotoxic versus nongenotoxic chemicals in human TK6 cells. Environ Mol Mutagen. 2015;56(6):520–34.

5. Yauk CL, Buick JK, Williams A, Swartz CD, Recio L, Li H, et al. Application of the TGx-28.65 transcriptomic biomarker to classify genotoxic and non-genotoxic chemicals in human TK6 cells in the presence of rat liver S9. Environ Mol Mutagen. 2016;57(4):243–60.

6. Buick JK, Williams A, Kuo B, Wills JW, Swartz CD, Recio L, et al. Integration of the TGx-28.65 genomic biomarker with the flow cytometry micronucleus test to assess the genotoxicity of disperse orange and 1,2,4-benzenetriol in human TK6 cells. Mutat ResFundam Mol Mech Mutagen. 2017;806:51–62.

7. Allemang A, Abrew KND, Shan YK, Krailler JM, Pfuhler S. A comparison of classical and 21st century genotoxicity tools: A proof of concept study of 18 chemicals comparing in vitro micronucleus, ToxTracker and genomics-based methods (TGx-DDI, whole genome clustering and connectivity mapping). Environ Mol Mutagen. 2021;62(2):92–107.

8. Chen R, Lin YT, Fornace AJ, Li HH. A high-throughput and highly automated genotoxicity screening assay. ALTEX. 2021;39(1):71–81.

9. Buick JK, Rowan-Carroll A, Gagné R, Williams A, Chen R, Li HH, et al. Integrated Genotoxicity Testing of three anti-infective drugs using the TGx-DDI transcriptomic biomarker and high-throughput CometChip® assay in TK6 cells. Front Toxicol. 2022;4:991590.
10. Fortin AMV, Long AS, Williams A, Meier MJ, Cox J, Pinsonnault C, et al. Application of a new approach methodology (NAM)-based strategy for genotoxicity assessment of data-poor compounds. Front Toxicol. 2023;5:1098432.
11. Buick JK, Williams A, Gagné R, Swartz CD, Recio L, Ferguson SS, et al. Flow cytometric micronucleus assay and TGx-DDI transcriptomic biomarker analysis of ten genotoxic and non-genotoxic chemicals in human HepaRG™ cells. Genes Environ. 2020;42(1):5.
12. Buick JK, Williams A, Meier MJ, Swartz CD, Recio L, Gagné R, et al. A Modern Genotoxicity Testing Paradigm: Integration of the High-Throughput CometChip® and the TGx-DDI Transcriptomic Biomarker in Human HepaRG™ Cell Cultures. Front Public Heal. 2021;9:694834.
13. Thienpont A, Cho E, Williams A, Meier MJ, Yauk CL, Rogiers V, et al. Unlocking the Power of Transcriptomic Biomarkers in Qualitative and Quantitative Genotoxicity Assessment of Chemicals. Chem Res Toxicol. 2024;37(3):465–75.
14. Corton JC, Witt KL, Yauk CL. Identification of p53 Activators in a Human Microarray Compendium. Chem Res Toxicol. 2019;32(9):1748–59.
15. Corton JC, Williams A, Yauk CL. Using a gene expression biomarker to identify DNA damage-inducing agents in microarray profiles. Environ Mol Mutagen. 2018;59(9):772–84.
16. OECD (1997), *Test No. 473: In vitro Mammalian Chromosome Aberration Test*, OECD Publishing, Paris, https://doi.org/10.1787/9789264071261-en.
17. OECD (2023), *Test No. 487: In Vitro Mammalian Cell Micronucleus Test*, OECD Guidelines for the Testing of Chemicals, Section 4, OECD Publishing, Paris, https://doi.org/10.1787/9789264264861-en

APPENDIX I. STATISTICS

Laboratories should use the TGx-DDI Biomarker for DNA Damage Classification Tool (https://cebs.niehs.nih.gov/tgxddi/tool) for making a classification call. The software tools only require the user to click on an analysis button for the full TGx-DDI biomarker analysis to be executed. The software tool also creates the call for the user (described in Section 'Data Interpretation').

Nearest Shrunken Centroids Probability Analysis

The probability analysis uses the class probabilities and discriminant functions outlined in Tibshirani et al. (2002). A detailed, documented step-by-step approach applying the methodology to obtain the nearest shrunken centroids along with the accompanying R Code and data are presented in the Data Brief article by Williams et al. (2015). Given the shrunken centroids, the probability for a new test agent, x*, is estimated using the discriminant scores for the DNA damage- inducing (DDI) and non-DDI nearest shrunken centroid, x^k. The discriminant score is written as follows:

$$\delta_k(x^*) = \sum_{i=1}^{64} \frac{\left(x_i^* - x_i^k\right)}{(s_i - s_o)^2} - 2log(0.5)$$

where s_0 is a positive constant set to be the median of the within-class standard deviations, s_i, for all genes in the biomarker. The shrunken centroid coefficients, x_i^k, are presented in Table 1 of Williams et al. (2015) with the estimated standard deviations. The discriminant scores are used to construct the DDI probability estimate, by analogy to Gaussian linear discriminant analysis:

$$Prob(DDI) = \frac{exp\left(\frac{-1}{2}\delta_{DDI}(x^*)\right)}{exp\left(\frac{-1}{2}\delta_{DDI}(x^*)\right) + exp\left(\frac{-1}{2}\delta_{non-DDI}(x^*)\right)}$$

A positive DDI call is made if the estimated probability is > 90% and a non-DDI call is made if the estimated probability is < 10%; otherwise, the call is ‘inconclusive’. The 90% and 10% thresholds are selected and not data derived.

Principal Component Analysis

The PCA call for a new test agent is conducted in the Manticore tool by estimating the first principal component using the loadings determined from the training set. In the PCA, the correlation matrix is used in estimating the principal components. In this analysis, the DDI or non-DDI call is made based on the sign of the estimated principal component. If the first principal component is <0, a DDI call is given. Similarly, if the value is > 0, a non-DDI call is made. The online tool provides this analysis as a scatterplot of the first and second principal components. A red vertical line at 0 for the first principal component is displayed to aid the user with regard to making the call.

Please see below for the equation of PC1 to be used for PCA analysis of the test compounds.

Gene	Mean	Standard Deviation	PCA Loading	Gene	Mean	Standard Deviation	PCA Loading
ACTA2	0.086	0.131	-0.093	HIST1H3D	0.0269	0.1551	0.0913
AEN	0.071	0.207	-0.152	ID2	0.0641	0.1926	0.1109
ARRDC4	0.111	0.196	-0.129	IKBIP	0.0513	0.1558	-0.1383
B3GNT2	-0.006	0.188	0.113	ITPKC	0.0757	0.1007	-0.1347
BLOC1S2	0.107	0.170	-0.154	ITPR1	0.0060	0.1722	0.1048
BRMS1L	0.078	0.136	-0.125	LCE1E	0.1403	0.2682	-0.1321
BTG2	0.187	0.189	-0.140	LRRFIP2	0.0265	0.1281	0.1123
C12orf5	0.119	0.172	-0.140	MDM2	0.1470	0.1780	-0.1422
CBLB	-0.022	0.125	0.129	MEX3B	0.1100	0.1435	-0.0932
CCP110	0.064	0.117	-0.152	NLRX1	-0.0273	0.1227	-0.1214
CDKN1A	0.247	0.203	-0.121	PCDH8	0.1902	0.2420	-0.1437
CEBPD	-0.113	0.242	-0.101	PHLDA3	0.1160	0.2117	-0.1601
CENPE	-0.059	0.137	0.110	PLK3	0.0641	0.2312	-0.1126
COIL	-0.086	0.179	-0.107	PPM1D	0.1801	0.1959	-0.1510
DAAM1	0.085	0.189	0.119	PRKAB1	0.1538	0.2269	-0.1583
DCP1B	0.066	0.123	-0.153	PRKAB2	0.0737	0.1283	-0.1331
DDB2	0.114	0.155	-0.157	PTGER4	0.0523	0.1954	0.1185
DUSP14	0.099	0.107	-0.131	RAPGEF2	0.0214	0.1611	0.1261
E2F7	0.100	0.172	-0.151	RBM12B	-0.0296	0.1165	-0.1323
E2F8	-0.055	0.202	-0.128	RPS27L	0.0897	0.1147	-0.1330
EI24	0.021	0.103	-0.151	RRM2B	0.1712	0.2116	-0.1290

FAM123B	-0.120	0.168	-0.139	SEL1L	0.0353	0.1360	0.0816
FBXO22	0.045	0.111	-0.147	SEMG2	0.0719	0.1428	-0.1118
GADD45A	0.261	0.202	-0.111	SERTAD1	0.2469	0.2590	-0.1260
GXYLT1	0.045	0.068	-0.117	SMAD5	0.0580	0.1283	-0.1337
HIST1H1E	-0.128	0.295	0.081	TM7SF3	0.0535	0.1115	-0.1444
HIST1H2BB	0.014	0.226	0.076	TNFRSF17	-0.0879	0.2837	-0.1101
HIST1H2BC	0.009	0.245	0.087	TOPORS	-0.0478	0.1523	-0.1181
HIST1H2BG	0.029	0.279	0.085	TP53I3	0.0637	0.1498	-0.1483
HIST1H2BI	0.011	0.236	0.076	TRIAP1	0.0421	0.2183	-0.1482
HIST1H2BM	-0.002	0.263	0.075	TRIM22	0.0865	0.2124	-0.1517
HIST1H2BN	0.004	0.118	0.081	USP41	0.0346	0.0876	-0.0529

$$PC1 = \sum_{Gene\,=\,1}^{64} PC1\ Loading_{Gene} \frac{\left(log2FC_{gene} - Mean_{gene}\right)}{Std.Dev_{Gene}}$$

Hierarchical Cluster Analysis

The inconclusive chemical could either cluster with the DDI agents (DDI call) or non-DDI (non-DDI call) agents or cluster on its own as a singleton cluster (inconclusive).

Any positive call (probability > 0.9 of DDI by probability analysis OR clusters with DDI agents by HCA OR has a negative PC1 in the PCA) leads to a positive DDI call. If the classification is not clear, then the inconclusive chemical may be a borderline case or there may be an outlier for at least one of the genes in the biomarker. If the classification is not a clear DDI or non-DDI call, then the experiment should be repeated.

These tools do not provide confidence intervals. We make DDI or non-DDI calls only. However, we have a high degree of confidence in the non-DDI calls under those biological conditions. Probability analysis derives an estimated probability of being non-DDI (<10% probability of being DDI) or DDI (>90%). Anything in between is inconclusive. Anything inconclusive will warrant further scrutiny. Classification would then be based on PCA and HCA.

References Appendix I

Tibshirani R, Hastie T, Narasimhan B, and Chu G. Diagnosis of multiple cancer types by shrunken centroids of gene expression. Proc Natl Acad Sci U S A. 2002;99:6567–6572.

Williams A, Buick JK, Moffat I, Swartz CD, Recio L, Hyduke DR, Li HH, Fornace AJ, Jr., Aubrecht J, and Yauk CL. A predictive toxicogenomics signature to classify genotoxic versus non-genotoxic chemicals in human TK6 cells. Data Brief. 2015;5:77–83.
